# Preparation and Transformations of Acetophenone-Derived Enamino Ketones, BF_2_-β-Ketoiminates, and BF_2_-β-Diketonates

**DOI:** 10.3390/molecules30030601

**Published:** 2025-01-29

**Authors:** Helena Brodnik, Luka Ciber, Uroš Grošelj, Nejc Petek, Bogdan Štefane, Jurij Svete

**Affiliations:** Faculty of Chemistry and Chemical Technology, University of Ljubljana, Večna pot 113, 1000 Ljubljana, Slovenia; helena.brodnik@fkkt.uni-lj.si (H.B.); luka.ciber@fkkt.uni-lj.si (L.C.); uros.groselj@fkkt.uni-lj.si (U.G.); bogdan.stefane@fkkt.uni-lj.si (B.Š.)

**Keywords:** enaminones, BF_2_-ketoiminates, BF_2_-diketonates, transamination, photochemistry, De Mayo reaction, X-ray diffraction

## Abstract

A series of differently substituted β-enaminones **2a**,**b**, **4a**–**i**, **8a**–**d**, and **9**–**13**, their BF_2_-β-ketoiminate complexes **5a**–**d**, and BF_2_-β-diketonate complexes **6a**–**d** were prepared as model substrates for photochemical transformations. The attempted photochemical transformations of enaminones **2**, **4**, **8** and BF_2_-β-ketoiminate complexes **5** failed. On the other hand, irradiation of mixtures of BF_2_-β-diketonate complexes **6a**–**d** and cycloalkanes with UV-A light (365 nm) gave the corresponding De Mayo reaction products **7a**–**f** in 9–30% yields. The photochemical ring-expansion of acetyl tetralone-derived BF_2_-complex **6d** gave novel diannulated cyclooctane derivatives **7e** and **7f**, which would be difficult to obtain using conventional cyclization methods.

## 1. Introduction

Enaminones are versatile reagents and building blocks in organic synthesis. Structurally, they are regarded as the enamino equivalents of 1,3-dicarbonyl compounds, as well as the aza-equivalents of enols. Accordingly, these simple and versatile C_3_-building blocks can react as 1,3-dielectrophiles at terminal carbon atoms and/or as nucleophiles at the central carbon atom. Since their synthetic potential was recognized a long time ago, enaminones have a long record of applications, not only in organic and heterocyclic chemistry, but also in other chemistry-related fields and their use in various applications continues. Since the first general review on enaminones by Greenhill in 1977 [1], several reviews on different aspects of chemistry of enaminones have been published [2,3,4,5,6,7,8].

In contrast to well-elaborated thermal transformations, photochemical transformations of enaminones have been less studied. The majority of classical photochemical reactions of enaminones are [2+2] cycloadditions, 6π-electrocyclizations, and ene-type transformations, which have been used in the synthesis of saturated nitrogen heterocycles, indoles, natural products, and their analogues [9,10,11]. Recently, the array of photochemical reactions of enaminones was expanded by photocatalytic transformations, such as α-C–H selenylation, thiocyanation, and perfluoroalkylation. These transformations were also used in the synthesis of 1,2-diketones, β-keto esters, and annulation reactions to obtain five- and six-membered heterocycles [10].

BF_2_-β-diketonates are the cyclic equivalents of 1,3-diketones locked in their enol form. Coordination of β-diketone to Lewis acid (BF_3_·Et_2_O) enhances the electrophilic character of the terminal carbon atoms, while retaining a mild nucleophilic character at the central carbon atom. Similarly, BF_2_-β-ketoiminates are also available from β-enaminones and boron trifluoride etherate. Boron β-diketonates and β-ketoiminates are used as reagents in organic synthesis [12,13,14]. However, they are much better known for interesting optical properties, such as fluorescence, room temperature phosphorescence, and aggregation-induced emission enhancement (AIEE). Therefore, boron β-diketonates and β-ketoiminates are interesting as materials for various optoelectronic applications [15,16,17,18,19,20,21,22,23,24].

In continuation of our long-term interest in the chemistry of enaminones and BF_2_-β-diketonates and their use in organic synthesis, we recently turned our attention to the application of enaminones as ligands and/or building blocks in transition-metal catalysis [25], in asymmetric organocatalysis [26], and in the synthesis of polyenaminones as recyclable optical- and redox-active materials [27]. By extension, we became interested in using enaminones, BF_2_-β-ketoiminates, and BF_2_-β-diketonates as substrates in photochemical and photocatalytic transformations. Therefore, we prepared some model enaminones, BF_2_-β-ketoiminates, and BF_2_-β-diketonates and used them as substrates for further photochemical studies. Herein, we report the results of this study, the synthesis and transformations of selected α-unsubstituted enamino ketones and their BF_2_-complexes, and the photochemical transformations (De Mayo reactions) of BF_2_- β-diketonates.

## 2. Results

### 2.1. Synthesis of Model Enamino Ketones ***4***

**Scheme 1:**
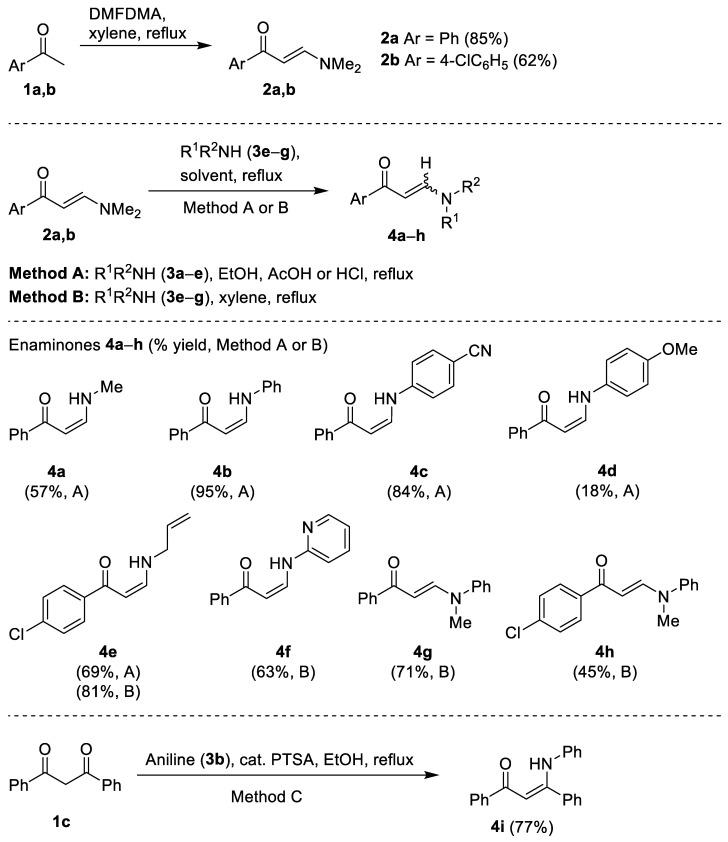
Synthesis of 1-aryl-3-(substituted amino)propen-1-ones **4a**–**h** and (*Z*)-1,3-diphenyl-3-(phenylamino)prop-2-en-1-one (**4i**).

(*E*)-3-(dimethylamino)-1-phenylprop-2-en-1-one (**2a**) and its 4′-chloro analogue **2b** were prepared in two steps from acetophenone (**1a**) and 4-chloroacetophenone (**1b**) by heating with 1.1 equiv. of *N,N*-dimethylformamide dimethylacetal (DMFDMA) in anh. xylene for 16 h to afford the corresponding *N,N*-dimethylenaminones **2a** and **2b** in 85% and 62% yield, respectively. Compounds **2a** and **2b** were then heated with l.1 equiv. of primary amines **3a**–**e** in ethanol in the presence of acetic acid or hydrochloric acid to give the secondary enaminones **4a**–**e** in 18–94% yields (Scheme 1, Method A). On the other hand, acid-catalyzed transamination of *N,N*-dimethylenaminones **2** did not take place with 2-aminopyridine (**3f**) and *N*-methylaniline (**3g**). Transamination products **4f**, **4g**, and **4h** were then obtained by heating **2a** and **2b** with excess **3f** and **3g** in toluene or xylene. In the same way, the secondary enaminone **4e** was also obtained in 81% yield from enaminone **2b** and excess allylamine (**3e**) (Scheme 1, Method B). Finally, β-anilinochalcone **4i** was prepared by acid-catalyzed condensation of dibenzoylmethane (**1c**) and aniline (**3b**) (Scheme 1, Method C). Experimental data for compounds **2** and **4** are summarized in Table 1.

### 2.2. Synthesis and Photochemical Reactions of BF_2_-β-Ketoiminates ***5*** and BF_2_-β-Diketonates ***6***

**Scheme 2:**
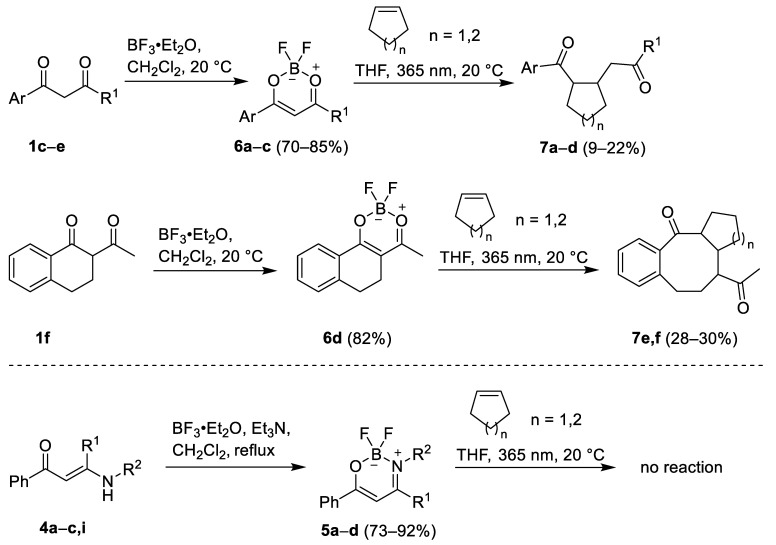
Synthesis of boron β-ketoiminate complexes **5a**–**d**, boron β-diketonate complexes **6a**–**d** and De Mayo reaction products **7a**–**f**.

Treatment of secondary enaminones **4a**–**c,i** with BF_3_·Et_2_O in the presence of triethylamine produced the corresponding BF_2_-β-ketoiminate complexes **5a**–**d** in 73–92% yields. Similarly, boron β-diketonates **6a**–**d** were prepared from 1,3-diketones **1c**–**f** and BF_3_·Et_2_O in dichloromethane following the literature procedure [28]. BF_2_-β-diketonate complexes **6** reacted readily with cyclopentene and cyclohexene under irradiation (365 nm) in dichloromethane to furnish the corresponding cycloalkane insertion products **7a**–**f** in 9–30% yields. Gratifyingly, novel compounds **7e** and **7f** are nice examples of a ring-expansion reaction furnishing cyclooctane ring, which is difficult to obtain using conventional cyclization methods (Scheme 2). On the other hand, attempts to carry out De Mayo reaction by irradiation of mixtures of β-ketoiminate complexes **5a**–**d** and various alkenes with UV-A light (365 nm) failed. This is in line with, to our knowledge, the only literature report on De Mayo reaction using BF_2_-β-ketoiminates as substrates [29]. Experimental data for compounds **5**–**7** are summarized in Table 2.

### 2.3. Other Transformations of Model Enamino Ketones ***4***

**Scheme 3:**
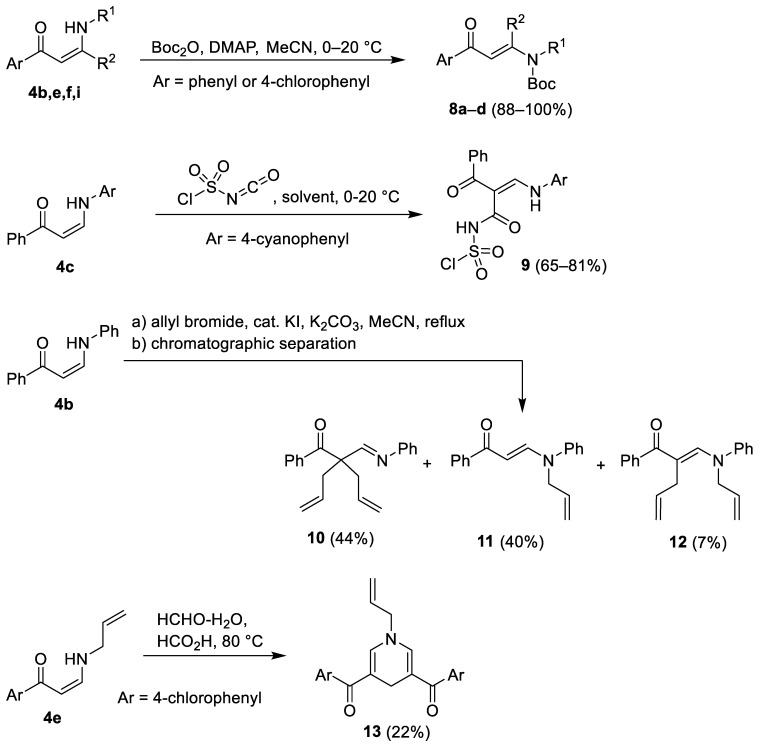
Other transformations of enaminones **4b**,**c**,**e**,**f**,**i**. Synthesis of *N*-Boc-enaminones **8a**–**d**, (*Z*)-{2-benzoyl-3-[(4-cyanophenyl)amino]acryloyl}sulfamoyl chloride (**9**), allylation products **10**–**12**, and 1,4-dihydropyridine derivative **13**.

As the initially prepared substrates **4** and **5** failed to produce the desired cycloalkane insertion products, additional derivatives of secondary enaminones were prepared and tested for photochemical conversion. Compounds **4b**,**e**,**f**,**i** were acylated with di-*tert*-butyl dicarbonate (Boc_2_O) in acetonitrile in the presence of 4-(dimethylamino)pyridine (DMAP) to afford the corresponding *N*-Boc derivatives **8a**–**d** in 88–100% yields. On the other hand, reaction of **4c** with chlorosulfonyl isocyanate (CSI) in Et_2_O at 0–20 °C furnished (*Z*)-{2-benzoyl-3-[(4-cyanophenyl)amino]acryloyl}sulfamoyl chloride (**9**) as the *C*-acylation product in 80% yield. Replacement of Et_2_O with toluene and acetonitrile as solvents gave **9** in 81% and 65% yield, respectively. Somewhat surprisingly, treatment of **4b** with excess allyl bromide in the presence of K_2_CO_3_ gave a mixture of three products **10**–**12**. Subsequent chromatographic separation afforded the *C*,*C*-diallylation product **10** in 44% yield, the *N*-monoallylation product **11** in 40% yield, and the *C*,*N*-diallylation product **12** in 7% yield. Attempted methylation of **4e** with aq. formaldehyde in formic acid did not give any of the expected methylation products. Instead, 1-allyl-3,5-bis(4-chlorobenzoyl)-1,4-dihydropyridine (**13**) was isolated in 22% yield (Scheme 3). Experimental data for compounds **8**–**13** are summarized in Table 3.

**Scheme 4:**
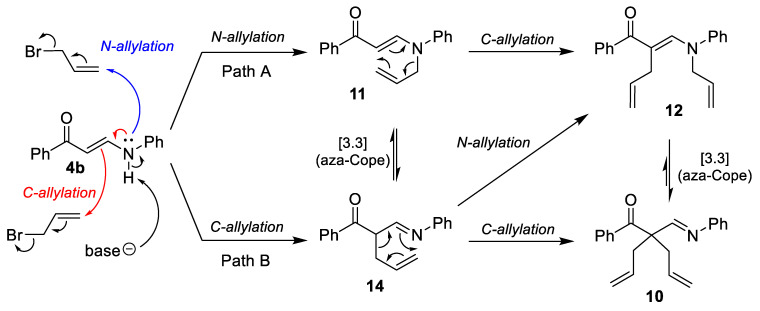
The proposed reaction pathway for the formation of compounds **10**–**12**.

Formations of the products **4**–**9** (cf. Scheme 1, Scheme 2 and Scheme 3) were expected and are explainable by general mechanisms for enaminone transamination (compounds **4**), boron β-ketoiminate and β-diketonate complex formation (compounds **5** and **6**), De Mayo reaction (compounds **7**), acylation (compounds **8**), and electrophilic substitution (compound **9**). On the other hand, formation of a mixture of three allylation products **10**–**12** and dihydropyridine derivative **13** were outside the expected product formation and require some further discussion. The proposed reaction pathway leading to a mixture of **10**–**12** is shown on Scheme 4. Reaction of enaminone **4b** with 2 equiv. allyl bromide can take place as initial *N*-allylation (Path A) and/or *C*-allylation (Path B) to give the respective monoallylated compounds **11** and/or **14**. Further allylation of the *N*-allyl intermediate **11** can only take place at the carbon atom to give the *C*,*N*-diallylated compound **10**, while allylation of the *C*-allyl intermediate **14** can give two products, the *C*,*C*-diallylated product **10** and the *C*,*N*-diallylated compound **12**. Finally, the allyl group in monoallylated compounds **11** and **14** and in diallylated compounds **10** and **12** can migrate from carbon to nitrogen and vice versa via a [3.3] sigmatropic rearrangement (aza-Cope rearrangement) [30,31,32] as proposed in Scheme 4.

**Scheme 5:**
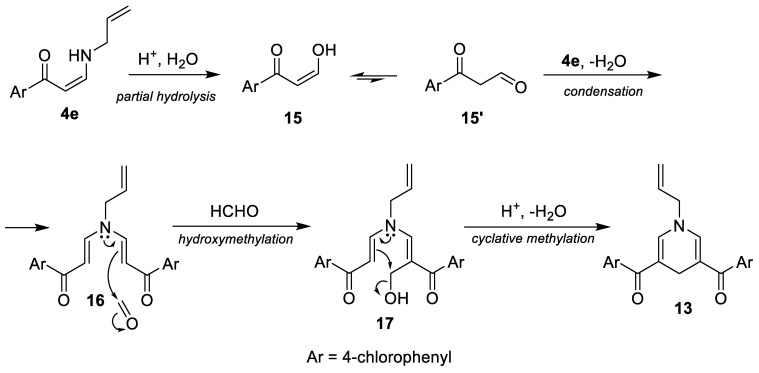
Plausible explanation for the formation of compound **13**.

A plausible explanation for the formation of 1,4-dihydropyridine derivative **13** is shown in Scheme 5. Under aqueous acidic conditions, the enamine moiety of *N*-allylenaminone **4e** partially hydrolyzes to give the β-keto aldehyde intermediate **15/15′**, which reacts with enaminone **4e** to afford the bis-enaminone intermediate **16**. Subsequent *C*-hydroxymethylation of **16** with formaldehyde gives the intermediate **17**, which undergoes acid-catalyzed cyclative alkylation via elimination of water to furnish the 1,4-dihydropyridine derivative **13**.

Unfortunately, the attempted photochemical and photocatalytic transformations of compounds **4** and **8** were unsuccessful. In most cases, no reaction took place, while in some cases, complex inseparable mixtures of products were obtained.

## 3. Structure Determination and Compound Characterization

The structures of compounds **4**–**13** were determined by spectroscopic methods (^1^H, ^13^C NMR, IR, HRMS, and UV-vis) and by elemental analyses for C, H, and N. The structures of compounds **5b**, **8a**, **8c**, and **8d** (Figures S2–S5) in the solid state were determined by X-Ray diffraction.

**Figure 1:**
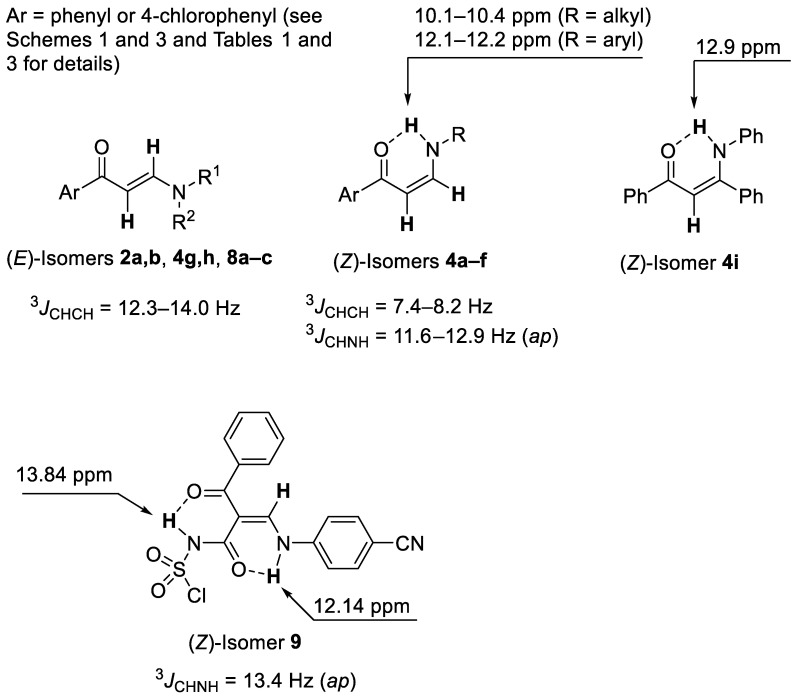
The structures of enaminones **2**, **4**, **8**, and **9** in CDCl_3_ solution determined by NMR.

In solution, enaminones can exist as mixtures of the *E*- and the *Z*-isomers and the predominance of either isomer depends on the structure of enaminone and the solvent [1,2,3,4,5,6,7,8]. NMR spectra of tertiary enaminones **2a**,**b**, **4g**,**h**, and **8a**–**c** in CDCl_3_ exhibited a large vicinal coupling constant, ^3^*J*_CHCH_ = 12.3–14.0 Hz, which was in agreement with the *E*-configuration around the C=C double bond. On the other hand, the NMR spectra of secondary enaminones **4a**–**f** in CDCl_3_ exhibited smaller vicinal coupling constant, ^3^*J*_CHCH_ = 7.4–8.2 Hz, which was in agreement with the *Z*-configuration around the C=C double bond. The *Z*-configuration of **4a**–**f** in CDCl_3_ is favored by the intramolecular N–*H*···*O*=C hydrogen bond, which is consistent with the observed chemical shift, *δ* = 10.1–12.2 ppm, for the NH proton. On the basis of the large *δ* chemical shift around 12.5 ppm, the *Z*-configuration around the C=C double bond was also assigned to compounds **4i** and **9**. Furthermore, the chemical shift of the sulfonamide NH proton, *δ* = 13.9 ppm, supports the intramolecular N–*H*···*O*=C hydrogen bond between the sulfonamide and the benzoyl groups attached to the α-carbon atom of **9** (Figure 1).

The absorption spectra of compounds **4**, **5**, **8**, and **9** are shown in Figure S6. Absorption spectra of *N*-aryl-substituted enaminones **4b**–**d**,**f**,**g**,**i** show typical absorption maxima between 350 nm (**4g**, purple line) and 380 nm (**4d**, blue line) (Figure S6A). Absorption spectra of oxazaborinines **5** are similar to those of parent enaminones **4**; the *N*-methyloxazaborinine **5a** absorbs at 334 nm (brown line), while the *N*-aryl analogues **5b**–**d** absorb between 356 nm (**5d**, green line) and 373 nm (**5c**, blue line) (Figure S6B). N-acylation of **4** into product **8** resulted in the blue-shift of absorption maxima, which appear between 300 nm (**8c**, red line) and 327 nm (**8d**, blue line) (Figure S6C). Compound **9** with chlorosulfonylaminocarbonyl group at α-position exhibits two distinct absorption maxima at 267 nm and 360 nm (Figure S6D). In summary, the *N*-aryl-substituted enaminones **4b**–**d**,**f**,**g**,**i** and **9** and the respective BF_2_-complexes **5b**–**d** absorb visible light up to 415–470 nm, while the *N*-Boc-analogues **8a**,**c**,**d** and *N*-methyloxazaborinine **5a** absorb only UV-A light below 400 nm (Figure S6).

## 4. Experimental

### 4.1. General Methods

Melting points were determined on a Kofler micro hot stage and on a Mettler Toledo MP30 automated melting point system (Mettler Toledo, Columbus, OH, USA). The NMR spectra were recorded in CDCl_3_ and DMSO-d_6_ using Me_4_Si as the internal standard on a Bruker Avance III Ultrashield 500 and Bruker Avance Neo 600 instruments (Bruker, Billerica, MA, USA) at 500 and 600 MHz for ^1^H and at 125 and 150 MHz for ^13^C nucleus, respectively. Chemical shifts (δ) are given in ppm relative to Me_4_Si as internal standard (δ = 0 ppm) and vicinal coupling constants (*J*) are given in hertz (Hz). HRMS spectra were recorded on an Agilent 6224 time-of-flight (TOF) mass spectrometer equipped with a double orthogonal electrospray source under atmospheric pressure ionization (ESI) coupled to an Agilent 1260 high-performance liquid chromatograph (HPLC) (Agilent Technologies, Santa Clara, CA, USA). UV-vis spectra were recorded in MeOH using a Varian Cary Bio50 UV–Visible Spectrophotometer (Agilent Technologies, Santa Clara, CA, USA). Emission spectra were recorded on a PerkinElmer LS 50 B Luminescence spectrophotometer (PerkinElmer, Waltham, MA, USA). Fourier-transform infrared (FT-IR) spectra were obtained on a Bruker FTIR Alpha Platinum spectrophotometer (Bruker, Billerica, MA, USA) using attenuated total reflection (ATR) sampling technique. Microanalyses for C, H, and N were obtained on a Perkin-Elmer CHNS/O Analyzer 2400 Series II (PerkinElmer, Waltham, MA, USA). Column chromatography (CC) was performed on silica gel (Silica gel 60, particle size: 0.035–0.070 mm (Sigma-Aldrich, St. Louis, MO, USA)). Photochemical transformations were performed on a Penn PhD Photoreactor M2 with LED light source (3 W, 365 nm) and air-cooling (Penn Photon Devices, Pennsburg, PA, USA).

Acetophenones **1a** and **1b**, 1,3-diketones **1c**–**f**, *N,N*-dimethylformamide dimethylacetal (DMFDMA), amines **3a**–**g**, acetic acid, 37% hydrochloric acid, di-*tert*-butyl dicarbonate (Boc_2_O), chlorosulfonyl isocyanate (CSI), allyl bromide, 37% aqueous formaldehyde, formic acid, cyclopentene, cyclohexene, triethylamine, and BF_3_·Et_2_O are commercially available (Sigma-Aldrich, St. Louis, MO, USA).

### 4.2. General Procedure for the Synthesis of N,N-Dimethylenaminones ***2a*** and ***2b***

*N,N*-Dimethylformamide dimethyl acetal (DMFDMA) (1.5 mL, 11.2 mmol) was added to a solution of acetophenone derivative **1** (1.20 g, 10 mmol) in anh. xylene (10 mL) and the mixture was refluxed under argon for 16 h. Volatile components were evaporated in vacuo and the orange–red solid residue was triturated with petroleum ether (20 mL). The precipitate was collected by filtration, washed with petroleum ether (2 × 5 mL), and air-dried to give **2**.

#### 4.2.1. (*E*)-3-(Dimethylamino)-1-phenylprop-2-en-1-one (**2a**)

Prepared from acetophenone (**1a**) (1.20 g, 10 mmol) and DMFDMA (1.5 mL, 11.2 mmol). Yield: 1.49 g (85%) of ochre solid, m.p. 93–94 °C, lit. [33] m.p. 90–92 °C. ^1^H NMR (500 MHz, CDCl_3_) δ 7.89 (dt, *J* = 6.9, 1.5 Hz, 2H), 7.80 (d, *J* = 12.4 Hz, 1H), 7.48–7.36 (m, 3H), 5.71 (d, *J* = 12.4 Hz, 1H), 3.14 and 2.92 (2 br s, 1:1, 6H). ^13^C NMR (126 MHz, CDCl_3_) δ 188.6, 154.2, 140.5, 130.8, 128.1, 127.5, 92.2, 45.0, 37.2. *ν*_max_ 2905, 2805, 1633, 1581, 1428, 1362, 1273, 1051 cm^−1^; ESI-HRMS: *m*/*z* = 176.1068 [M+H]^+^, C_11_H_14_NO requires *m*/*z* = 176.1070. Anal. Calcd. for C_11_H_13_NO: C, 75.40; H, 4.93; N, 7,90%. Found: C, 75.59; H, 4.93, N, 7.90%. Physical and spectral data are in agreement with the literature data [33,34,35].

#### 4.2.2. (*E*)-1-(4-Chlorophenyl)-3-(dimethylamino)prop-2-en-1-one (**2b**)

Prepared from 4-chloroacetophenone (**1b**) (1.55 g, 10 mmol) and DMFDMA (1.5 mL, 11.2 mmol). Yield: 1.295 g (62%) of light orange solid, m.p. 87–88 °C, lit. [34] m.p. 85–87 °C. ^1^H NMR (500 MHz, CDCl_3_) δ 7.87–7.81 (m, 2H), 7.80 (d, *J* = 12.3 Hz, 1H), 7.40–7.34 (m, 2H), 5.66 (d, *J* = 12.3 Hz, 1H), 3.16 and 2.93 (2 br s, 1:1, 6H). Physical and spectral data are in agreement with the literature data [34].

### 4.3. General Procedures for the Synthesis of Enaminones ***4a***–***h***

General Procedure A (G.P.A).

A mixture of *N,N*-dimethylenaminone **2** (10 mmol), amine **3** (10 mmol), ethanol (40 mL), and acetic acid (1.5 mL, 25 mmol) or 37% hydrochloric acid (1 mL, 10 mmol), was stirred under reflux for 3–16 h. The reaction mixture was cooled to 0 °C (ice bath), the precipitate was collected by filtration and washed with EtOH–water (1:1, 2 × 5 mL) to give compound **4**.

General Procedure B (G.P.B).

A two-necked 50 mL flask was charged with *N,N*-dimethylenaminone **2** (5 mmol), anh. xylene (25 mL), and amine **3** (5 mmol). The flask was then equipped with a reflux condenser and nitrogen inlet adapter. The reaction mixture was stirred under reflux and dry nitrogen was slowly bubbled into the reaction mixture to assist the removal of dimethylamine, evolution of which was monitored by indication with wet litmus paper at the top of reflux condenser. After approximately 12 h, the evolution of dimethylamine ceased (negative wet-litmus test) indicating completion of transamination reaction. Volatile components were evaporated in vacuo and the residue was purified by CC. Fractions containing the product were combined and evaporated in vacuo to give compound **4**.

#### 4.3.1. (*Z*)-3-(methylamino)-1-phenylprop-2-en-1-one (**4a**)

Prepared from enaminone **2a** (1.754 g, 10 mmol) and methylamine hydrochloride (**3a**) (1.35 g, 20 mmol), G.P.A, reflux for 16 h. Addition of acetic acid or hydrochloric acid was omitted and the product **4a** did not precipitate for the reaction mixture. Volatile components were evaporated in vacuo and the residue was purified by CC (EtOAc–hexanes, 1:5). Fractions containing the product were combined and evaporated in vacuo to give compound **4a**. Yield: 912 mg (57%) of yellow solid, m.p. 135–137 °C, lit. [36] m.p. 138–139 °C. ^1^H NMR (500 MHz, CDCl_3_) δ 10.13 (s, 1H), 7.83–7.74 (m, 2H), 7.45–7.27 (m, 3H), 6.83 (dd, *J* = 12.9, 7.4 Hz, 1H), 5.61 (d, *J* = 7.3 Hz, 1H), 2.99 (d, *J* = 5.1 Hz, 3H). *ν*_max_ 3071, 2948, 1630 (C=O), 1512, 1422, 1395, 1286, 1058 cm^−1^. Physical and spectral data are in agreement with the literature data [36,37].

#### 4.3.2. (*Z*)-1-Phenyl-3-(phenylamino)prop-2-en-1-one (**4b**)

Prepared from enaminone **2a** (1.754 g, 10 mmol) and aniline hydrochloride (**3b**) (1.30 g, 10 mmol), G.P.A, reflux for 3 h. Yield: 2.111 g (95%) of yellow solid, m.p. 139–140 °C, lit. [38] m.p. 141 °C. ^1^H NMR (500 MHz, CDCl_3_) δ 12.15 (d, *J* = 12.3 Hz, 1H), 7.98–7.92 (m, 2H), 7.57–7.42 (m, 4H), 7.39–7.32 (m, 2H), 7.15–7.06 (m, 3H), 6.04 (d, *J* = 7.8 Hz, 1H). ^13^C NMR (126 MHz, CDCl_3_) δ 191.0, 145.0, 140.2, 139.2, 131.6, 129.8, 128.5, 127.3, 123.7, 116.4, 93.7. *ν*_max_ 3050, 1659 (C=O), 1542, 1472, 1268, 1239, 1175, 886 cm^−1^. ESI-HRMS: *m*/*z* = 224.1082 [M+H]^+^, C_15_H_14_NO requires *m*/*z* = 224.1070. Physical and spectral data are in agreement with the literature data [38,39].

#### 4.3.3. (*Z*)-4-[(3-Oxo-3-phenylprop-1-en-1-yl)amino]benzonitrile (**4c**)

Prepared from enaminone **2a** (526 mg, 3 mmol), 4-aminobenzonitrile (**3c**) (390 mg, 3.3 mmol), and acetic acid (1.5 mL), G.P.A, reflux for 16 h. Yield: 622 mg (84%) of yellow solid, m.p. 153–154 °C, lit. [40] m.p. of the (*E*)-isomer 209–210 °C. ^1^H NMR (500 MHz, CDCl_3_) δ 12.18 (d, *J* = 11.9 Hz, 1H), 7.98–7.91 (m, 2H), 7.66–7.59 (m, 2H), 7.58–7.50 (m, 1H), 7.53–7.43 (m, 3H), 7.17–7.08 (m, 2H), 6.17 (d, *J* = 8.1 Hz, 1H). ^13^C NMR (126 MHz, CDCl_3_) δ 191.9, 144.0, 142.6, 138.5, 134.0, 132.3, 128.6, 127.5, 118.9, 116.0, 106.0, 96.3. *ν*_max_ 3056, 2223 (C≡N), 1643 (C=O), 1471, 1287, 1243, 1176, 1019 cm^−1^; ESI-HRMS: *m*/*z* = 249.1015 [M+H]^+^, C_16_H_13_N_2_O requires *m*/*z* = 249.1022. Anal. Calcd. for C_16_H_12_N_2_O: C, 77.40; H, 4.87; N, 11.28%. Found: C, 77.04; H, 4.93, N, 10.90%. Physical and spectral data are in agreement with the literature data [40].

#### 4.3.4. (*Z*)-3-[(4-Methoxyphenyl)amino]-1-phenylprop-2-en-1-one (**4d**)

Prepared from enaminone **2a** (175 mg, 1 mmol), 4-methoxyaniline (**3d**) (123 mg, 1 mmol), and acetic acid (0.5 mL), G.P.A, reflux for 16 h. The product was purified by CC (Et_2_O). Fractions containing the product were combined and evaporated in vacuo to give compound **4d**. Yield: 45 mg (18%) of yellow solid, m.p. 146–148 °C, lit. [41] 145–147 °C. ^1^H NMR (500 MHz, CDCl_3_) δ 12.20 (d, *J* = 12.4 Hz, 1H), 7.97–7.90 (m, 2H), 7.53–7.40 (m, 4H), 7.09–7.02 (m, 2H), 6.93–6.86 (m, 2H), 5.98 (d, *J* = 7.7 Hz, 1H), 3.80 (s, 3H). ^13^C NMR (126 MHz, CDCl_3_) δ 190.6, 156.4, 145.9, 139.3, 133.8, 131.4, 128.4, 127.2, 117.9, 115.0, 92.9, 55.6. *ν*_max_ 3060, 2990, 1624 (C=O), 1470, 1290, 1254, 1179, 1030 cm^−1^. ESI-HRMS: *m*/*z* = 254.1176 [M+H]^+^, C_16_H_16_NO_2_ requires *m*/*z* = 254.1176. Physical and spectral data are in agreement with the literature data [41].

#### 4.3.5. (*Z*)-3-(Allylamino)-1-(4-chlorophenyl)prop-2-en-1-one (**4e**)

Prepared from enaminone **2b** (210 mg, 1 mmol), allylamine (**3e**) (90 μL, 1.2 mmol), and 37% aq. HCl (3 drops, 1 mmol), reflux for 16 h, G.P.A. The product **4e** did not precipitate for the reaction mixture. Volatile components were evaporated in vacuo and the residue was purified by CC (EtOAc–hexanes, 1:1). Fractions containing the product were combined and evaporated in vacuo to give compound **4e**. Prepared also from enaminone **2b** (2.10 g, 10 mmol) and allylamine (**3e**) (1.8 mL, 24 mmol) in anh. xylene (25 mL), G.P.B, CC (EtOAc–hexanes, 1:1). Yield: 152 mg (69%, G.P.A) and 1.796 g (81%, G.P.B) of brownish solid, m.p. 60–61 °C. ^1^H NMR (500 MHz, CDCl_3_) δ 10.35 (s, 1H), 7.81 (d, *J* = 8.6 Hz, 2H), 7.38 (d, *J* = 8.6 Hz, 2H), 6.95 (dd, *J* = 12.8, 7.5 Hz, 1H), 5.90 (ddt, *J* = 17.1, 10.4, 5.4 Hz, 1H), 5.69 (d, *J* = 7.4 Hz, 1H), 5.28 (dq, *J* = 17.2, 1.6 Hz, 1H), 5.22 (dq, *J* = 10.2, 1.4 Hz, 1H), 3.89 (tt, *J* = 5.7, 1.7 Hz, 2H). ^13^C NMR (126 MHz, CDCl_3_) δ 188.6, 154.4, 138.1, 137.0, 134.1, 128.5, 128.5, 117.3, 90.4, 51.1. *ν*_max_ 3278, 2909, 1533, 1274, 1087, 1006, 935, 847, 762 cm^−1^. ESI-HRMS: *m*/*z* = 222.0608 [M+H]^+^, C_12_H_13_ClNO requires *m*/*z* = 222.0677.

#### 4.3.6. (*Z*)-1-Phenyl-3-[(pyridin-2-yl)amino]prop-2-en-1-one (**4f**)

Prepared from enaminone 2a (876 mg, 5 mmol) and 2-aminopyridine (**3f**) (470 mg, 5 mmol) in anh. xylene (25 mL), G.P.B, CC (Et_2_O). Yield: 710 mg (63%) of yellow solid, m.p. 126–128 °C, lit. [42] m.p. 169–170 °C, lit. [43] m.p. 93 °C. ^1^H NMR (500 MHz, CDCl_3_) δ 12.16 (d, *J* = 11.6 Hz, 1H), 8.31 (ddd, *J* = 4.9, 1.9, 0.8 Hz, 1H), 8.26 (dd, *J* = 11.6, 8.2 Hz, 1H), 8.00–7.93 (m, 2H), 7.62 (ddd, *J* = 8.1, 7.3, 1.9 Hz, 1H), 7.56–7.48 (m, 1H), 7.50–7.43 (m, 2H), 6.95 (ddd, *J* = 7.3, 4.9, 0.9 Hz, 1H), 6.83 (dt, *J* = 8.2, 0.9 Hz, 1H), 6.15 (d, *J* = 8.2 Hz, 1H). ^13^C NMR (126 MHz, CDCl_3_) δ 191.9, 151.7, 148.5, 142.9, 139.0, 138.4, 131.8, 128.5, 127.5, 118.5, 111.7, 95.2. *ν*_max_ 3061, 1591, 1536, 1470, 1416, 1230, 1202, 1016 cm^−1^. ESI-HRMS: *m*/*z* = 225.1018 [M+H]^+^, C_14_H_13_N_2_O requires *m*/*z* = 225.1022. Physical and spectral data are in agreement with the literature data [42,43].

#### 4.3.7. (*E*)-3-[Methyl(phenyl)amino]-1-phenylprop-2-en-1-one (**4g**)

Prepared from enaminone **2a** (876 mg, 5 mmol) and *N*-methylaniline (**3g**) (470 mg, 5 mmol) in anh. xylene (25 mL), G.P.B, CC (Et_2_O). Yield: 840 mg (71%) of beige solid, m.p. 99–101°C, lit. [44] m.p. 110–112 °C. ^1^H NMR (500 MHz, CDCl_3_) δ 8.23 (d, *J* = 12.7 Hz, 1H), 7.98–7.91 (m, 2H), 7.53–7.46 (m, 1H), 7.48–7.41 (m, 2H), 7.42–7.34 (m, 2H), 7.25–7.14 (m, 3H), 6.10 (d, *J* = 12.7 Hz, 1H), 3.40 (s, 3H). ^13^C NMR (126 MHz, CDCl_3_) δ 189.41, 149.95, 146.4, 140.0, 131.4, 129.5, 128.3, 127.7, 124.9, 112.4, 96.9, 37.4. *ν*_max_ 3052, 3034, 1650 (C=O), 1539, 1491, 1260, 1176, 1020 cm^−1^. ESI-HRMS: *m*/*z* = 238.1222 [M+H]^+^, C_16_H_16_NO requires *m*/*z* = 238.1226. Anal. Calcd. for C_16_H_15_NO: C, 80.98; H, 6.37; N, 5.90%. Found: C, 80.59; H, 6.51, N, 5.98%. Physical and spectral data are in agreement with the literature data [44].

#### 4.3.8. (*E*)-1-(4-Chlorophenyl)-3-[methyl(phenyl)amino]prop-2-en-1-one (**4h**)

Prepared from enaminone **2b** (323 mg, 1.5 mmol) and *N*-methylaniline (**3g**) (400 μL, 3.7 mmol) in anh. xylene (5 mL), G.P.B, CC (Et_2_O). Yield: 183 mg (45%) of yellow solid, m.p. 119–120 °C, lit. [45] m.p. 119–120 °C. ^1^H NMR (500 MHz, CDCl_3_) δ 8.22 (d, *J* = 12.6 Hz, 1H), 7.89 (d, *J* = 8.5 Hz, 2H), 7.43–7.37 (m, 4H), 7.23–7.17 (m, 3H), 6.04 (d, *J* = 12.6 Hz, 1H), 3.41 (s, 3H). ^13^C NMR (126 MHz, CDCl_3_) δ 187.9, 150.4, 146.3, 138.3, 137.5, 129.6, 129.1, 128.5, 125.2, 120.6, 96.3, 37.2. *ν*_max_ 3065, 2916, 2198, 2163, 2051, 1923, 1675 (C=O), 1639 (C=O), 1572, 1539, 1497, 1466, 1426, 1392, 1347, 1326, 1305, 1267, 1208, 1182, 1158, 1137, 1087, 1056, 1030, 1009, 973, 901, 874, 845, 800, 758, 742, 713, 695, 679, 626, 610 cm^−1^. ESI-HRMS: *m*/*z* = 272.0835 [M+H]^+^, C_16_H_15_ClNO requires *m*/*z* = 272.0837. Physical and spectral data are in agreement with the literature data [45].

### 4.4. (Z)-1,3-Diphenyl-3-(phenylamino)prop-2-en-1-one (***4i***)

This compound was prepared by a modified literature procedure [46]. Dibenzoylmethane (**12b**) (11.2 g, 50 mmol) and *para*-toluenesulfonic acid monohydrate (476 mg, 2.5 mmol) were added to a solution of aniline (**3b**) (4.56 mL, 5 mmol) in ethanol (100 mL) and the mixture was stirred under reflux for 16 h. Water (50 mL) and charcoal (~1 g) were added, the mixture was stirred under reflux for 5 min, the suspension filtered hot, and the filtrate was left to stand at ~4 °C for 12 h. The precipitate was collected by filtration and re-crystallized from EtOH–H_2_O (2:1, 250 mL) to give pure compound **4i**. Yield: 11.50 g (77%) of yellow crystals, m.p. 102–103 °C, lit. [46] m.p. 102–103 °C, lit. [47] m.p. 144–145 °C, lit. [48] m.p. 101–102 °C. ^1^H NMR (500 MHz, CDCl_3_) δ 12.90 (s, 1H), 8.00–7.94 (m, 2H), 7.53–7.30 (m, 8H), 7.17–7.09 (m, 2H), 7.03–6.95 (m, 1H), 6.82–6.77 (m, 2H), 6.09 (s, 1H). ^13^C NMR (126 MHz, CDCl_3_) δ ^13^C NMR (126 MHz, CDCl_3_) δ 189.8, 161.6, 140.0, 139.6, 136.0, 131.4, 129.8, 128.9, 128.7, 128.5, 127.4, 124.3, 123.4, 97.2. *ν*_max_ 3019, 1591, 1555, 1510, 1319, 1284, 1215, 1053 cm^−1^. ESI-HRMS: *m*/*z* = 300.1380 [M+H]^+^, C_21_H_18_NO requires *m*/*z* = 300.1383. Physical and spectral data are in agreement with the literature data [46,47,48].

### 4.5. General Procedure for the Synthesis of β-Ketoiminate Complexes ***5a***–***d***

Compounds **5** a–d were prepared by a modified literature procedure [13,14]. Under argon, Et_3_N (140 µL, 1 mmol) and BF_3_·Et_2_O (370 µL, 3 mmol) were added subsequently to a stirred suspension of enaminone **4** (1 mmol) in anh. dichloromethane (5 mL) and the mixture was stirred under reflux under argon for 4–24 h.

Workup A: Volatile components were evaporated in vacuo and the residue was purified by CC (dry load). Fractions containing the product were combined and evaporated in vacuo to give **5**.

Workup B: Volatile components were evaporated in vacuo and the residue was triturated with Et_2_O (5 mL) and water (5 mL). The precipitate was collected by filtration and washed with Et_2_O (5 mL) to give **5**.

#### 4.5.1. 2,2-Difluoro-3-methyl-6-phenyl-2*H*-1,3λ^4^,2λ^4^-oxazaborinine (**5a**)

Prepared from enaminone **4a** (162 mg, 1 mmol), Et_3_N (140 µL, 1 mmol), and BF_3_·Et_2_O (370 µL, 3 mmol), reflux for 24 h, Workup A, CC (CH_2_Cl_2_). Yield: 164 mg (78%) of beige solid, m.p. 128–129 °C. ^1^H NMR (500 MHz, CDCl_3_) δ 7.96–7.89 (m, 2H), 7.69 (p, *J* = 4.5 Hz, 1H), 7.56–7.48 (m, 1H), 7.45 (dd, *J* = 8.4, 6.9 Hz, 2H), 6.09 (d, *J* = 5.4 Hz, 1H), 3.40 (s, 3H). ^13^C NMR (126 MHz, CDCl_3_) δ 172.1, 161.7, 133.1, 132.6, 128.8, 127.5, 91.9, 39.6. *ν*_max_ 2921, 1643, 1551, 1491, 1380, 1151, 1013, 893 cm^−1^. ESI-HRMS: *m*/*z* = 209.0936 [M]^+^, C_10_H_10_BF_2_NO requires *m*/*z* = 209.0933. Anal. Calcd. for C_10_H_10_BF_2_NO: C, 57.47; H, 4.82; N, 6.70%. Found: C, 57.78; H, 4.67, N, 6.60%.

#### 4.5.2. 2,2-Difluoro-3,6-diphenyl-2*H*-1,3λ^4^,2λ^4^-oxazaborinine (**5b**)

Prepared from enaminone **4b** (894 mg, 4 mmol), Et_3_N (560 µL, 4 mmol) and BF_3_·Et_2_O (1.48 mL, 12 mmol), reflux for 16 h, Workup B. Yield: 797 mg (73%) of bright yellow solid, m.p. 194–195 °C. ^1^H NMR (500 MHz, CDCl_3_) δ 8.06–8.00 (m, 2H), 7.95 (s, 1H), 7.61–7.55 (m, 1H), 7.52–7.43 (m, 6H), 7.41–7.36 (m, 1H), 6.36 (d, *J* = 5.8 Hz, 1H). ^13^C NMR (126 MHz, CDCl_3_) δ 174.3, 159.6, 142.9, 133.3, 132.7, 129.7, 129.0, 128.3, 128.0, 123.5, 93.7. *ν*_max_ 1609, 1534, 1487, 1364, 1120, 1082, 1015, 998 cm^−1^. ESI-HRMS: *m*/*z* = 271.1090 [M+H]^+^, C_15_H_13_BF_2_NO requires *m*/*z* = 271.1089. Anal. Calcd. for C_15_H_12_BF_2_NO: C, 66.46; H, 4.46; N, 5.17%. Found: C, 66.48; H, 4.39, N, 5.20%. Physical and spectral data are in agreement with the literature data [20].

#### 4.5.3. 4-(2,2-Difluoro-6-phenyl-2*H*-1,3λ^4^,2λ^4^-oxazaborinin-3-yl)benzonitrile (**5c**)

Prepared from enaminone **4c** (248 g, 1 mmol), Et_3_N (140 µL, 1 mmol) and BF_3_·Et_2_O (370 μL, 3 mmol), reflux for 4 h, Workup A, CC (EtOAc–hexanes, 1:5 → EtOAc). Yield: 259 mg (88%) of yellow solid, m.p. 207–209 °C, lit. [19] m.p. 208–210 °C. ^1^H NMR (500 MHz, CDCl_3_) δ 8.08–8.02 (m, 2H), 7.98 (s, 1H), 7.79–7.72 (m, 2H), 7.67–7.58 (m, 3H), 7.53 (t, *J* = 7.8 Hz, 2H), 6.45 (d, *J* = 6.0 Hz, 1H). ^13^C NMR (126 MHz, CDCl_3_) δ 176.5, 159.4, 146.3, 134.2, 133.7, 132.2, 129.2, 128.4, 124.0, 118.2, 111.8, 94.5. *ν*_max_ 2230, 1595, 1533, 1489, 1352, 1085, 1025, 1009 cm^−1^. ESI-HRMS: *m*/*z* = 296.1045 [M]^+^, C_15_H_11_BF_2_N_2_O requires *m*/*z* = 296.1042. Anal. Calcd. for C_15_H_11_BF_2_N_2_O: C, 64.91; H, 3.74; N, 9.46%. Found: C, 64.88; H, 3.75, N, 9.16%. Physical and spectral data are in agreement with the literature data [19].

#### 4.5.4. 2,2-Difluoro-3,4,6-triphenyl-2*H*-1,3λ^4^,2λ^4^-oxazaborinine (**5d**)

Prepared from enaminone **4i** (1.50 g, 5 mmol), Et_3_N (700 µL, 5 mmol) and BF_3_·Et_2_O (1.86 mL, 15 mmol), reflux for 16 h, Workup B. Yield: 1.59 g (92%) of yellow solid, m.p. 195–196 °C, lit. [24] m.p. 195 °C. ^1^H NMR (500 MHz, CDCl_3_) δ 8.09–8.02 (m, 2H), 7.60–7.53 (m, 1H), 7.49 (m, 2H), 7.38–7.32 (m, 1H), 7.30–7.13 (m, 9H), 6.41 (s, 1H). ^13^C NMR (126 MHz, CDCl_3_) δ 172.35, 170.81, 140.66, 135.08, 133.27, 132.95, 130.62, 128.92, 128.87, 128.74, 128.61, 127.86, 127.49, 127.11, 97.00. *ν*_max_ 1595, 1566, 1506, 1486, 1231, 1107, 1020, 973 cm^−1^. ESI-HRMS: *m*/*z* = 347.1400 [M]^+^, C_21_H_16_BF_2_NO requires *m*/*z* = 347.1402. Physical and spectral data are in agreement with the literature data [24].

### 4.6. General Procedure for the Synthesis of Boron Diketonato Complexes ***6a***–***d***

These compounds were prepared according to a general procedure in the literature [21]. BF_3_·Et_2_O (1.86 mL, 15 mmol) was added to a solution of 1,3-diketone **1** (10 mmol) in anh. dichloromethane (10 mL), the mixture was stirred at room temperature for 20 h, and volatile components were evaporated in vacuo. The residue was triturated with Et_2_O (10 mL), the precipitate was collected by filtration and washed with Et_2_O (5 mL) to give compound **6**.

#### 4.6.1. 2,2-Difluoro-4,6-diphenyl-2*H*-1,3λ^3^,2λ^4^-dioxaborinine (**6a**)

Prepared from dibenzoylmethane (**1c**) (2.24 g, 10 mmol) and BF_3_·Et_2_O (1.86 mL, 15 mmol) in anh. dichloromethane (10 mL). Yield: 2.31 g (85%) of light yellow solid, m.p. 194–196 °C, lit. [49] m.p. 195–196 °C. ^1^H NMR (500 MHz, CDCl_3_) δ 8.19–8.14 (m, 2H), 7.74–7.68 (m, 2H), 7.57 (t, *J* = 7.9 Hz, 4H), 7.21 (s, 1H). Physical and spectral data are in agreement with the literature data [49].

#### 4.6.2. 2,2-Difluoro-4-methyl-6-phenyl-2*H*-1,3λ^3^,2λ^4^-dioxaborinine (**6b**)

Prepared from benzoyl acetone (**1d**) (5.00 g, 30.8 mmol) and BF_3_·Et_2_O (6.00 mL, 46.3 mmol) in anh. dichloromethane (30 mL). Yield: 5.09 g (79%) of white solid, m.p. 125–126 °C, lit. [50] m.p. 120–123 °C. ^1^H NMR (500 MHz, CDCl_3_) δ 8.08–8.05 (m, 2H), 7.71–7.67 (m, 1H), 7.56–7.51 (m, 2H), 6.58 (s, 1H), 2.42 (s, 3H). *ν*_max_ 1984, 1898, 1537, 1493, 1356, 1089, 1053, 1018, 777, 706, 679 cm^−1^. ESI-HRMS: *m*/*z* = 227.1040 [M+NH_4_]^+^, C_10_H_12_BF_2_NO_2_ requires *m*/*z* = 227.020. Physical and spectral data are in agreement with the literature data [50,51].

#### 4.6.3. 2,2-Difluoro-6-(4-methoxyphenyl)-4-methyl-2*H*-1,3λ^3^,2λ^4^-dioxaborinine (**6c**)

Prepared from (4-methoxylbenzoyl)acetone (**1e**) (1.92 g, 10 mmol) and BF_3_·Et_2_O (1.86 mL, 15 mmol) in anh. dichloromethane (10 mL). Yield: 1.69 g (70%) of yellowish resin. ^1^H NMR (500 MHz, CDCl_3_) δ 8.06 (d, *J* = 9.0 Hz, 2H), 7.00 (d, *J* = 9.0 Hz, 2H), 6.46 (s, 1H), 3.92 (s, 3H), 2.37 (s, 3H). Spectral data are in agreement with the literature data [52].

#### 4.6.4. 2,2-Difluoro-4-methyl-5,6-dihydro-2*H*-2λ^4^,3λ^3^-naphtho[1,2-*d*][1,3,2]dioxaborinine (**6d**)

Prepared from 2-acetyl-1-tetralone (**1f**) (5.00 g, 26.5 mmol) and BF_3_·Et_2_O (5.00 mL, 39.8 mmol) in anh. dichloromethane (25 mL). Yield: 5.14 g (82%) of yellow solid, m.p. 150–153 °C, lit. [28] m.p. 150–154 °C. ^1^H NMR (500 MHz, CDCl_3_) δ 8.14 (dd, *J* = 7.8, 0.9 Hz, 1H), 7.56 (td, *J* = 7.5, 1.3 Hz, 1H), 7.40 (t, *J* = 7.5 Hz, 1H), 7.28 (d, *J* = 7.3 Hz, 1H), 2.98 (t, *J* = 7.4 Hz, 2H), 2.71 (t, *J* = 7.4 Hz, 2H), 2.40 (s, 3H). *ν*_max_ 2961, 2012, 1523, 1431, 1213, 1167, 1138, 1027, 996, 794, 742, 719 cm^−1^. ESI-HRMS: *m*/*z* = 253.120 [M+NH_4_]^+^, C_12_H_14_BF_2_NO_2_ requires *m*/*z* = 253.050. Physical and spectral data are in agreement with the literature data [28].

### 4.7. General Procedure for the Synthesis of 1,5-Diketones ***7a***–***f***

A 5 mL vial with a screw cap and septum was charged with boron β-diketonate **6** (0.5 mmol), alkene (0.5–5 mmol), acetophenone (1.5 mg, 12 μmol), and dichloromethane or THF (5 mL). The vial was stopped and carefully degassed and purged with nitrogen using the freeze–pump–thaw technique. Then, the vial was mounted into a photoreactor and irradiated with UV-A light (365 nm) at 20 °C for 20 h. The reaction mixture was diluted with dichloromethane (20 mL) and washed with 1 M aq. HCl (15 mL), saturated aq. NaHCO_3_ (15 mL), and brine (15 mL). The organic phase was dried over anh. sodium sulfate, filtered, and the filtrate was evaporated in vacuo. The residue was purified by CC (ethyl acetate–petroleum ether). Fractions containing the product were combined and evaporated in vacuo to give the product **7**.

#### 4.7.1. 2-(2-Benzoylcyclohexyl)-1-phenylethan-1-one (**7a**)

Prepared from boron β-diketonate **6a** (137 mg, 0.5 mmol), cyclohexene (164 mg, 2 mmol), and acetophenone (1.5 mg, 12 μmol) in dichloromethane (5 mL), CC (EtOAc–hexanes, 1:50). Yield: 28 mg (18%) of white resin. ^1^H NMR (500 MHz, CDCl_3_) δ 8.01–7.96 (m, 4H), 7.60–7.42 (m, 6H), 3.32 (td, *J* = 11.2, 3.4 Hz, 1H), 3.13 (dd, *J* = 13.8, 2.7 Hz, 1H), 2.48 (dd, *J* = 13.8, 10.0 Hz, 1H), 2.54 (qt*, J* = 10.2, f3.2 Hz, 1H), 2.01–1.95 (m, 1H), 1.91–1.85 (m, 1H), 1.84–1.79 (m, 1H), 1.78–1.71 (m, 1H), 1.44–1.15 (m, 4H). ^13^C NMR (126 MHz, CDCl_3_) δ 203.7, 199.9, 137.3, 137.0, 133.0, 132.9, 128.8, 128.6, 128.3, 128.2, 46.6, 39.0, 33.7, 29.4, 25.9, 23.8, 23.0. *ν*_max_ 3058, 2927, 2855, 1674, 1596, 1579, 1447, 1279, 1251, 1212, 117, 1002, 981, 944, 751, 690, 663 cm^−1^. ESI-HRMS: *m*/*z* = 307.1695 [MH]^+^, C_21_H_23_O_2_ requires *m*/*z* = 307.1693. Physical and spectral data are in agreement with the literature data [53].

#### 4.7.2. 2-(2-Benzoylcyclopentyl)-1-phenylethan-1-one (**7b**)

Prepared from boron β-diketonate **6a** (68 mg, 0.25 mmol), cyclopentene (130 μL, 1.5 mmol), and acetophenone (0.75 mg, 6 μmol) in dichloromethane (2.5 mL), CC (EtOAc–hexanes, 1:50). Yield: 16 mg (22%) of white resin. ^1^H NMR (500 MHz, CDCl_3_) δ 8.00–7.93 (m, 4H), 7.58–7.51 (m, 2H), 7.47 (t, *J* = 7.7 Hz, 2H), 7.44 (t, *J* = 7.7 Hz, 2H), 3.52 (dt, *J* = 9.2, 7.8 Hz,1H), 3.19 (dd, *J* = 15.0, 5.5 Hz, 1H), 3.00 (pd, *J* = 8.3, 5.5 Hz, 1H), 2.87 (dd, *J* = 15.1, 8.5 Hz, 1H), 2.20–2.12 (m, 1H), 2.11–2.02 (m, 1H), 1.83–1.67 (m, 3H), 1.43 (dq, *J* = 12.5, 8.1 Hz, 1H). ^13^C NMR (126 MHz, CDCl_3_) δ 202.4, 199.9, 137.3, 137.0, 133.1, 133.0, 128.7, 128.7, 128.6, 128.4, 52.7, 43.9, 38.9, 32.5, 31.5, 24.8. νmax 3061, 2949, 2869, 1677, 1596, 1579, 1448, 1276, 1220, 1001, 752, 690 cm^−1^. ESI-HRMS: *m*/*z* = 293.1536 [MH]^+^, C_20_H_21_O_2_ requires *m*/*z* = 293.1536. Spectral data are in agreement with the literature data [54].

#### 4.7.3. 1-(2-Benzoylcyclohexyl)propan-2-one (**7c**)

Prepared from boron β-diketonate **6b** (106 mg, 0.5 mmol), cyclohexene (250 mg, 3 mmol), and acetophenone (1.5 mg, 12 μmol) in dichloromethane (5 mL), CC (EtOAc–hexanes, 1:50). Yield: 16 mg (13%) of white resin. ^1^H NMR (500 MHz, CDCl_3_) δ 7.91–7.86 (m, 2H), 7.53 (tt, *J* = 7.3, 1.3 Hz, 1H), 7.44 (t, *J* = 7.6 Hz, 2H), 3.63 (dt, *J* = 8.2, 4.0 Hz, 1H), 2.60–2.52 (m, 1H), 2.52 (dd, *J* = 17.1, 7.2 Hz, 1H), 2.45 (dd, *J* = 17.1, 5.5 Hz, 1H), 2.00 (s, 3H), 1.87–1.78 (m, 2H), 1.71–1.34 (m, 6H). ^13^C NMR (126 MHz, CDCl_3_) δ 208.2, 203.7, 137.0, 133.0, 128.8, 128.3, 46.3, 44.0, 32.8, 30.7, 29.7, 25.7, 23.8, 22.8. *ν*_max_ 2927, 2857, 1712, 1673, 1447, 1356, 1253, 1214, 1161, 1002, 976, 759, 699 cm^−1^. ESI-HRMS: *m*/*z* = 245.1534 [MH]^+^, C_16_H_21_O_2_ requires *m*/*z* = 245.1536. Physical and spectral data are in agreement with the literature data [53].

#### 4.7.4. 1-[2-(4-Methoxybenzoyl)cyclohexyl]propan-2-one (**7d**)

Prepared from boron β-diketonate **6c** (48 mg, 0.20 mmol), cyclohexene (120 μL, 1.2 mmol), and acetophenone (0.75 mg, 6 μmol) in THF (2.5 mL), CC (EtOAc–hexanes, 1:10). Yield: 5 mg (9%) of brown resin. ^1^H NMR (500 MHz, CDCl_3_) δ 7.95 (br d, *J* = 7.7 Hz, 2H), 6.95 (br d, *J* = 7.5 Hz, 2H), 3.87 (s, 3H), 3.18 (br t, *J* = 9.8 Hz, 1H), 2.43–2.31 (m, 2H), 2.20–2.00 (m, 1H), 2.07 (s, 3H), 1.95–1.84 (m, 2H), 1.83–1.72 (m, 2H), 1.43–1.31 (m, 3H), 1.19–1.07 (m, 1H). ^13^C NMR (126 MHz, CDCl_3_) δ 208.7, 202.3, 163.7, 130.7, 130.0, 114.0, 55.6, 49.8, 49.2, 35.3, 31.7, 31.5, 30.1, 26.1, 25.8. ν_max_ 2930, 2856, 1708, 1671, 1598, 1510, 1358, 1254, 1169, 1114, 1028, 839, 758 cm^−1^. ESI-HRMS: *m*/*z* = 275.1643 [MH]^+^, C_17_H_23_O_3_ requires *m*/*z* = 275.1642.

#### 4.7.5. 4-Acetyl-1,2,3,3a,4,5,6,11a-octahydro-11*H*-benzo[*a*]cyclopenta[*d*][8]annulen-11-one (**7e**)

Prepared from boron β-diketonate **6d** (59 mg, 0.25 mmol), cyclopentene (130 μL, 1.5 mmol), and acetophenone (1.5 mg, 12 μmol) in dichloromethane (2.5 mL), CC (EtOAc–hexanes, 1:10). Yield: 18 mg (28%) of yellowish resin. ^1^H NMR (500 MHz, CDCl_3_) δ 8.02 (dd, *J* = 7.9, 1.4 Hz, 1H), 7.46 (td, *J* = 7.4, 1.5 Hz, 1H), 7.35 (td, *J* = 7.9, 1.1 Hz, 1H), 7.22 (dd, *J* = 7.5, 0.6 Hz, 1H), 3.97 (td, *J* = 7.8, 2.4 Hz, 1H), 3.77 (td, *J* = 14.2, 5.2 Hz, 1H), 2.94 (ddd, *J* = 14.9, 5.2, 2.9 Hz, 1H), 2.48–2.29 (m, 3H), 2.04 (s, 3H), 2.02–1.86 (m, 2H), 1.82–1.70 (m, 3H), 1.61–1.50 (m, 1H), 1.31–1.18 (m, 1H). ^13^C NMR (126 MHz, CDCl_3_) δ 212.1, 204.5, 140.1, 139.4, 133.2, 132.3, 130.1, 127.2, 52.1, 51.3, 46.3, 34.4, 31.7, 31.0, 30.6, 27.8, 23.5. *ν*_max_ 2941, 2853, 1693, 1663, 1595, 1464, 1443, 1358, 1345, 1306, 1277, 1202, 1179, 759 cm^−1^. ESI-HRMS: *m*/*z* = 257.1540 [MH]^+^, C_17_H_21_O_2_ requires *m*/*z* = 257.1536.

#### 4.7.6. 5-Acetyl-1,3,4,4a,5,6,7,12a-octahydrodibenzo[*a,d*][8]annulen-12(2*H*)-one (**7f**)

Prepared from boron β-diketonate **6d** (236 mg, 1.0 mmol), cyclohexene (164 mg, 2 mmol), and acetophenone (1.5 mg, 12 μmol) in dichloromethane (5 mL), CC (EtOAc–hexanes, 1:50). Yield: 80 mg (30%) of yellowish resin. ^1^H NMR (500 MHz, CDCl_3_) δ 7.54 (br d, *J* = 7.4 Hz, 1H), 7.39 (td, *J* = 7.5, 1.4 Hz, 1H), 7.30 (td, *J* = 7.6, 0.9 Hz, 1H), 7.13 (br d, *J* = 7.6 Hz, 1H), 3.21 (dt, *J* = 15.7, 9.7 Hz, 1H), 3.15 (br s, 1H), 2.94 (dt, *J* = 15.7, 5.3 Hz, 1H), 2.57–2.42 (m, 2H), 2.11–2.00 (m, 1H), 2.06 (s, 3H), 1.97–1.88 (m, 2H), 1.85–1.71 (m, 2H), 1.60–1.50 (m, 1H), 1.48–1.32 (m, 2H), 1.29–1.21 (m 1H), 1.13–1.02 (m, 1H). ^13^C NMR (126 MHz, CDCl_3_) δ 211.6, 207.9, 140.8, 137.9, 131.3, 130.2, 128.4, 126.9, 52.1, 51.8, 38.4, 32.2, 30.5, 30.0, 27.2, 26.2, 24.4, 23.4. *ν*_max_ 2914, 1848, 1703, 1659, 1230, 954, 925, 764, 682 cm^−1^. ESI-HRMS: *m*/*z* = 271.1690 [MH]^+^, C_18_H_23_O_2_ requires *m*/*z* = 271.1693.

### 4.8. General Procedure for the Synthesis of Compounds ***8a***–***d***

A mixture of enaminone **4** (1 mmol) and anh. acetonitrile (5 mL) was stirred at 0 °C (ice bath) for 5 min. Then, Boc_2_O (327 mg, 1.5 mmol) and DMAP (12 mg, 0.1 mmol) were added, and the mixture was stirred at 0 °C for 10 min. Ice bath was removed, and the mixture was stirred at room temperature for 16 h. Volatile components were evaporated in vacuo and the residue was purified by CC (EtOAc–hexanes). Fractions containing the product were combined and evaporated in vacuo to give compound **8**.

#### 4.8.1. *tert*-Butyl (*E*)-(3-oxo-3-phenylprop-1-en-1-yl)(phenyl)carbamate (**8a**)

Prepared from enaminone **4b** (224 mg, 1 mmol), CC (EtOAc–hexanes, 1:10). Yield: 297 mg (92%) of yellow solid, m.p. 109–110 °C. ^1^H NMR (500 MHz, CDCl_3_) δ 8.60 (d, *J* = 13.7 Hz, 1H), 7.74–7.69 (m, 2H), 7.53–7.41 (m, 4H), 7.40–7.34 (m, 2H), 7.23–7.19 (m, 2H), 5.79 (d, *J* = 13.7 Hz, 1H), 1.48 (s, 9H). ^13^C NMR (126 MHz, CDCl_3_) δ 190.5, 152.0, 145.4, 138.8, 137.9, 132.2, 129.9, 128.8, 128.5, 128.2, 128.1, 105.2, 83.8, 28.1. *ν*_max_ 2983, 2969, 1720 (C=O), 1655 (C=O), 1564, 1345, 1270, 1141 cm^−1^. ESI-HRMS: *m*/*z* = 324.1589 [M+H]^+^, C_20_H_22_NO_3_ requires *m*/*z* = 324.1594.

#### 4.8.2. *tert*-Butyl (*E*)-allyl(3-(4-chlorophenyl)-3-oxoprop-1-en-1-yl)carbamate (**8b**)

Prepared from enaminone **4e** (444 mg, 2 mmol), CC (EtOAc–hexanes, 1:5). Yield: 644 mg (100%) of yellow solid, m.p. 83–84 °C. ^1^H NMR (500 MHz, CDCl_3_) δ 8.40 (d, *J* = 13.7 Hz, 1H), 7.82 (d, *J* = 8.6 Hz, 2H), 7.42 (d, *J* = 8.5 Hz, 2H), 6.20 (d, *J* = 13.8 Hz, 1H), 5.81 (ddt, *J* = 17.2, 10.3, 5.1 Hz, 1H), 5.25 (dq, *J* = 10.5, 1.4 Hz, 1H), 5.19 (dq, *J* = 17.1, 1.7 Hz, 1H), 4.29 (dt, *J* = 5.3, 1.8 Hz, 2H), 1.54 (s, 9H). ^13^C NMR (126 MHz, CDCl_3_) δ 189.0, 152.1, 144.2, 138.58, 137.4, 131.3, 129.6, 128.9, 117.2, 102.7, 83.9, 47.2, 28.2. *ν*_max_ 3118, 3010, 2928, 1715 (C=O), 1653 (C=O), 1590, 1577, 1560, 1489, 1456, 1409, 1393, 1368, 1359, 1320, 1283, 1244, 1204, 1172, 1135, 1090, 1045, 1011, 981, 956, 944, 933, 912, 886, 845, 832, 816, 771, 742, 680 635 cm^−1^. ESI-HRMS: *m*/*z* = 322.1203 [M+H]^+^, C_17_H_21_ClNO_3_ requires *m*/*z* = 322.1204.

#### 4.8.3. *tert*-Butyl (*E*)-(3-oxo-3-phenylprop-1-en-1-yl)(pyridin-2-yl)carbamate (**8c**)

Prepared from enaminone **4f** (224 mg, 1 mmol), CC (EtOAc–hexanes, 1:5). Yield: 324 mg (100%) of yellow solid, m.p. 105–107 °C. ^1^H NMR (500 MHz, CDCl_3_) δ 8.68 (ddd, *J* = 4.8, 2.0, 0.8 Hz, 1H), 8.50 (d, *J* = 14.0 Hz, 1H), 7.90 (td, *J* = 7.7, 2.0 Hz, 1H), 7.76–7.71 (m, 2H), 7.51–7.45 (m, 1H), 7.43–7.36 (m, 3H), 7.28 (dt, *J* = 7.8, 1.0 Hz, 1H), 5.81 (d, *J* = 13.9 Hz, 1H), 1.49 (s, 9H). ^13^C NMR (126 MHz, CDCl_3_) δ 190.5, 151.5, 151.2, 150.1, 144.1, 139.1, 138.7, 132.2, 128.5, 128.1, 124.0, 123.4, 105.5, 84.2, 28.1. *ν*_max_ 3012, 2979, 1720 (C=O), 1655 (C=O), 1563, 1467, 1213, 1098 cm^−1^. ESI-HRMS: *m*/*z* = 325.1540 [M+H]^+^, C_19_H_21_N_2_O_3_ requires *m*/*z* = 325.1547.

#### 4.8.4. *tert*-Butyl (*E/Z*)-(3-oxo-1,3-diphenylprop-1-en-1-yl)(phenyl)carbamate (**8d**)

Prepared from enaminone **4i** (299 mg, 1 mmol), CC (EtOAc–hexanes, 1:5). Yield: 352 mg (88%) of light yellow solid, m.p. 113–124 °C, *E/Z* = 4:1. ^1^H NMR (500 MHz, CDCl_3_) δ major isomer 7.88 (d, *J* = 7.5 Hz, 2H), 7.69–7.60 (m, 2H), 7.60–7.50 (m, 1H), 7.48–7.35 (m, 5H), 7.28–7.21 (m, 1H), 7.19–7.10 (m, 3H), 7.06–6.98 (m, 1H), 6.91 (s, 1H), 1.26 (s, 9H); minor isomer 7.76 (dd, *J* = 8.3, 1.2 Hz, 2H), 7.47–7.37 (m, 7H), 7.30 (t, *J* = 7.7 Hz, 2H), 7.27–7.21 (m, 1H), 7.20–7.10 (m, 3H), 6.37 (s, 1H), 1.20 (s, 9H). ^13^C NMR (126 MHz, CDCl_3_) δ major isomer ^13^C NMR (126 MHz, CDCl_3_) δ 189.8, 153.1, 151.4, 141.7, 138.4, 132.9, 130.1, 129.4, 128.9, 128.6, 128.5, 128.4, 127.4, 127.0, 125.3, 119.0, 81.9, 28.0; minor isomer 192.8, 154.4, 153.5, 143.0, 138.2, 137.1, 132.7, 130.1, 129.5, 128.9, 128.8, 128.3, 128.2, 126.7, 125.5, 121.4, 82.4, 27.8. *ν*_max_ 2977, 1713 (C=O), 1660 (C=O), 1595, 1491, 1448, 1367, 1339, 1269, 1246, 1153, 1014, 847, 771, 741, 692 cm^−1^. Anal. Calcd. for C_26_H_25_NO_3_·⅙H_2_O: C, 77.52; H, 6.69, N, 3.45%. Found: C, 77.59; H, 6.34, N, 3.48%.

### 4.9. Synthesis of (Z)-{2-Benzoyl-3-[(4-cyanophenyl)amino]acryloyl}sulfamoyl chloride (***9***)

Under argon, chlorosulfonyl isocyanate (CSI) (130 μL, 1.5 mmol) was added to a suspension of enaminone **4c** (248 mg, 1 mmol) in anhydrous Et_2_O (10 mL) at 0 °C (ice-bath) and the mixture was stirred at 0 °C for 15 minutes. Then, the ice bath was removed and stirring was continued at room temperature (~20 °C) for 1 h. The precipitate was collected by filtration, washed with anh. Et_2_O (3 × 5 mL) and dried in vacuo over NaOH pellets for 12 h. Yield: 310 mg (80%) of yellowish solid, m.p. 150 °C (decomp.). ^1^H NMR (600 MHz, CDCl_3_) δ 13.84 (s, 1H), 12.14 (d, *J* = 13.4 Hz, 1H), 8.26 (d, *J* = 13.4 Hz, 1H), 7.69 (br d, *J* = 8.7 Hz, 2H), 7.67–7.62 (m,1H), 7.59–7.54 (m, 4H), 7.13 (br d, *J* = 8.7 Hz, 2H). ^13^C NMR (150 MHz, CDCl_3_) δ 196.1, 165.7, 156.4, 141.2, 137.6, 134.3, 132.5, 129.0, 128.7, 118.2, 117.8, 110.2, 102.6. *ν*_max_ 2223 (C≡N), 1775 (C=O), 1717 (C=O), 1672 (C=O), 1629 (C=O), 1588, 1567, 1440, 1414, 1318, 1275, 1177, 907, 841, 814, 762, 734, 709, 661 cm^−1^.

### 4.10. Allylation of Enaminone 4b. Synthesis of Compounds ***10***–***12***

Under argon, allyl bromide (175 μL, 2 mmol) was added to a stirred mixture of enaminone **4b** (224 mg, 1 mmol), K_2_CO_3_ (500 mg, 3.6 mmol), NaI (12 mg, 0.08 mmol), and anh. acetonitrile (25 mL). The reaction mixture was then stirred under reflux under argon for 16 h. Volatile components were evaporated in vacuo to give a crude mixture of compounds **10**, **11**, and **12**. The residue was purified by CC (EtOAc–hexanes, 1:5). Compound **10** eluted first, followed by elution of compound **12** and compound **11**. Fractions containing the products **10**, **11**, and **12** were combined and evaporated in vacuo to give compounds **10**, **11**, and **12**.

#### 4.10.1. (*E*)-2-Allyl-1-phenyl-2-[(phenylimino)methyl]pent-4-en-1-one (**10**)

Yield: 135 mg (44%) of yellow–orange oil. ^1^H NMR (500 MHz, CDCl_3_) δ 8.00 (s, 1H), 7.93–7.89 (m, 2H), 7.53–7.49 (m, 1H), 7.44–7.39 (m, 2H), 7.37–7.32 (m, 2H), 7.24–7.19 (m, 1H), 7.04–6.99 (m, 2H), 5.74 (ddt, *J* = 17.5, 10.2, 7.4 Hz, 2H), 5.12–5.02 (m, 4H), 2.94 (dt, *J* = 7.4, 1.2 Hz, 4H). ^13^C NMR (126 MHz, CDCl_3_) δ 200.0, 166.7, 151.7, 137.2, 132.6, 132.4, 129.2, 129.2, 128.4, 126.0, 120.4, 119.2, 59.9, 38.3. ESI-HRMS: *m*/*z* = 304.1687 [M+H]^+^, C_21_H_22_NO requires *m*/*z* = 304.1696.

#### 4.10.2. (*E*)-3-[Allyl(phenyl)amino]-1-phenylprop-2-en-1-one (**11**)

Yield: 106 mg (40%) of yellow–orange oil. ^1^H NMR (500 MHz, CDCl_3_) δ 8.24 (d, *J* = 12.8 Hz, 1H), 7.90 (dt, *J* = 7.1, 1.4 Hz, 2H), 7.51–7.46 (m, 1H), 7.46–7.41 (m, 2H), 7.40–7.35 (m, 2H), 7.26–7.23 (m, 2H), 7.19 (td, *J* = 7.3, 1.1 Hz, 1H), 6.10 (d, *J* = 12.9 Hz, 1H), 5.94 (ddt, *J* = 17.2, 10.5, 4.8 Hz, 1H), 5.34–5.26 (m, 2H), 4.39 (dt, *J* = 4.2, 1.9 Hz, 2H). ^13^C NMR (126 MHz, CDCl_3_) δ 189.6, 149.2, 140.1, 131.5, 131.1, 129.7, 129.3, 128.4, 127.8, 125.3, 117.8, 115.6, 97.7, 53.5. ESI-HRMS: *m*/*z* = 264.1375 [M+H]^+^, C_18_H_18_NO requires *m*/*z* = 264.1383.

#### 4.10.3. (*E*)-2-{[Allyl(phenyl)amino]methylene}-1-phenylpent-4-en-1-one (**12**)

Yield: 21 mg (7%) of yellow–orange oil. ^1^H NMR (500 MHz, CDCl_3_) δ 7.57–7.54 (m, 2H), 7.42–7.38 (m, 3H), 7.35 (s, 1H), 7.31–7.26 (m, 2H), 7.14–7.09 (m, 1H), 7.06–7.02 (m, 2H), 5.96–5.79 (m, 2H), 5.21 (d, *J* = 2275.4 Hz, 2H), 4.95 (dd, *J* = 99.1, 53.9 Hz, 2H), 4.31 (dt, *J* = 4.6, 1.9 Hz, 2H), 3.12 (dt, *J* = 5.4, 1.9 Hz, 2H). ^13^C NMR (126 MHz, CDCl_3_) δ 197.4, 151.5, 146.0, 141.2, 137.1, 133.5, 129.9, 129.2, 128.6, 128.0, 125.0, 121.9, 117.2, 114.3, 114.3, 55.8, 28.9. ESI-HRMS: *m*/*z* = 304.1691 [M+H]^+^, C_21_H_22_NO requires *m*/*z* = 304.1696.

### 4.11. Synthesis of 1-allyl-3,5-bis(4-chlorobenzoyl)-1,4-dihydropyridine (***13***)

A 5 mL flask was charged with compound **4e** (222 mg, 1 mmol) and the flask was cooled to 0 °C (ice bath). Formic acid (700 μL), 37% aq. formaldehyde (1.35 mL), and water (1 mL) were added at 0 °C (ice bath), the flask was equipped with a reflux condenser, and the mixture was stirred at 90 °C for 48 h. The reaction mixture was cooled to room temperature, diluted with 6 M hydrochloric acid (20 mL), and the product was extracted with dichloromethane (3 × 15 mL). The combined organic phase was evaporated in vacuo and the residue was purified by CC (CH_2_Cl_2_–MeOH, 50:1). Fractions containing the product were combined and evaporated in vacuo to give **13**. Yield: 42 mg (22%) of brownish solid, m.p. 156–159 °C. ^1^H NMR (500 MHz, CDCl_3_) δ 7.49 (d, *J* = 8.4 Hz, 4H), 7.40 (d, *J* = 8.4 Hz, 4H), 6.65 (s, 2H), 5.79 (ddt, *J* = 17.0, 10.5, 5.3 Hz, 1H), 5.30 (dd, *J* = 8.6, 1.8 Hz, 1H), 5.25 (dd, *J* = 17.2, 1.0 Hz, 1H), 3.82 (dt, *J* = 5.5, 1.6 Hz, 2H), 3.51 (s, 2H). ^13^C NMR (150 MHz, CDCl_3_) δ 193.0, 143.1, 137.4, 137.1, 132.1, 129.9, 128.6, 119.2, 115.7, 56.8, 21.4. *ν*_max_ 3088, 2827, 2050, 1664, 1610, 1584, 1572, 1483, 1451, 1396, 1374, 1289, 1258, 1201, 1170, 1136, 1087, 1012, 976, 961, 932, 911, 845, 826, 779, 743, 683, 654, 628 cm^−1^. ESI-HRMS: *m*/*z* = 398.0691 [M+H]^+^, C_22_H_28_Cl_2_NO_2_ requires *m*/*z* = 398.0709.

## 5. Conclusions

An array of acetophenone-derived enaminones were prepared via the transamination of *N,N*-dimethylenaminones. The corresponding BF_2_-β-ketoiminates as well as BF_2_-β-diketonates were then prepared and tested in De Mayo reactions with cyclic alkenes. While BF_2_-β-diketonates gave the desired products in low yields, BF_2_-β-ketoiminates remained unconverted. The photochemical transformation of tricyclic BF_2_-acetyltetralone-complex resulted in ring expansion to give novel diannulated cyclooctane derivatives, which would be difficult to obtain using conventional cyclization methods. Additionally, *N*-Boc, α-chlorosulfonylated, *C,C*-diallylated, *N*-monoallyated, and *C,N*-diallylated derivatives of enaminones were prepared. While their attempted photochemical transformations failed, interesting mechanistic insights were made into allylation of enaminones as well as their conversion to 1,4-dihydropyridine derivative.

## Figures and Tables

**Table 1:** Selected experimental data for amines **3a**–**g** and enaminones **2a,b** and **4a**–**i**.

Transformation	Ar	R^1^	R^2^	Method	*E* or *Z*	Yield (%)
**1a** → **2a**	Ph	Me	Me	-	*E*	85
**1b** → **2b**	4-ClC_6_H_4_	Me	Me	-	*E*	62
**2a**+**3a** → **4a**	Ph	Me	H	A	*Z*	57
**2a**+**3b** → **4b**	Ph	Ph	H	A	*Z*	95
**2a**+**3c** → **4c**	Ph	4-NCC_6_H_4_	H	A	*Z*	84
**2a**+**3d** → **4d**	Ph	4-MeOC_6_H_4_	H	A	*Z*	18
**2b**+**3e** → **4e**	4-ClC_6_H_4_	allyl	H	A	*Z*	69
**2b**+**3e** → **4e**	4-ClC_6_H_4_	allyl	H	B	*Z*	81
**2a**+**3f** → **4f**	Ph	2-pyridyl	H	B	*Z*	63
**2a**+**3g** → **4g**	Ph	Ph	Me	B	*E*	71
**2b**+**3g** → **4h**	4-ClC_6_H_4_	Ph	Me	B	*E*	45
**1c**+**3b** → **4i**	-	-	-	C	*Z*	77

**Table 2:** Selected experimental data for compounds **5a**–**d**, **6a**–**d**, and **7a**–**f**.

Transformation	Ar	R^1^	R^2^	n	Yield (%)
**4a** **→** **5a**	-	H	Me	-	78
**4b** **→** **5b**	-	H	Ph	-	73
**4c** **→** **5c**	-	H	4-NCC_6_H_4_	-	88
**4i** **→** **5d**	-	Ph	Ph	-	92
**1c** **→** **6a**	Ph	Ph	-	-	85
**1d** **→** **6b**	Ph	Me	-	-	79
**1d** **→** **6c**	4-MeOC_6_H_4_	Me	-	-	70
**1f** **→** **6d**	-	-	-	-	82
**6a** **→** **7a**	Ph	Ph	-	2	18
**6a** **→** **7b**	Ph	Ph	-	1	22
**6b** **→** **7c**	Ph	Me	-	2	13
**6c** **→** **7d**	4-MeOC_6_H_4_	Me	-	2	9
**6d** **→** **7e**	-	-	-	1	28
**6d** **→** **7f**	-	-	-	2	30

**Table 3:** Selected experimental data for compounds **8a**–**d** and **9**–**13**.

Transformation	Ar	R^1^	R^2^	Solvent	Yield (%)
**4b** **→** **8a**	Ph	Ph	H	MeCN	92
**4e** **→** **8b**	4-ClC_6_H_4_	allyl	H	MeCN	100
**4f** **→** **8c**	Ph	2-pyridyl	H	MeCN	100
**4i** **→** **8d**	Ph	Ph	Ph	MeCN	88
**4c** **→** **9**	4-NCC_6_H_4_	-	-	MeCN	65
**4c** **→** **9**	4-NCC_6_H_4_	-	-	Et_2_O	80
**4c** **→** **9**	4-NCC_6_H_4_	-	-	toluene	81
**4b** **→** **10**	-	-	-	MeCN	44
**4b** **→** **11**	-	-	-	MeCN	40
**4b** **→** **12**	-	-	-	MeCN	7
**4e** **→** **13**	4-ClC_6_H_4_	-	-	H_2_O-HCO_2_H	22

## Data Availability

The data presented in this study are available in the main manuscript and in the Supplementary Materials of this manuscript.

## Supplementary Materials

The following supporting information can be downloaded at: https://www.mdpi.com/article/10.3390/molecules30030601/s1, copies of ^1^H NMR and ^13^C NMR of compounds **2** and **4**–**13**; Figure S1: photochemical reaction setup. Figures S2–S5: X-Ray structures of compounds **8a**, **8c**, **8d**, and **5b**; Figure S6: absorption spectra of enaminones **4b**–**d,f,g,i**, β-ketoiminate complexes **5a**–**d**, *N*-Boc-enaminones **8a,c,d**, and compound **9**. Table S1. Crystal data and structure refinement for **5b**, **8a**, **8c**, and **8d**. References [55,56,57,58,59,60,61] are cited in the supplementary materials.
