# Supplementary material for: Preparation and Transformations of Acetophenone-Derived Enamino Ketones, BF2-β-Ketoiminates, and BF2-β-Diketonates
Source: Molecules. 2025 Jan 29;30(3):601. doi: 10.3390/molecules30030601 (PMC11820750; doi:10.3390/molecules30030601)
Supplement: Supplementary file 1 [file molecules-30-00601-s001.zip › molecules-3402544-supplementary.pdf]

molecules

Supporting information for the article

Preparation and Transformations of Acetophenone-Derived Enamino Ketones, BF_2-β-Ketoiminates, and BF_2-β-Diketonates

Helena Brodnik, Luka Ciber, Uroš Grošelj, Nejc Petek *, Bogdan Štefane and Jurij Svete *

Faculty of Chemistry and Chemical Technology, University of Ljubljana, Večna pot 113, 1000 Ljubljana, Slovenia; helena.brodnik@fkkt.uni-lj.si (H.B.); luka.ciber@fkkt.uni-lj.si (L.C.); uros.groselj@fkkt.uni-lj.si (U.G.); bogdan.stefane@fkkt.uni-lj.si (B.Š.)

* Correspondence: nejc.petek@fkkt.uni-lj.si (N.P.); jurij.svete@fkkt.uni-lj.si (J.S.); Tel.: +386-1-479-8562 (J.S.)

Table of Contents

1. Experimental details (reaction setup). S2
2. Copies of NMR spectra of compounds **2** and **4–13**. S3
3. X-Ray structure determination for compounds **5b**, **8a**, **8c**, and **8d**. S34
4. Absorption spectra of compounds **4**, **5**, **8**, and **9**. S37
5. References. S38

 https://doi.org/10.3390/molecules30030601

1. Experimental details (reaction setup).

Photochemical transformations were performed on benchtop Penn PhD Photoreactor M2 with LED light source and air-cooling (Penn Photon Devices, Pennsburg, PA, USA). LEDs used: 365 nm (3 W). Reactions were performed in 8 mL glass vials using an 8 mL vial holder. More details on Photoreactor M2 can be found at https://www.sigmaaldrich.com/deepweb/assets/sigmaaldrich/product/documents/212/357/photoreactor-m2-user-manual.pdf?srsltid=AfmBOoptoY_QgnN6KCi-N-Pb58phMs2baTRtzQ9zJ6IekNZFAoHoeZN9. The photochemical reaction setup is shown in Figure S1.

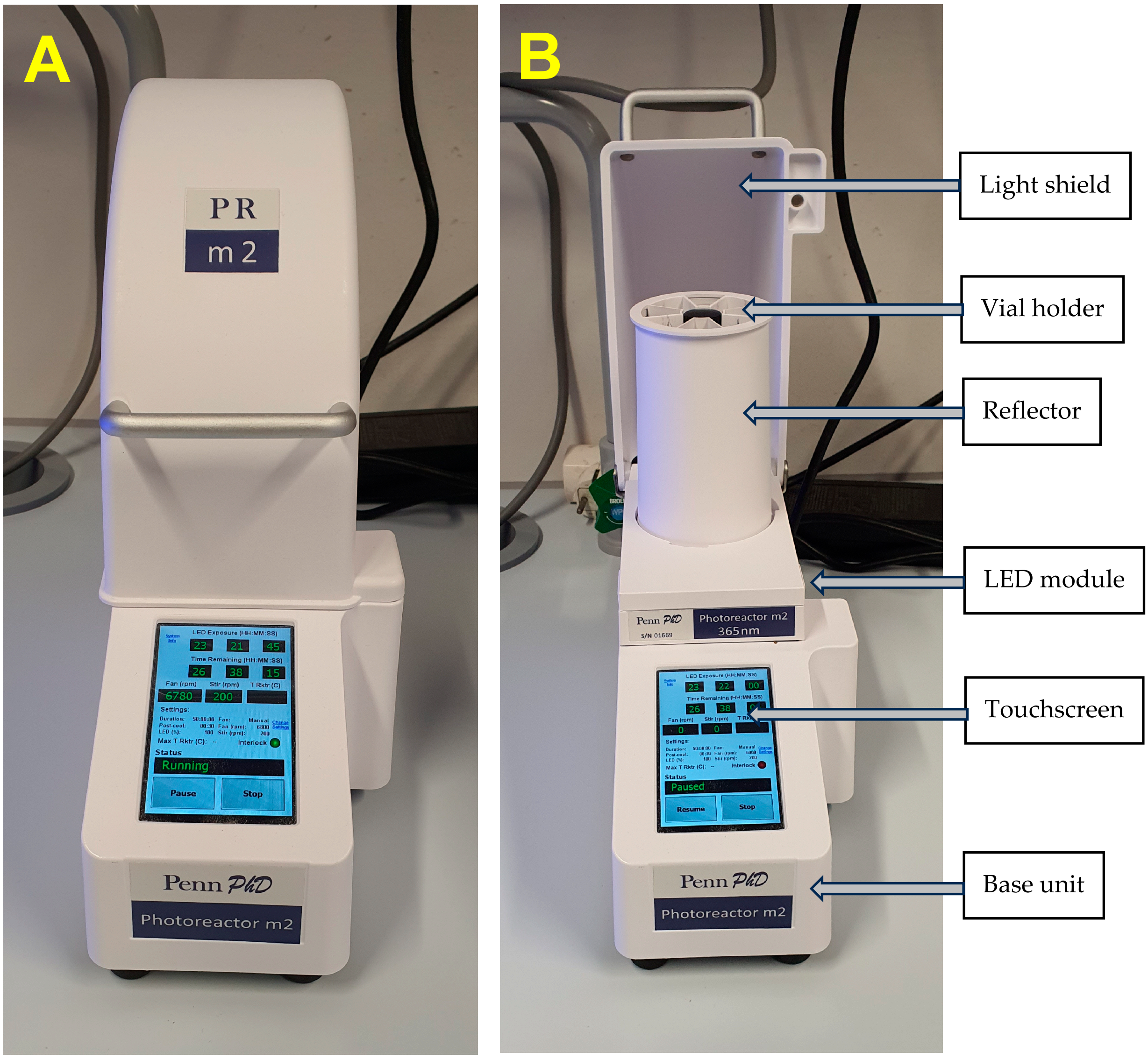

Figure S1. Photochemical reaction setup with (**A**) closed an (**B**) opened light shield with marked components of photoreactor.

2. Copies of NMR spectra of compounds 2 and 4–13.

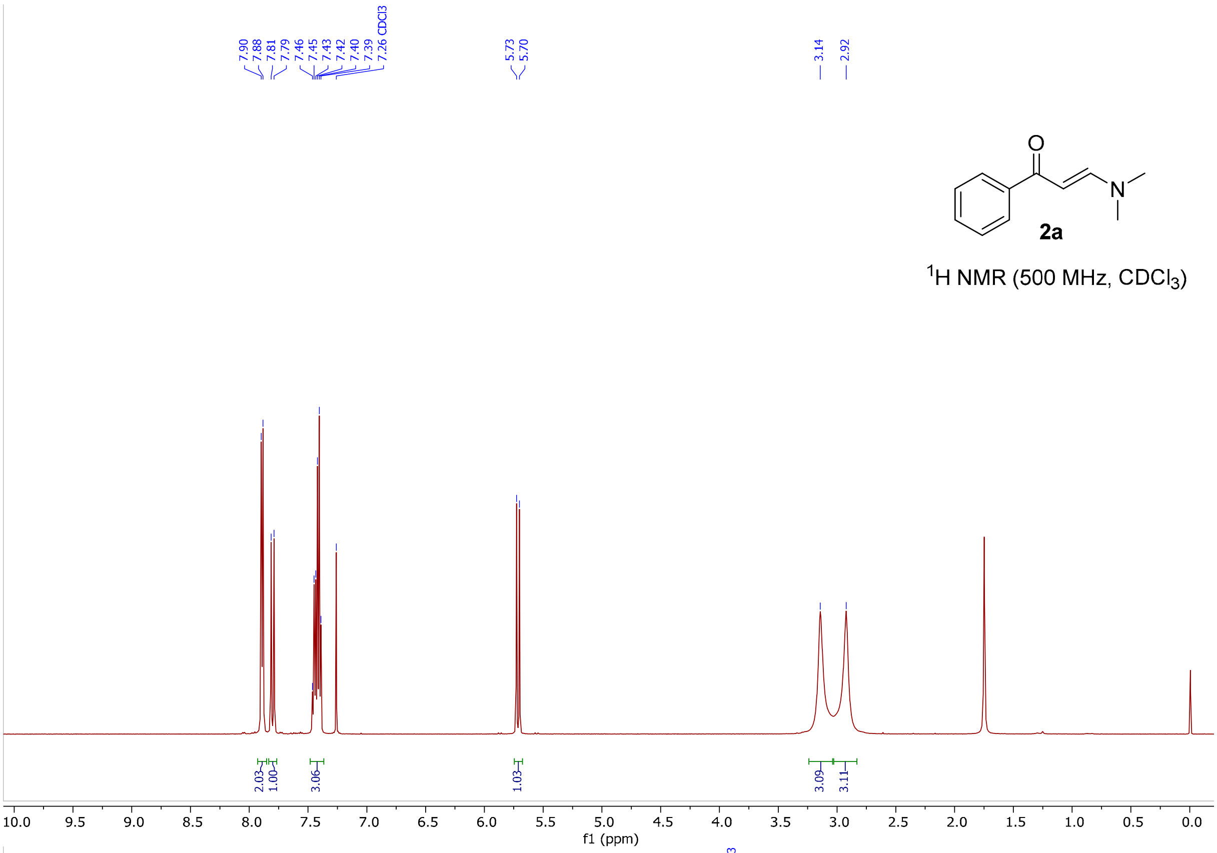

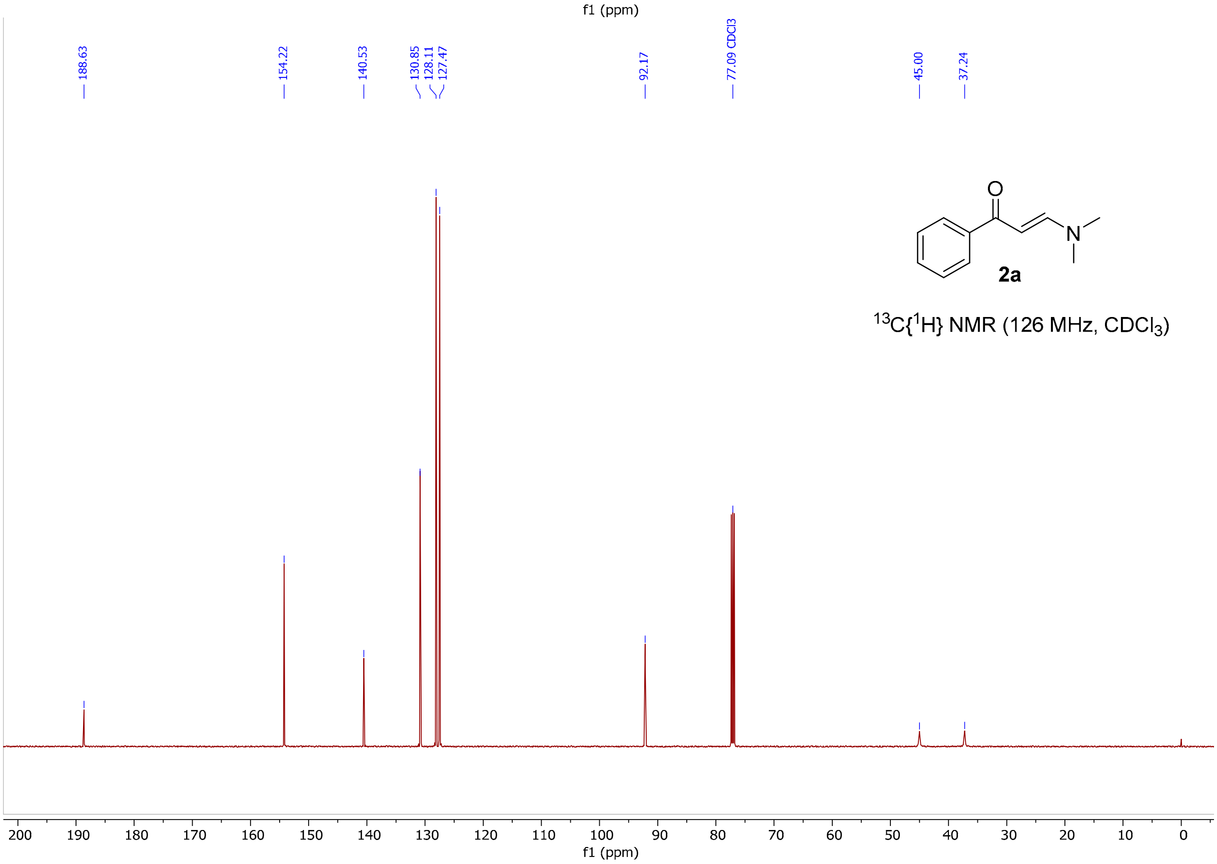

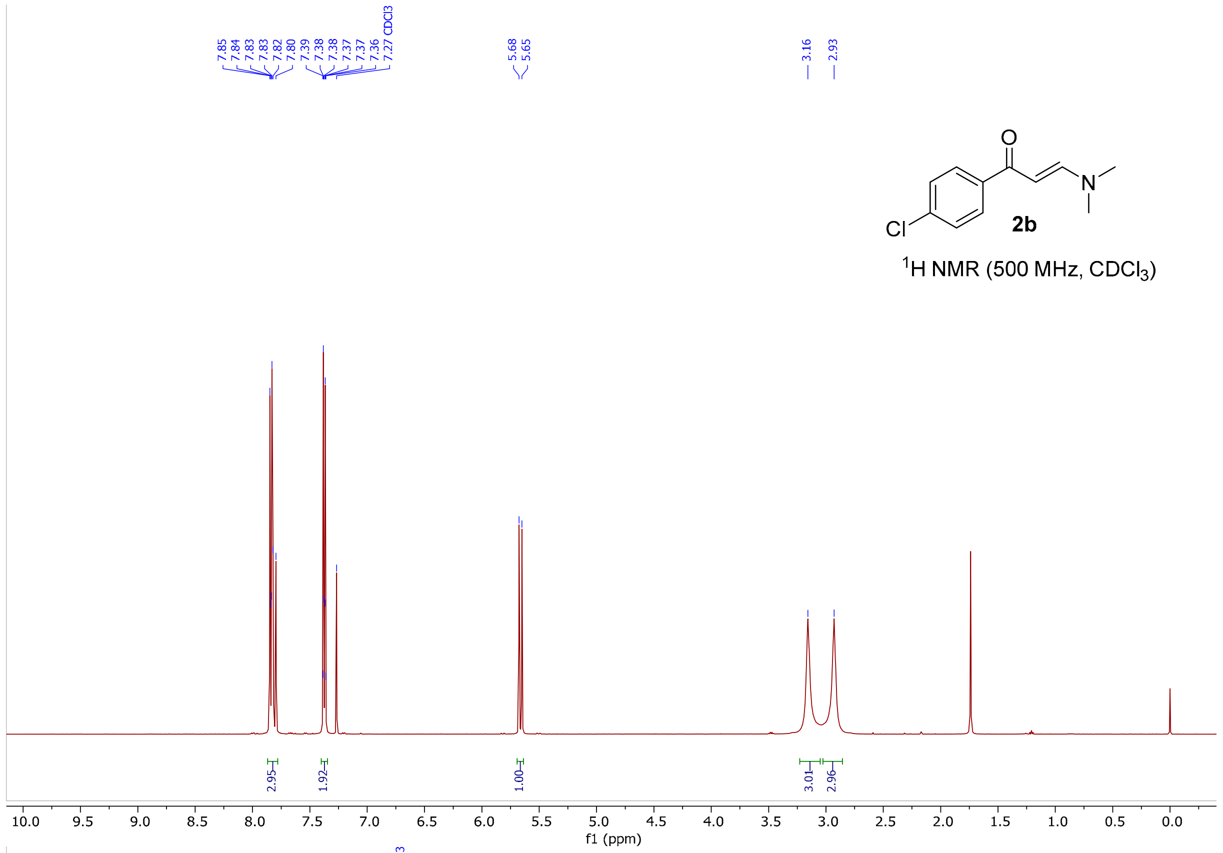
7.85
7.84
7.83
7.83
7.82
7.80
7.39
7.38
7.38
7.37
7.37
7.36
7.27 CDCl3
5.68
5.65
3.16
2.93
O
N
Cl
2b
1H NMR (500 MHz, CDCl3)
2.95
1.92
1.00
3.01
2.96
10.0 9.5 9.0 8.5 8.0 7.5 7.0 6.5 6.0 5.5 5.0 4.5 4.0 3.5 3.0 2.5 2.0 1.5 1.0 0.5 0.0
f1 (ppm)

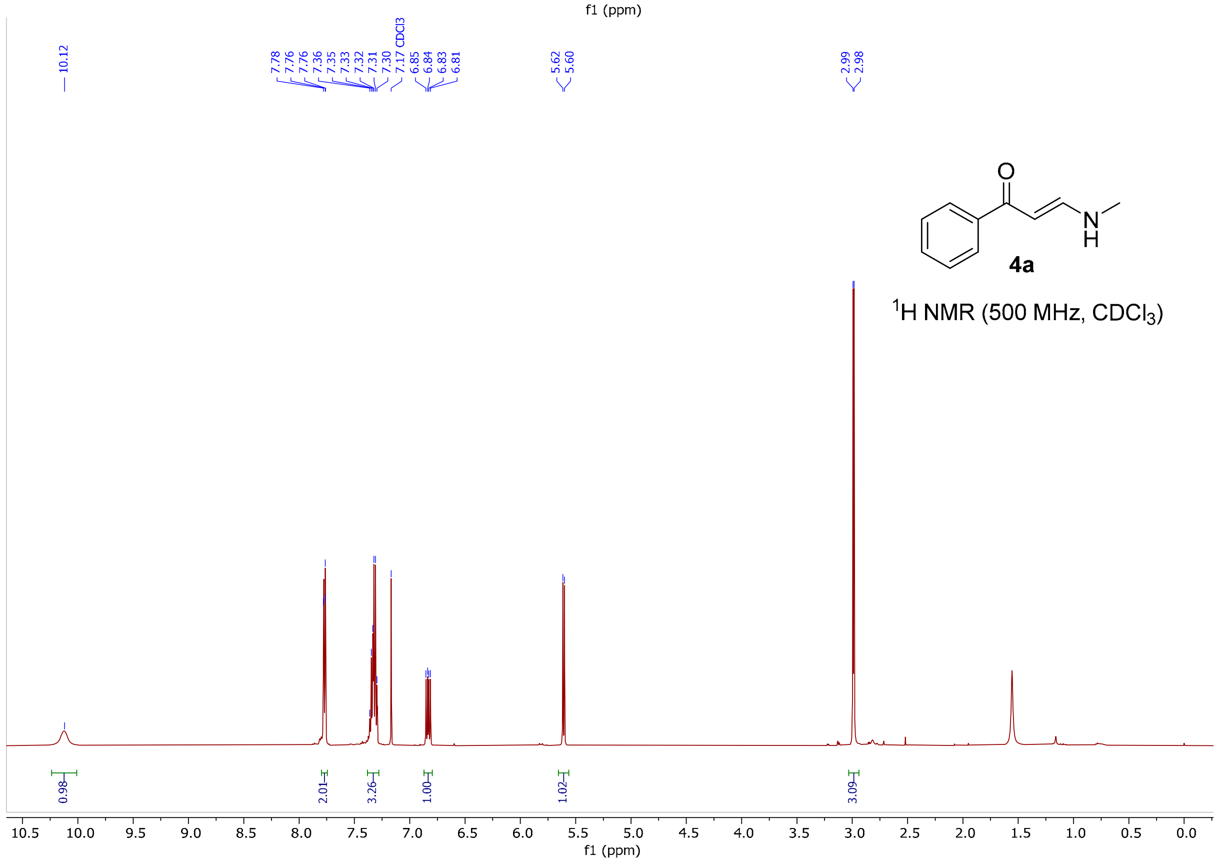
10.12
7.78
7.76
7.76
7.36
7.35
7.33
7.32
7.31
7.30
7.17 CDCl3
6.85
6.84
6.83
6.81
5.62
5.60
2.99
2.98
O
N
H
4a
1H NMR (500 MHz, CDCl3)
0.98
2.01
3.26
1.00
1.02
3.09
10.5 10.0 9.5 9.0 8.5 8.0 7.5 7.0 6.5 6.0 5.5 5.0 4.5 4.0 3.5 3.0 2.5 2.0 1.5 1.0 0.5 0.0
f1 (ppm)

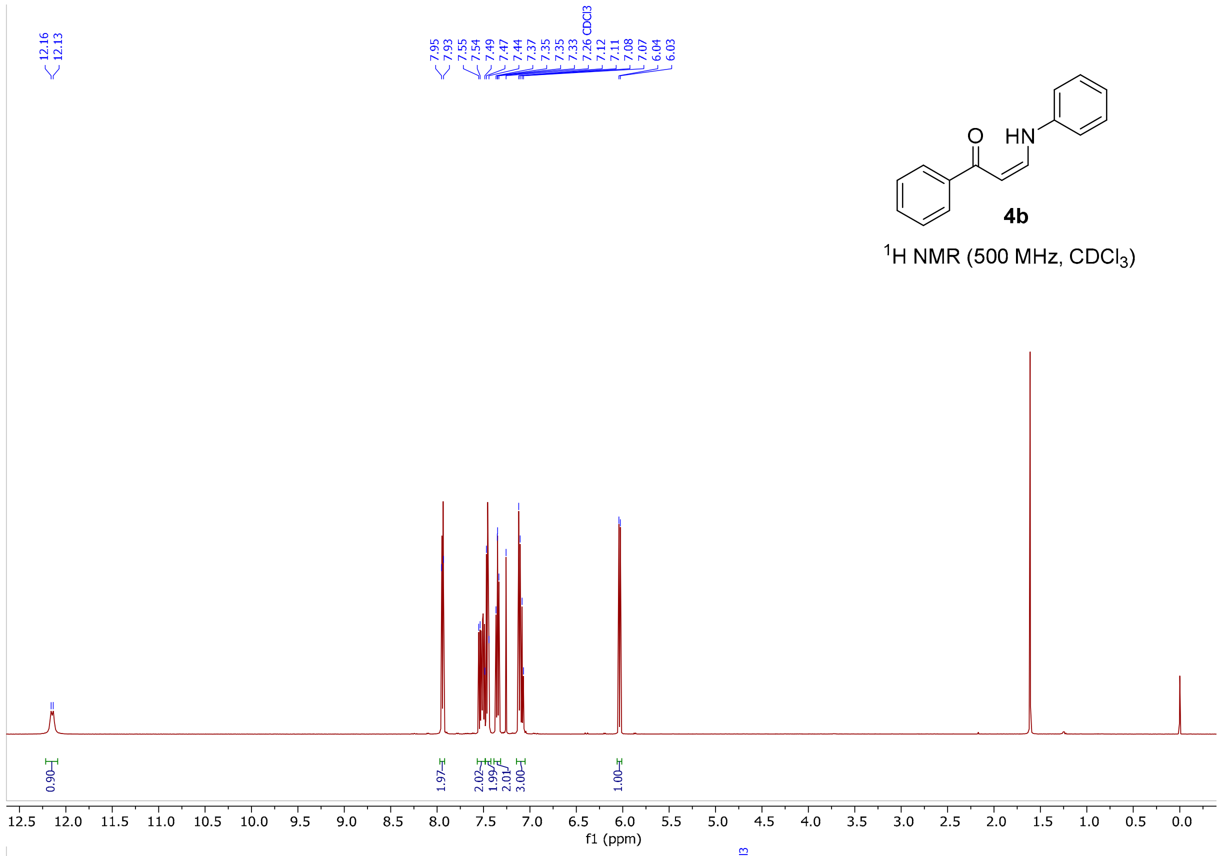
12.16
12.13
7.95
7.93
7.55
7.54
7.49
7.47
7.44
7.37
7.35
7.35
7.33
7.26 CDCl3
7.12
7.11
7.08
7.07
6.04
6.03
O
HN
4b
1H NMR (500 MHz, CDCl3)
0.90
1.97
2.02
1.99
2.01
3.00
1.00
f1 (ppm)

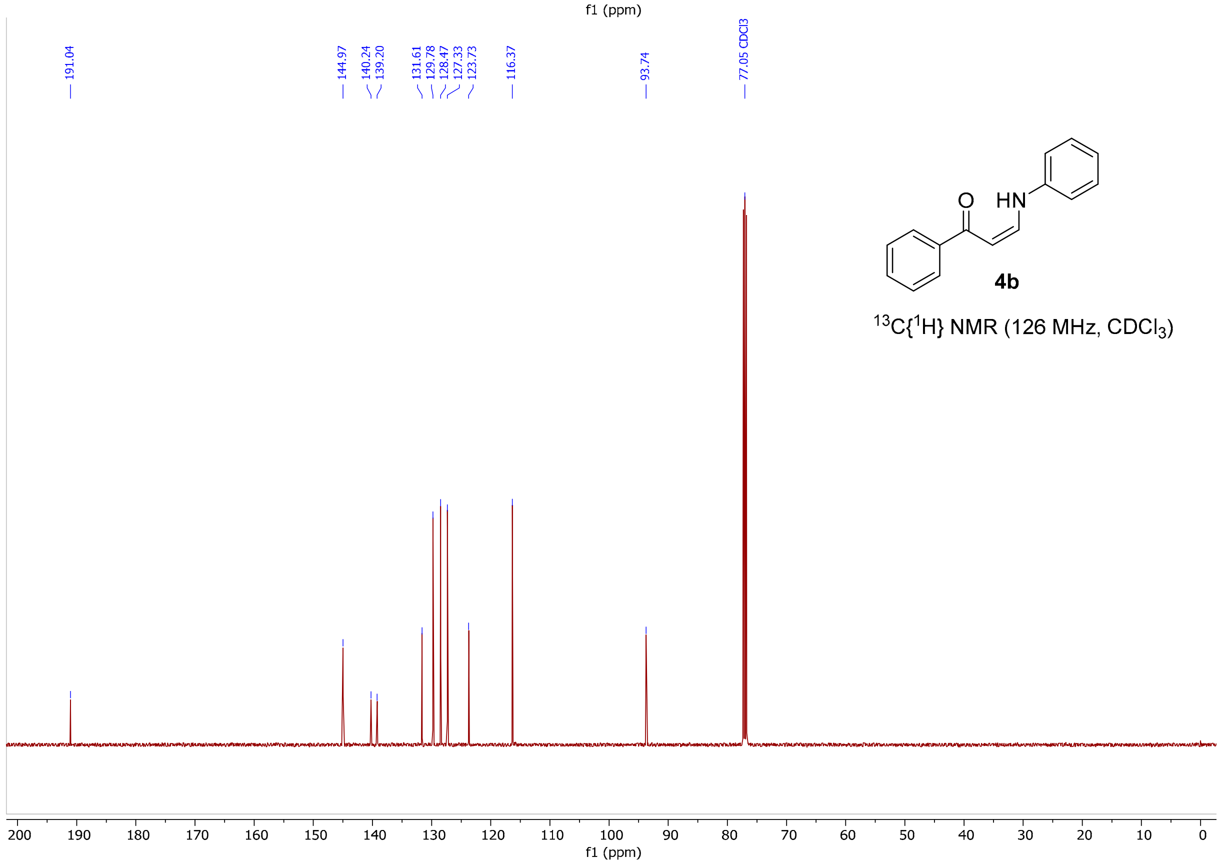
191.04
144.97
140.24
139.20
131.61
129.78
128.47
127.33
123.73
116.37
93.74
77.05 CDCl3
O
HN
4b
13C{1H} NMR (126 MHz, CDCl3)
f1 (ppm)

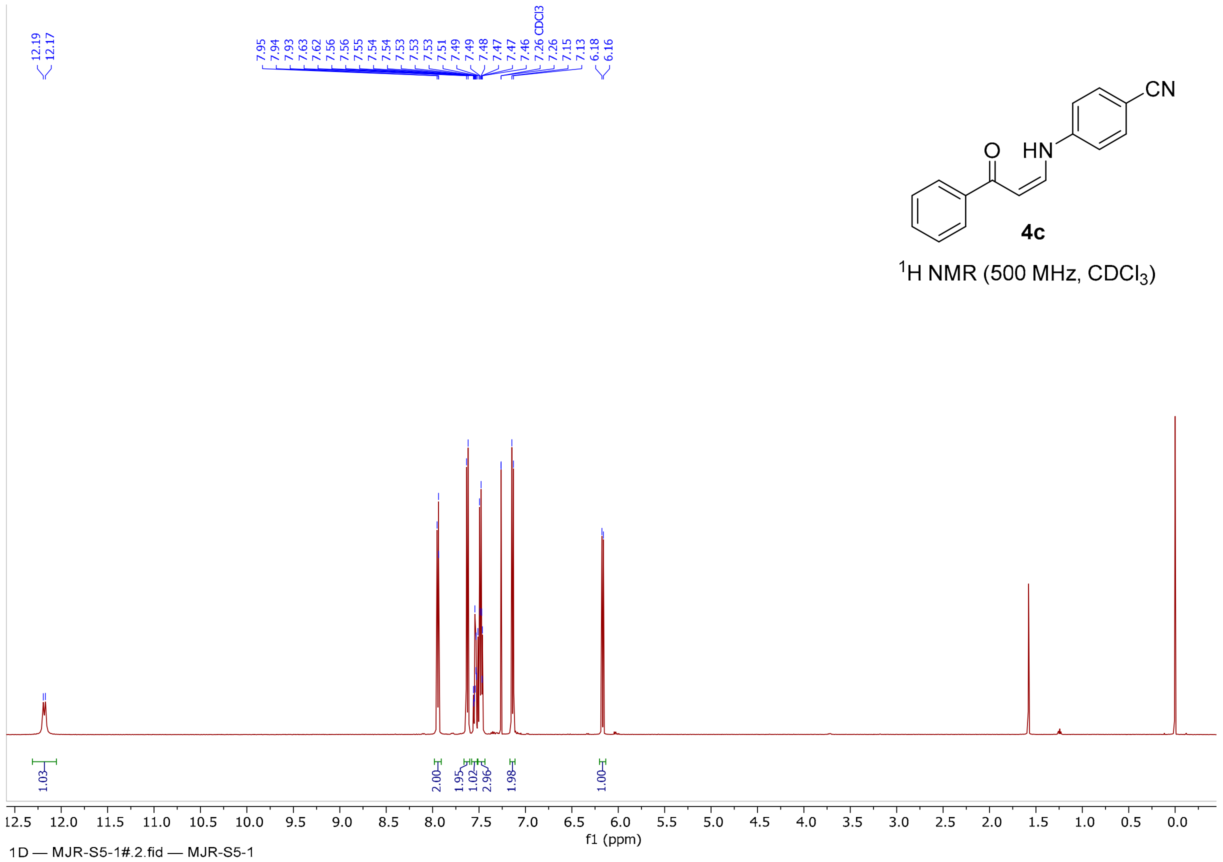

1D — MJR-S5-1#.2.fid — MJR-S5-1

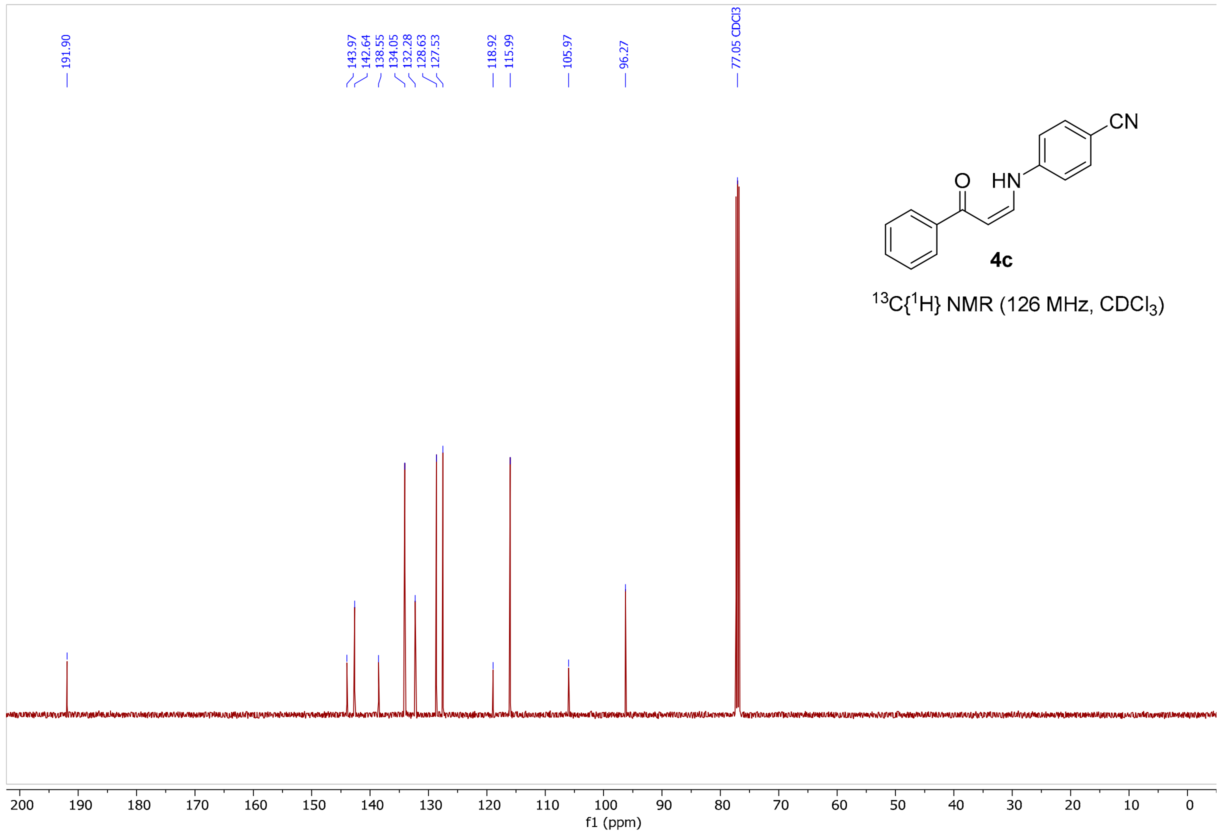

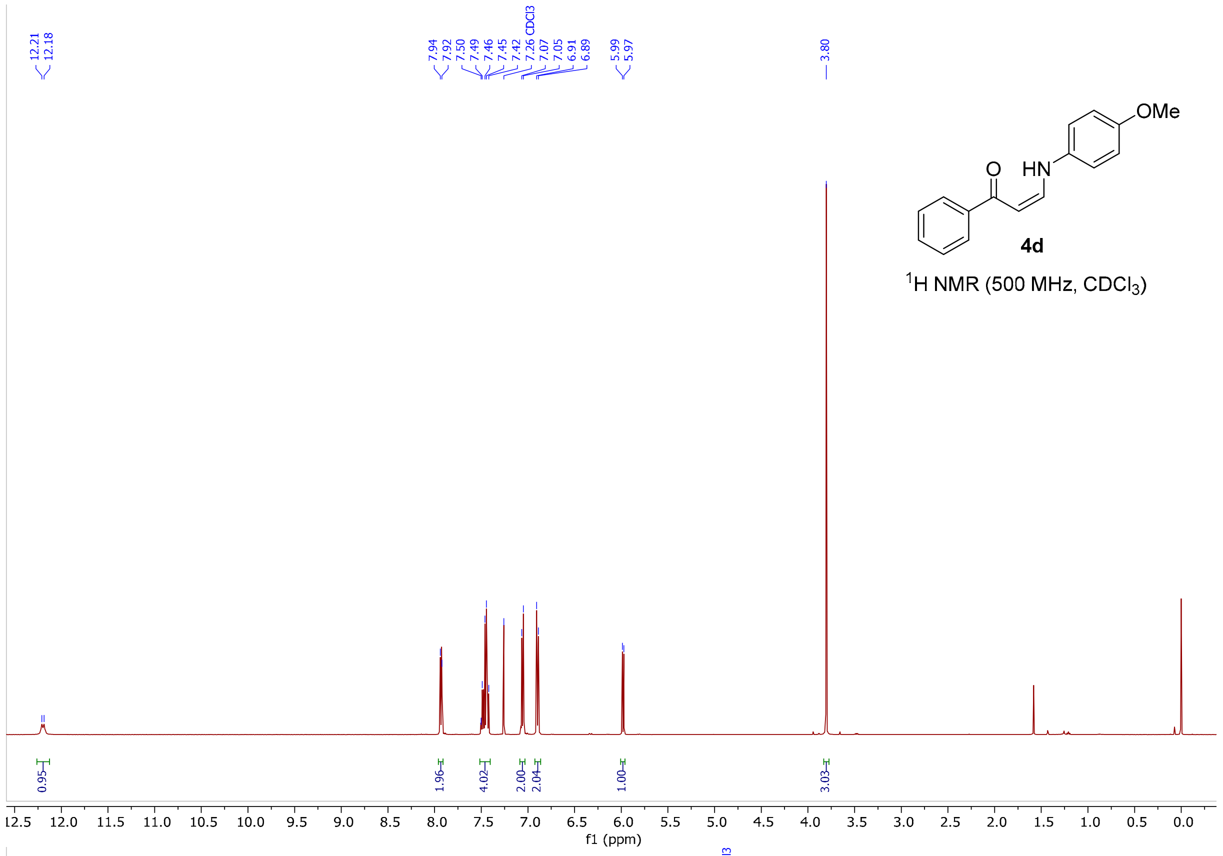

12.21
12.18
7.94
7.92
7.50
7.49
7.46
7.45
7.42
7.26 CDCl3
7.07
7.05
6.91
6.89
5.99
5.97
3.80
OMe
O
HN
4d
1H NMR (500 MHz, CDCl3)
0.95
1.96
4.02
2.00
2.04
1.00
3.03
f1 (ppm)

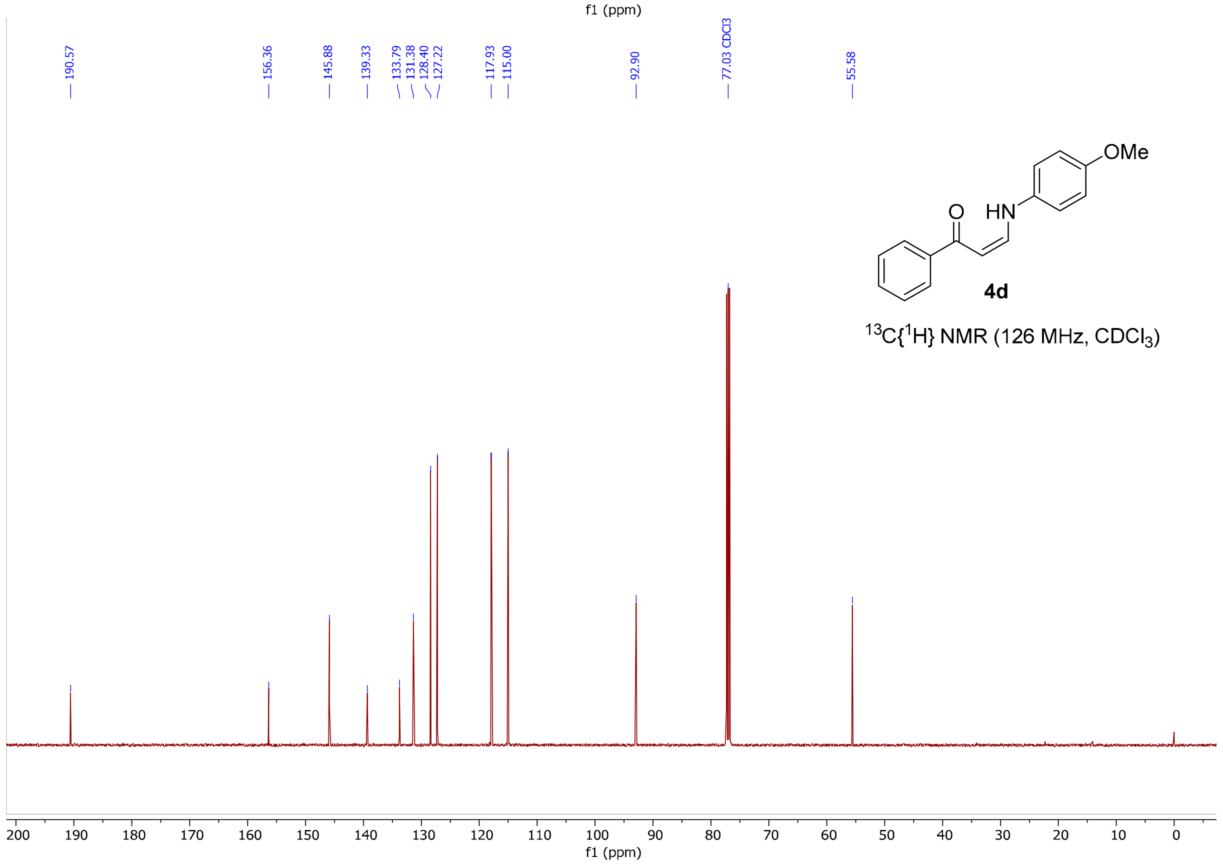

190.57
156.36
145.88
139.33
133.79
131.38
128.40
127.22
117.93
115.00
92.90
77.03 CDCl3
55.58
OMe
O
HN
4d
13C{1H} NMR (126 MHz, CDCl3)
f1 (ppm)

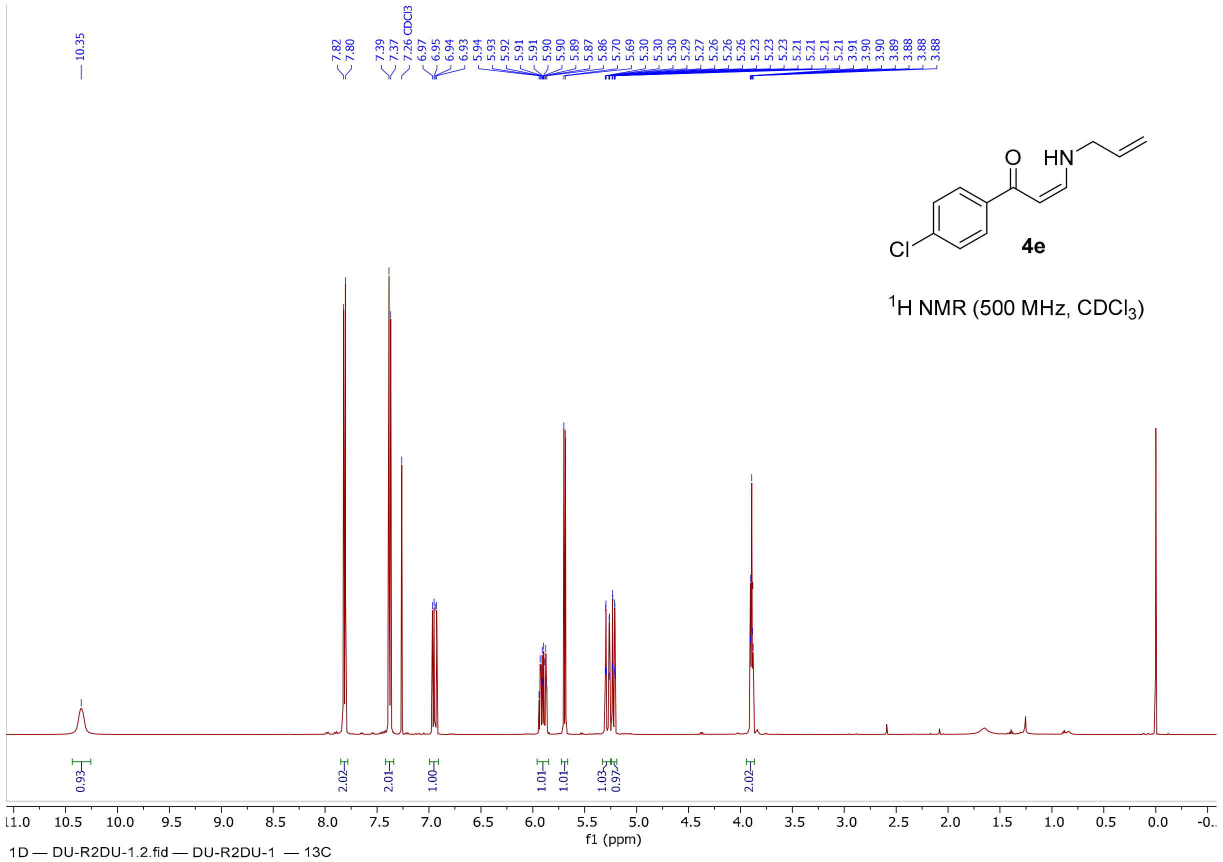

4e
^{1}H NMR (500 MHz, CDCl$_3$)
f1 (ppm)

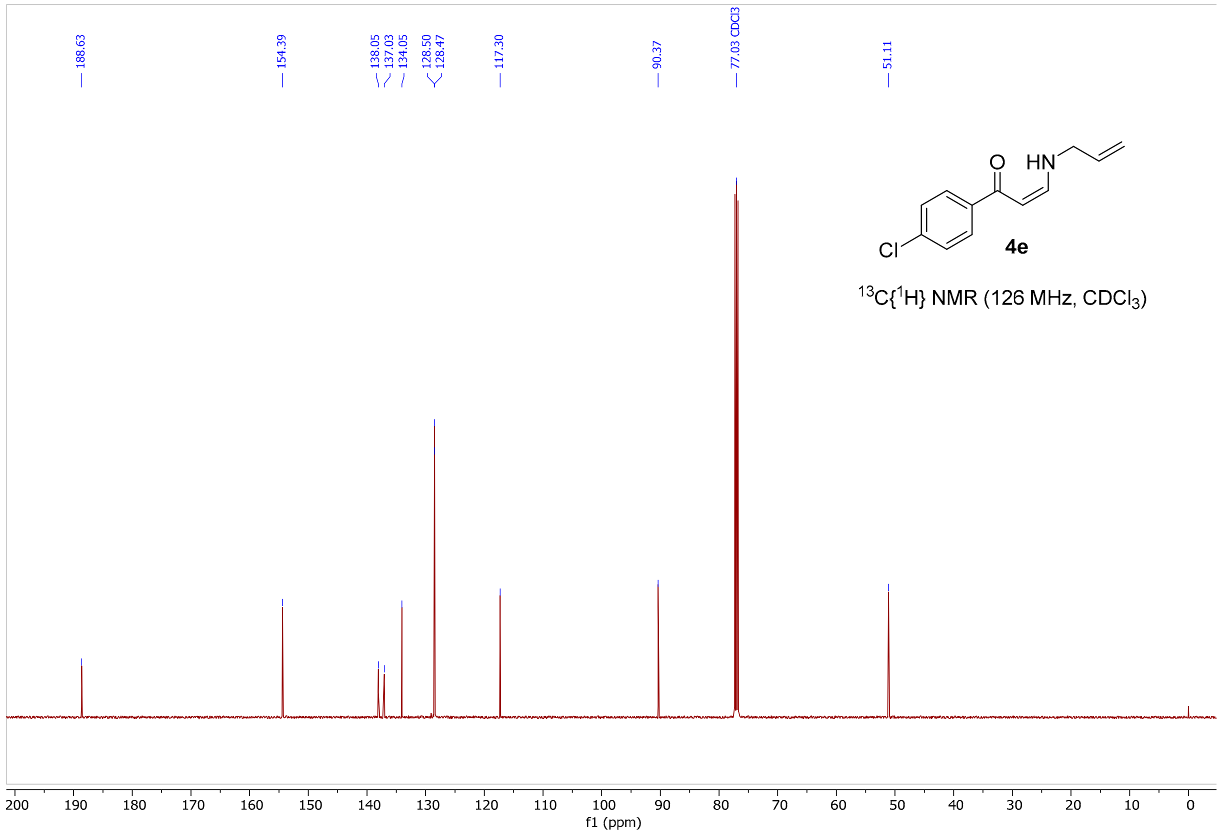

1D — DU-R2DU-1.2.fid — DU-R2DU-1 — 13C
4e
^{13}C{^{1}H} NMR (126 MHz, CDCl$_3$)
f1 (ppm)

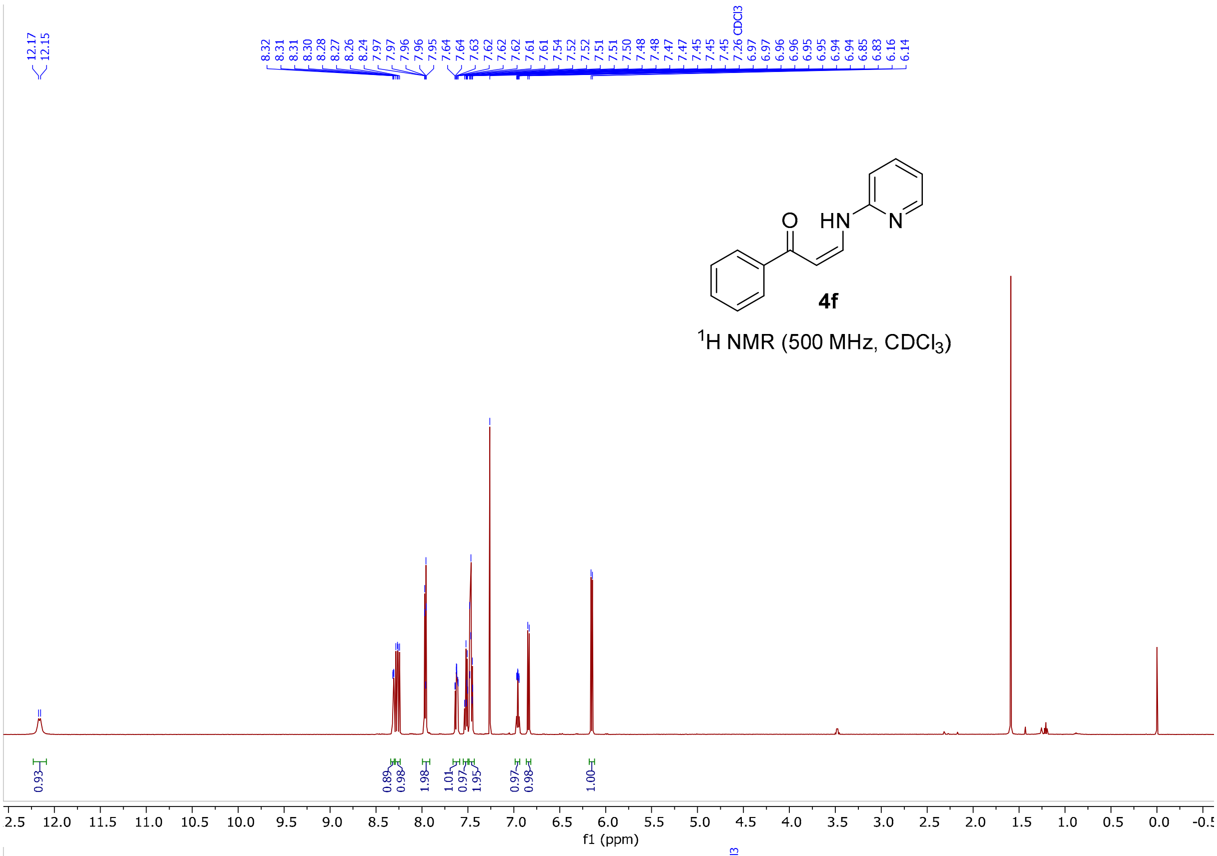
12.17
12.15
8.32 8.31 8.31 8.30 8.28 8.27 8.26 8.24 7.97 7.97 7.96 7.96 7.95 7.64 7.64 7.63 7.62 7.62 7.62 7.61 7.61 7.54 7.52 7.52 7.51 7.51 7.50 7.48 7.48 7.47 7.47 7.45 7.45 7.45 7.26 CDCl3 6.97 6.97 6.96 6.96 6.95 6.95 6.94 6.94 6.85 6.83 6.16 6.14
O
HN
N
4f
1H NMR (500 MHz, CDCl3)
0.93
0.89
0.98
1.98
1.01
0.97
1.95
0.97
0.98
1.00
f1 (ppm)

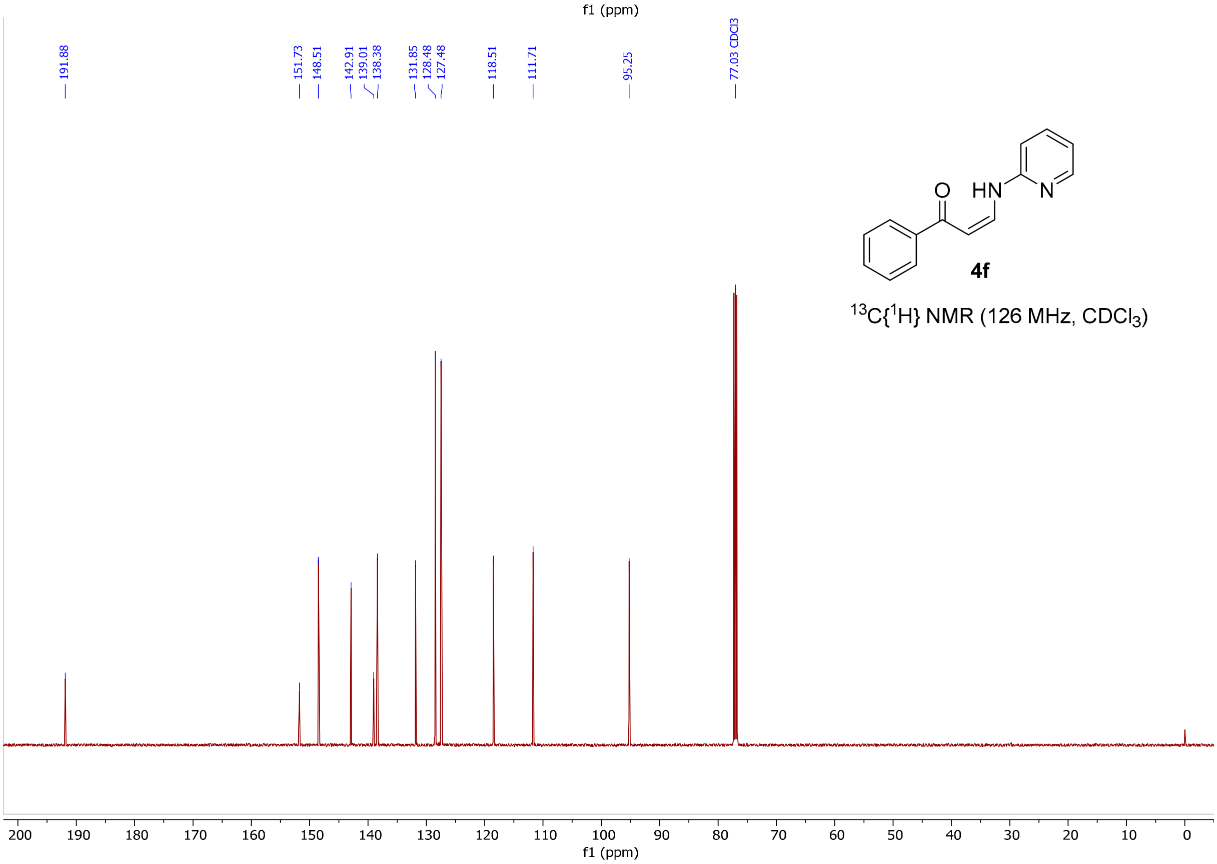
191.88
151.73
148.51
142.91
139.01
138.38
131.85
128.48
127.48
118.51
111.71
95.25
77.03 CDCl3
O
HN
N
4f
13C{1H} NMR (126 MHz, CDCl3)
f1 (ppm)

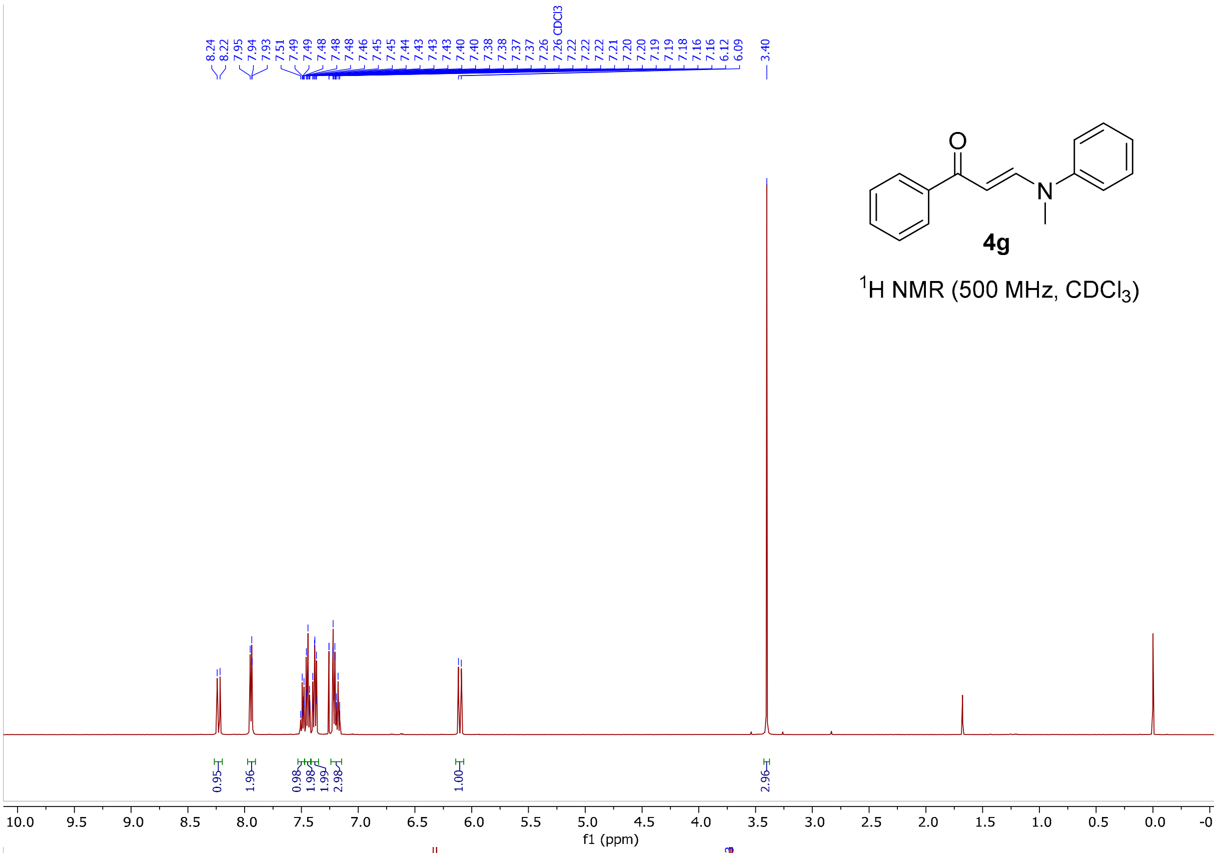
4g
1H NMR (500 MHz, CDCl3)
f1 (ppm)

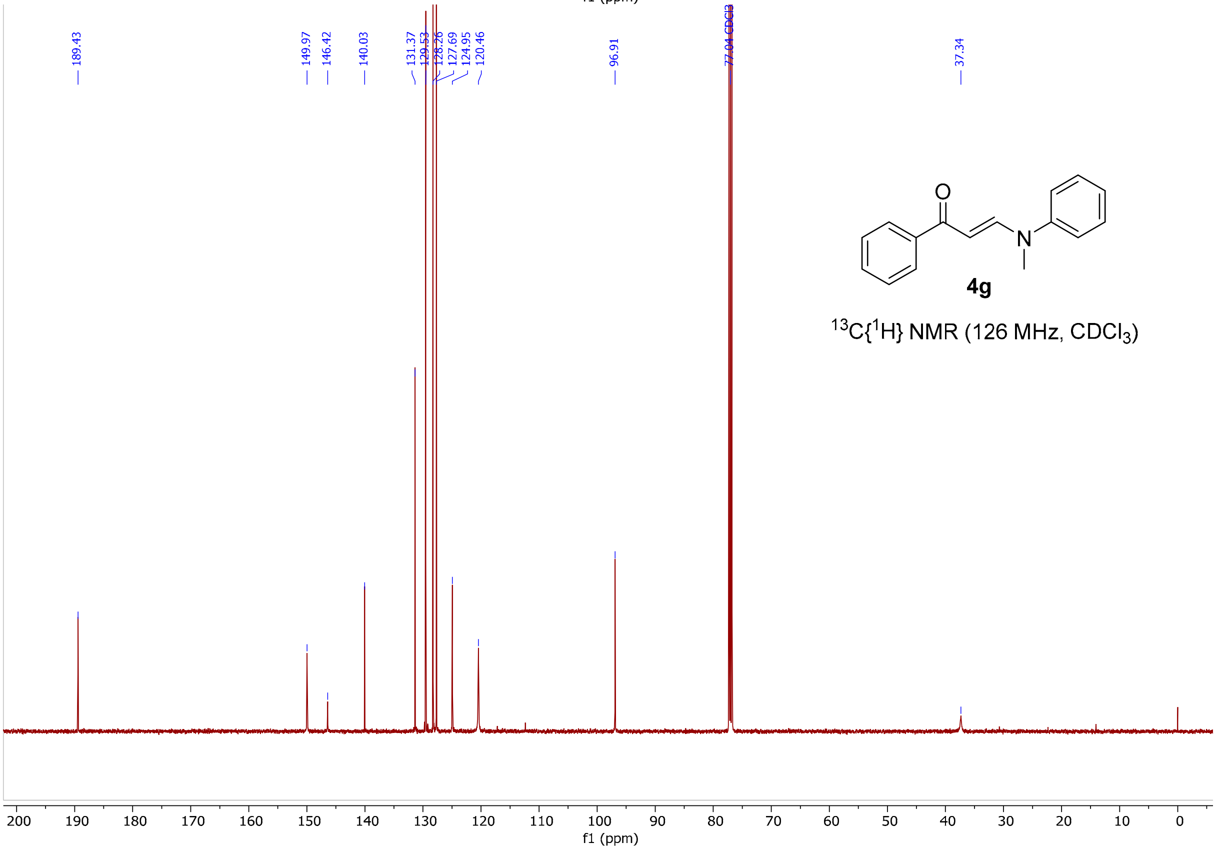
189.43
149.97
146.42
140.03
131.37
124.95
120.46
96.91
37.34
4g
13C{1H} NMR (126 MHz, CDCl3)
f1 (ppm)

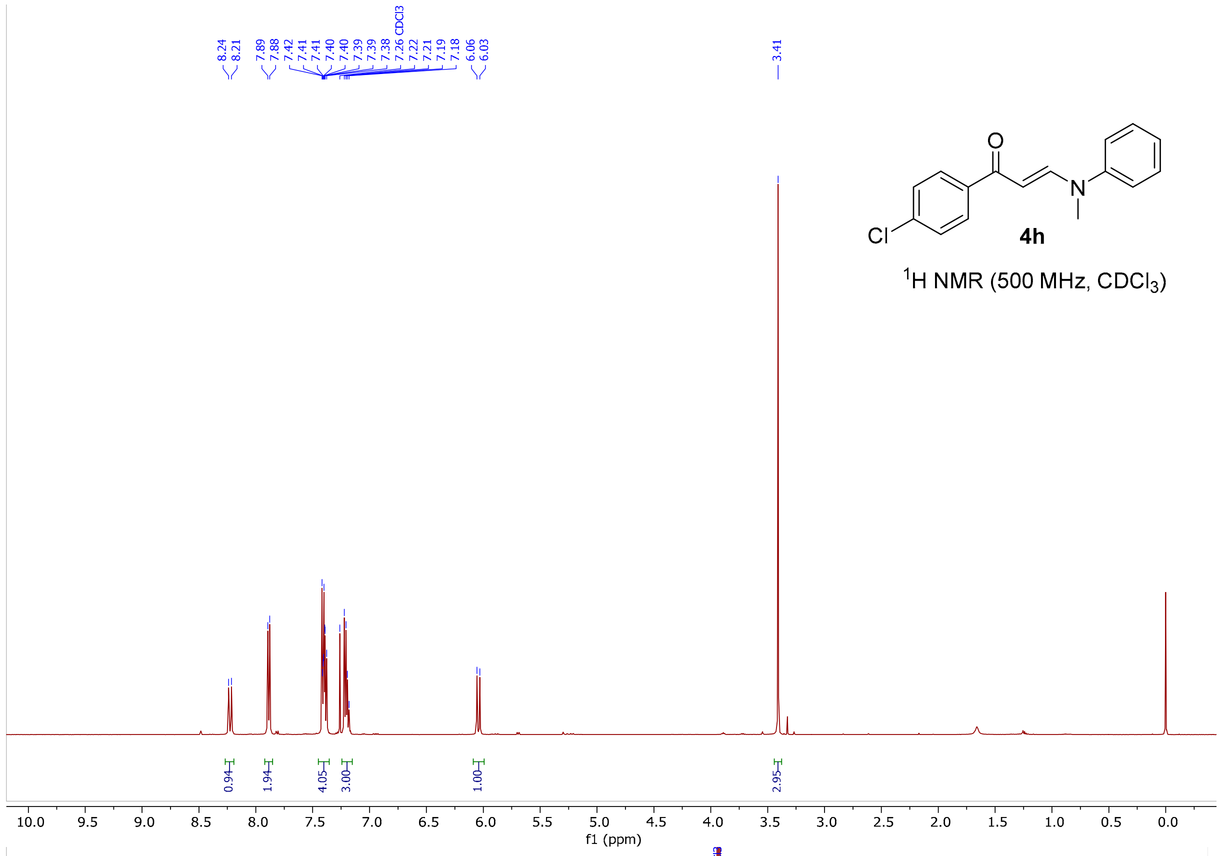

8.24
8.21
7.89
7.88
7.42
7.41
7.41
7.40
7.40
7.39
7.39
7.38
7.26 CDCl3
7.22
7.21
7.21
7.19
7.18
6.06
6.03
3.41
Cl
O
N
4h
1H NMR (500 MHz, CDCl3)
0.94
1.94
4.05
3.00
1.00
2.95
10.0
9.5
9.0
8.5
8.0
7.5
7.0
6.5
6.0
5.5
5.0
4.5
4.0
3.5
3.0
2.5
2.0
1.5
1.0
0.5
0.0
f1 (ppm)

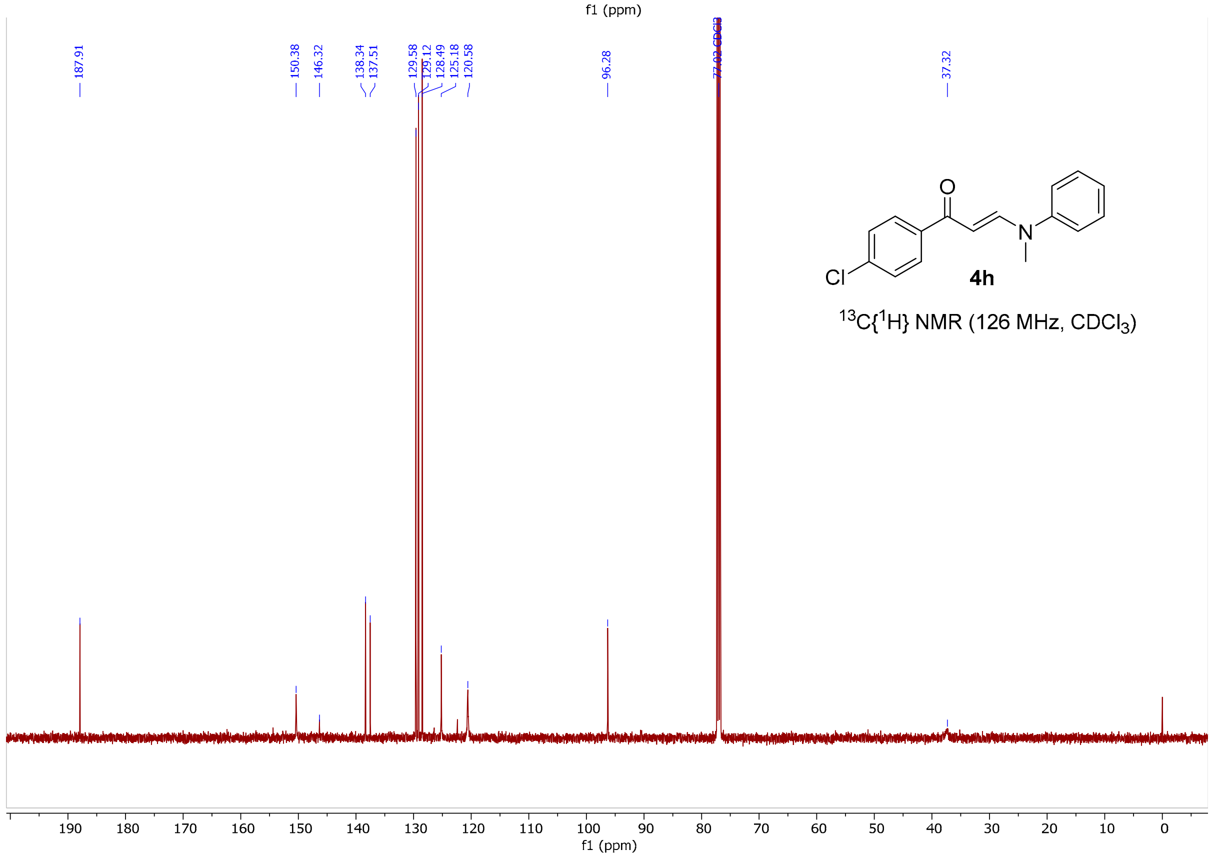

187.91
150.38
146.32
138.34
137.51
129.58
129.12
128.49
125.18
120.58
96.28
77.03 CDCl3
37.32
Cl
O
N
4h
13C{1H} NMR (126 MHz, CDCl3)
190
180
170
160
150
140
130
120
110
100
90
80
70
60
50
40
30
20
10
0
f1 (ppm)

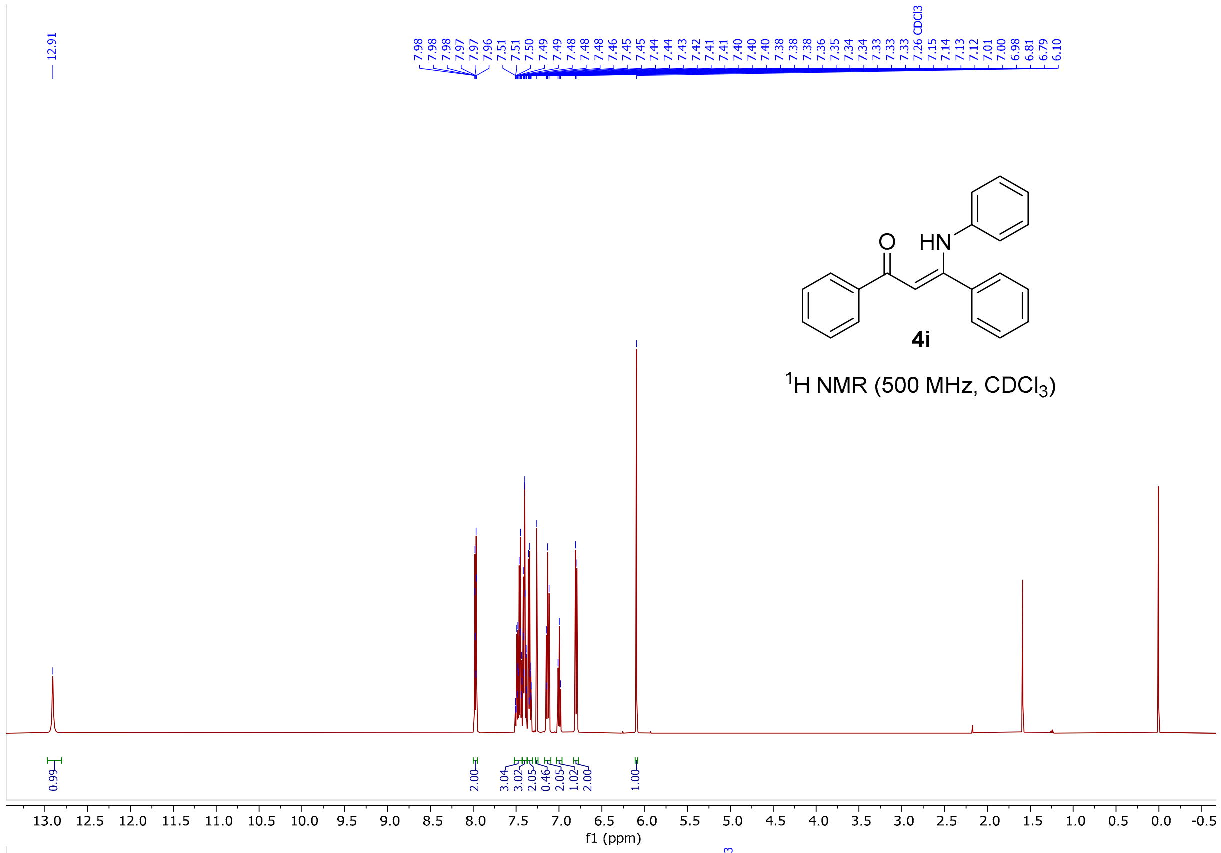

4i
1H NMR (500 MHz, CDCl3)
f1 (ppm)

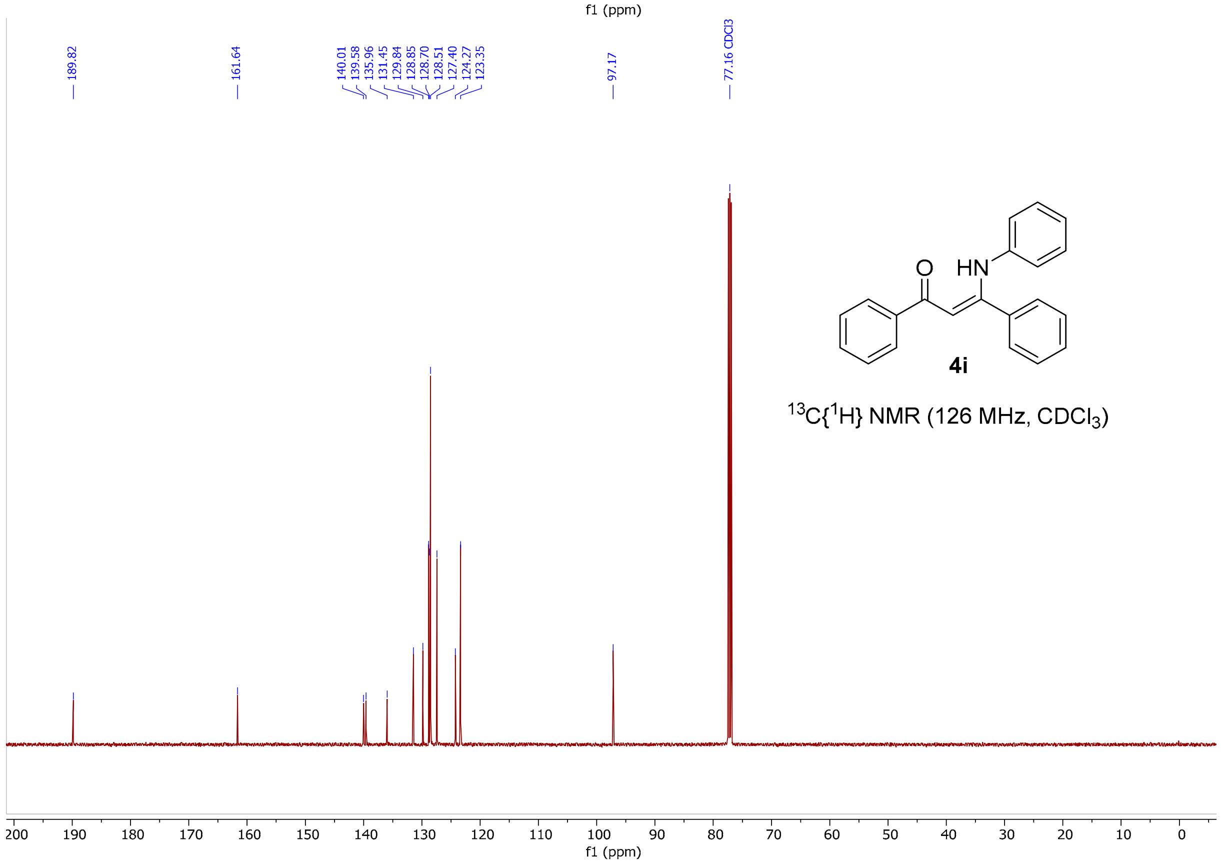

4i
13C{1H} NMR (126 MHz, CDCl3)
f1 (ppm)

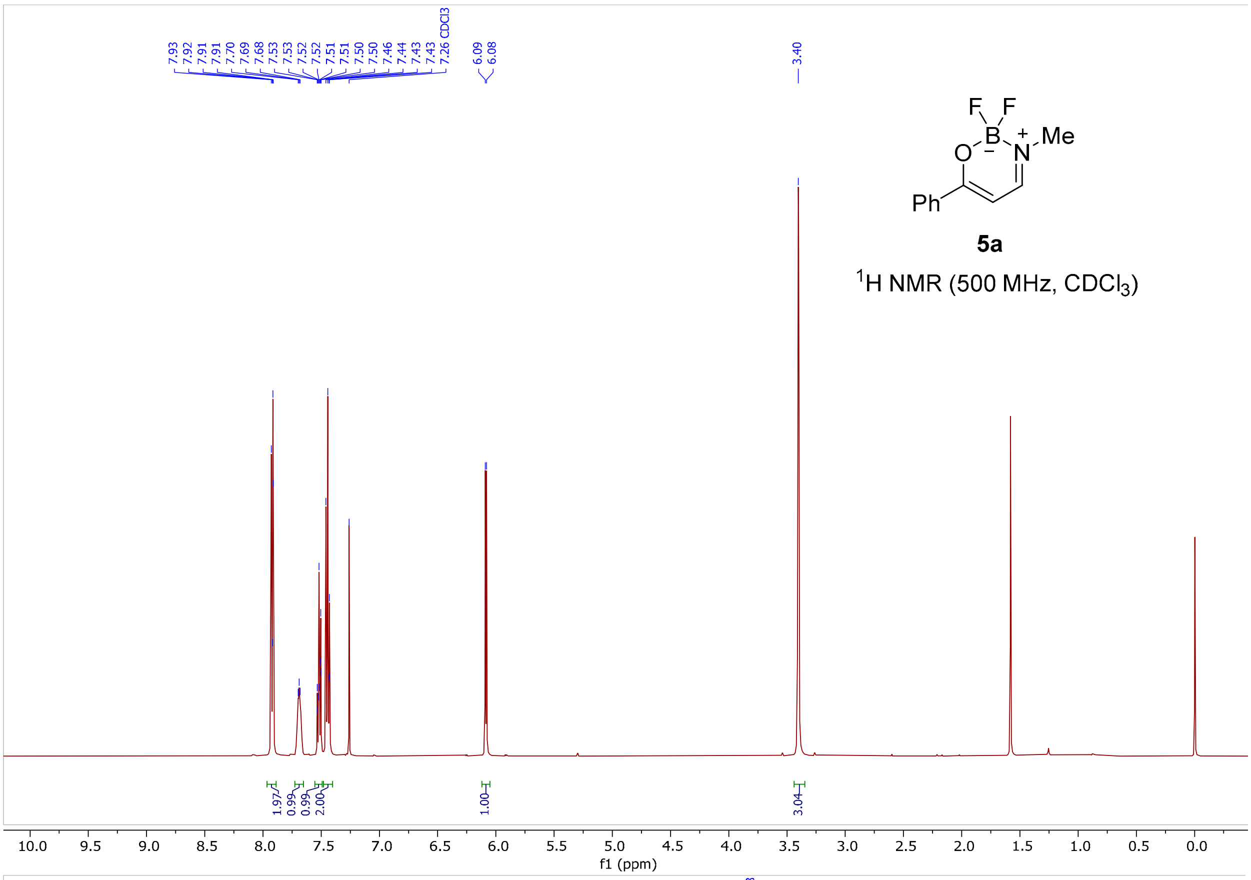
7.93
7.92
7.91
7.91
7.70
7.69
7.68
7.53
7.53
7.52
7.52
7.51
7.51
7.50
7.50
7.46
7.44
7.43
7.43
7.26 CDCl3
6.09
6.08
3.40
F F
B
+
Me
O
N
Ph
5a
1H NMR (500 MHz, CDCl3)
1.97
0.99
0.99
2.00
1.00
3.04
10.0 9.5 9.0 8.5 8.0 7.5 7.0 6.5 6.0 5.5 5.0 4.5 4.0 3.5 3.0 2.5 2.0 1.5 1.0 0.5 0.0
f1 (ppm)

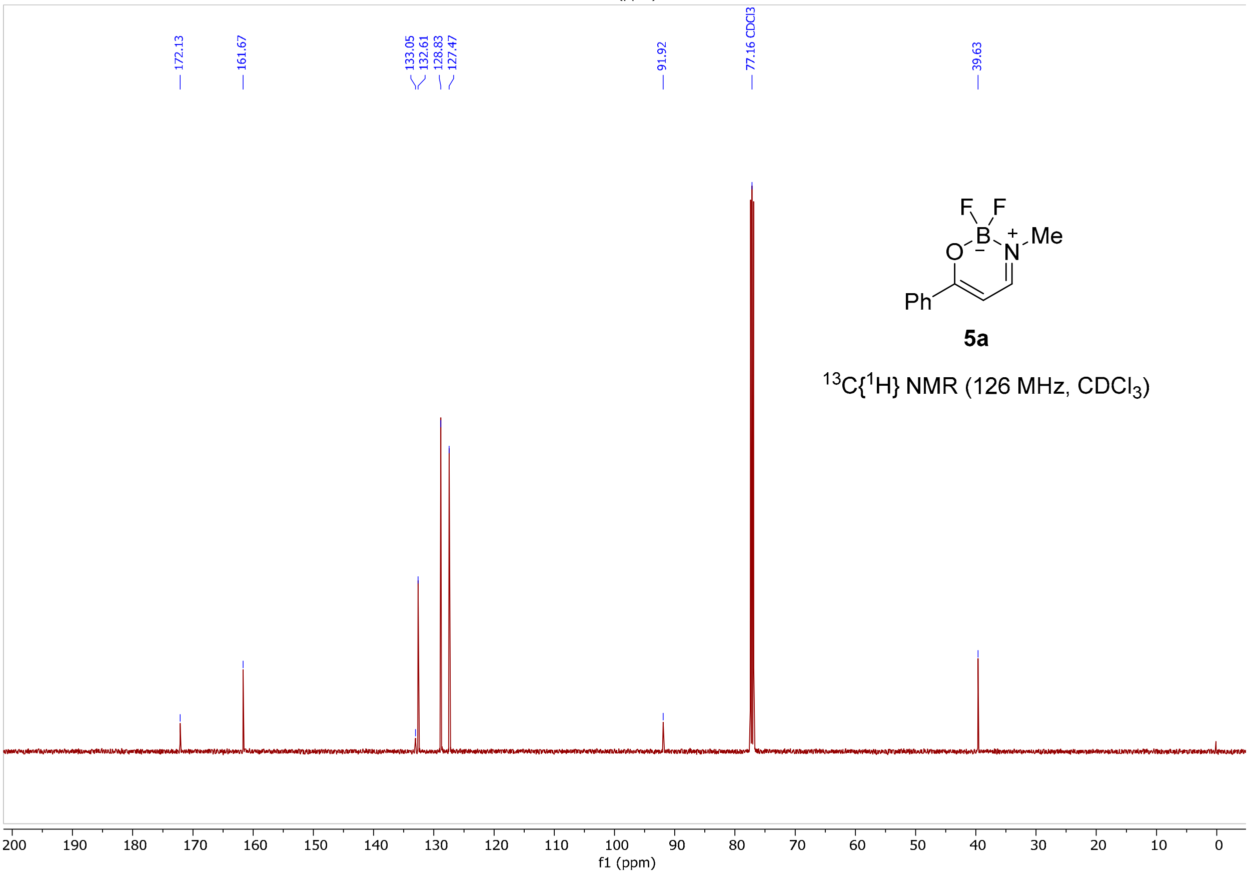
172.13
161.67
133.05
132.61
128.83
127.47
91.92
77.16 CDCl3
39.63
F F
B
+
Me
O
N
Ph
5a
13C{1H} NMR (126 MHz, CDCl3)
200 190 180 170 160 150 140 130 120 110 100 90 80 70 60 50 40 30 20 10 0
f1 (ppm)

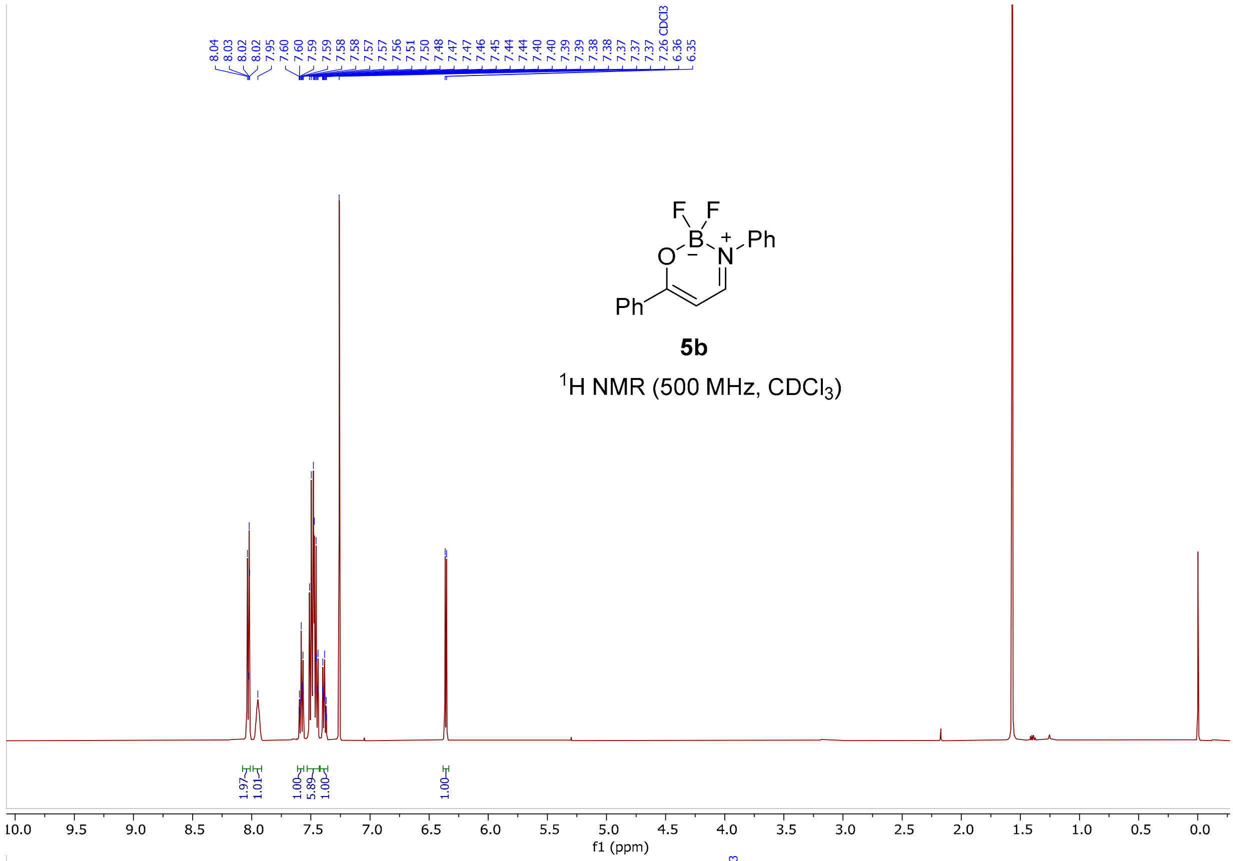
8.04
8.03
8.02
8.02
7.95
7.60
7.60
7.59
7.59
7.58
7.58
7.57
7.57
7.56
7.51
7.50
7.48
7.47
7.47
7.46
7.45
7.44
7.44
7.40
7.40
7.39
7.39
7.38
7.38
7.37
7.37
7.37
7.26 CDCl3
6.36
6.35
1.97
1.01
1.00
5.89
1.00
1.00
f1 (ppm)
5b
^{1}H NMR (500 MHz, CDCl$_3$)

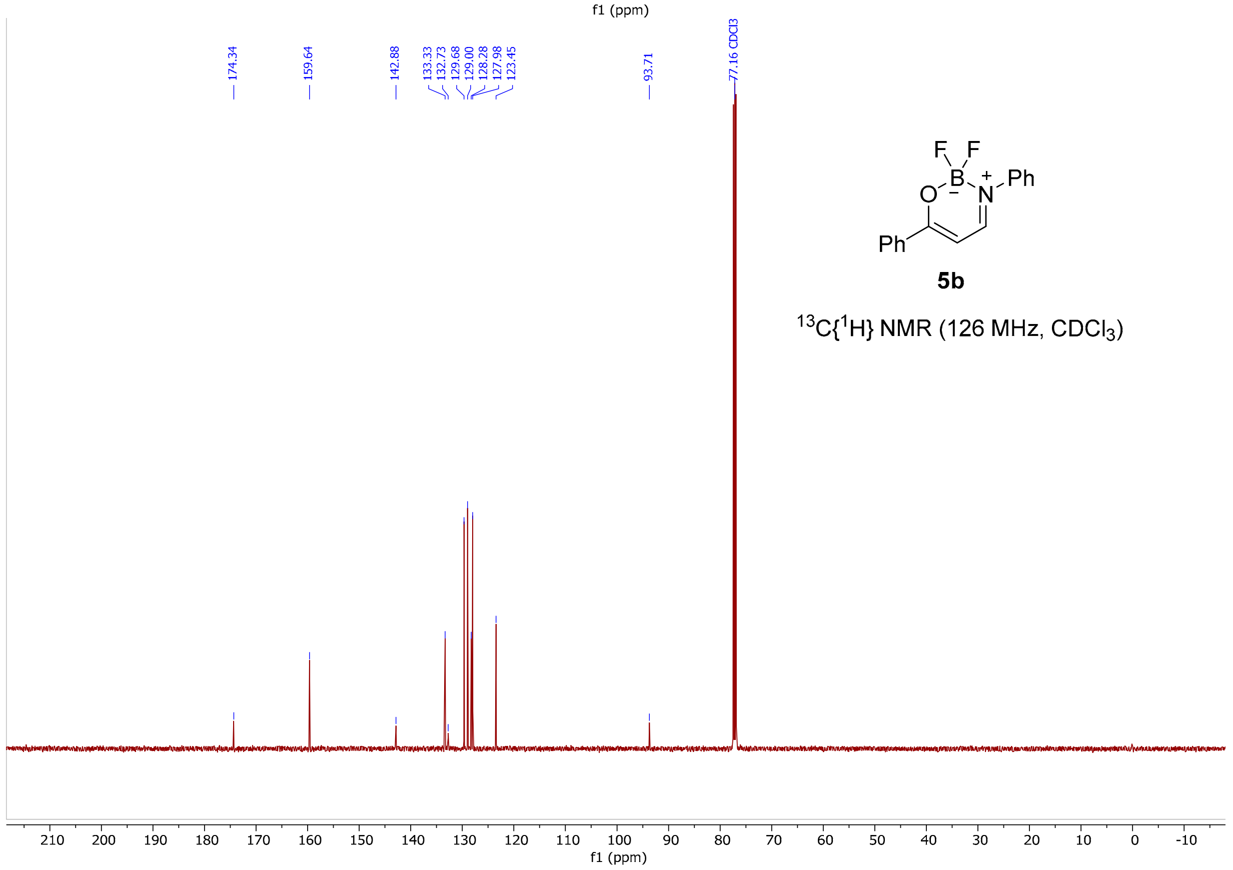
174.34
159.64
142.88
133.33
132.73
129.68
129.00
128.28
127.98
123.45
93.71
77.16 CDCl3
f1 (ppm)
5b
^{13}C{^{1}H} NMR (126 MHz, CDCl$_3$)

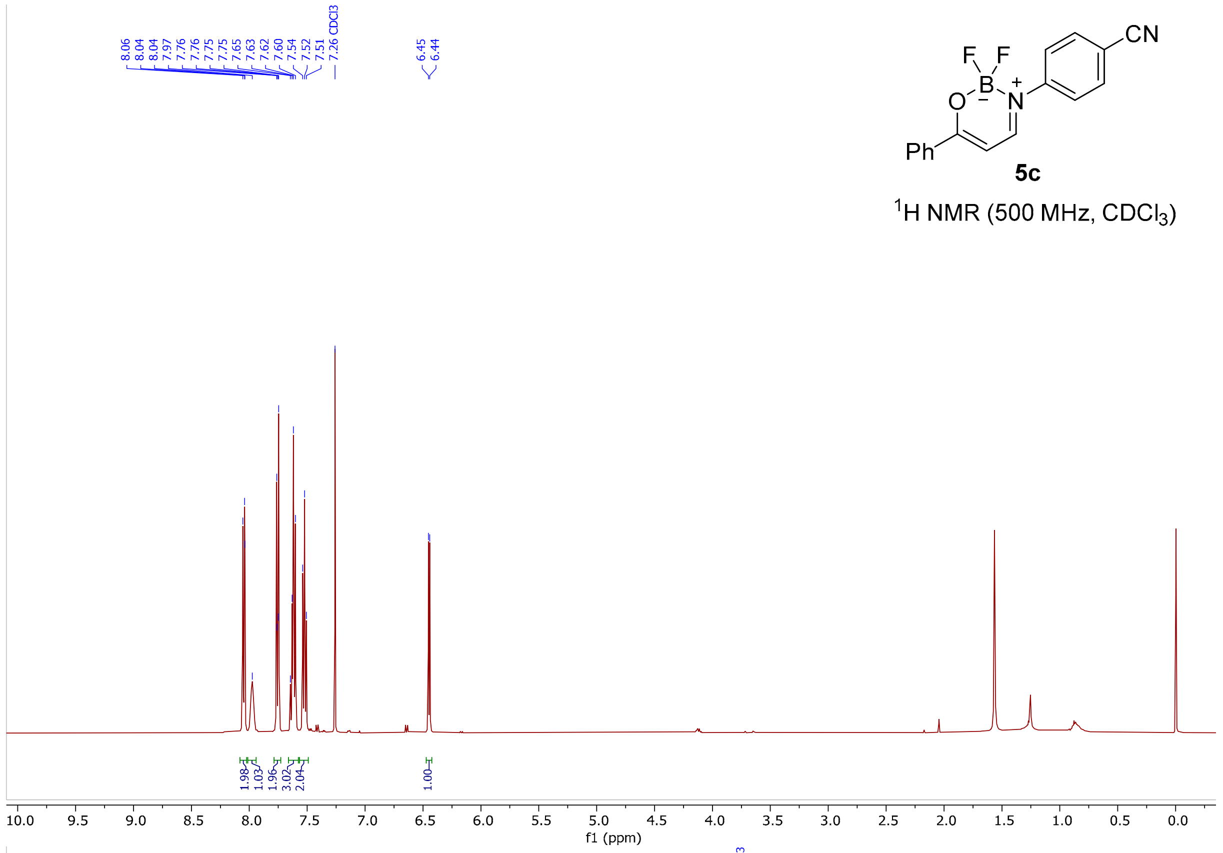
8.06
8.04
8.04
7.97
7.76
7.76
7.75
7.75
7.65
7.63
7.63
7.62
7.60
7.54
7.52
7.51
7.26 CDCl3
6.45
6.44
F F
B
N
O
Ph
CN
5c
1H NMR (500 MHz, CDCl3)
1.98
1.03
1.96
3.02
2.04
1.00
f1 (ppm)

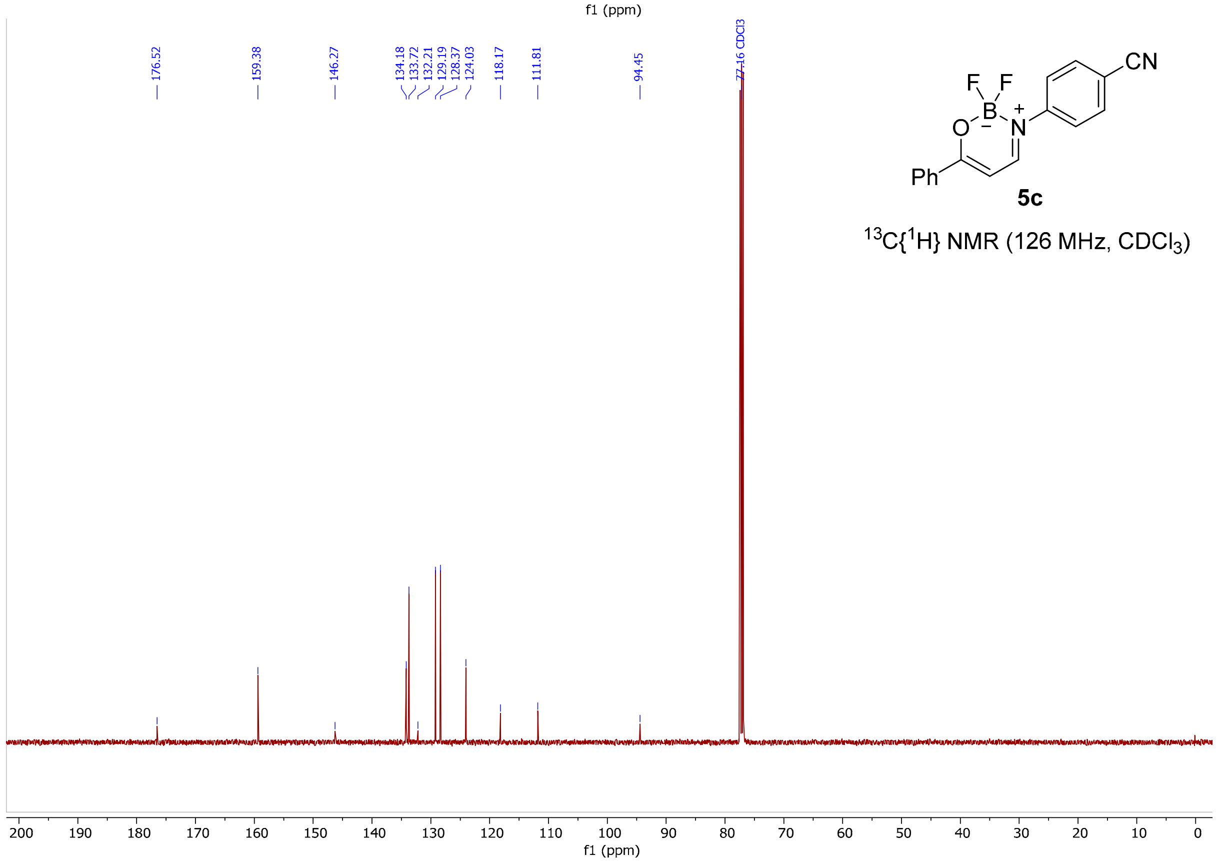
176.52
159.38
146.27
134.18
133.72
132.21
129.19
128.37
124.03
118.17
111.81
94.45
77.16 CDCl3
F F
B
N
O
Ph
CN
5c
13C{1H} NMR (126 MHz, CDCl3)
f1 (ppm)

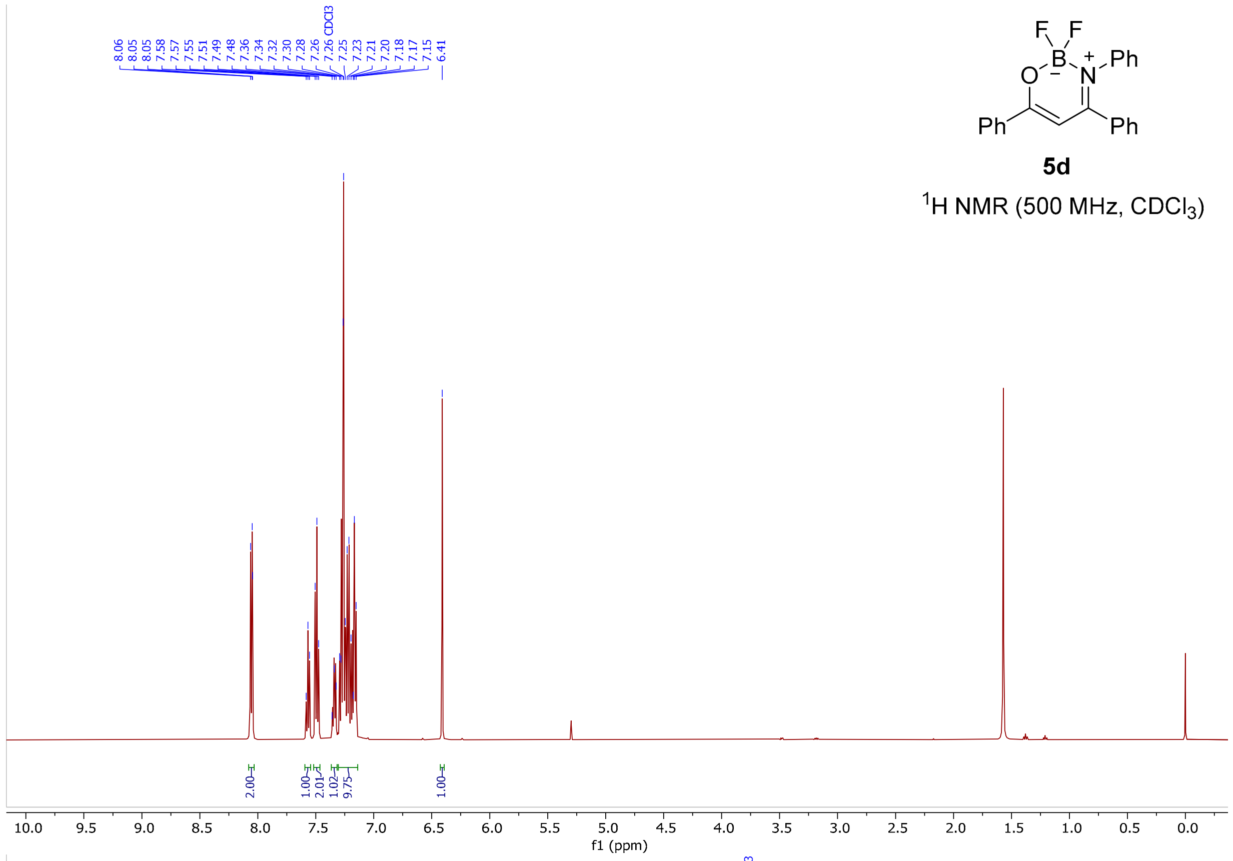
5d
1H NMR (500 MHz, CDCl3)
f1 (ppm)

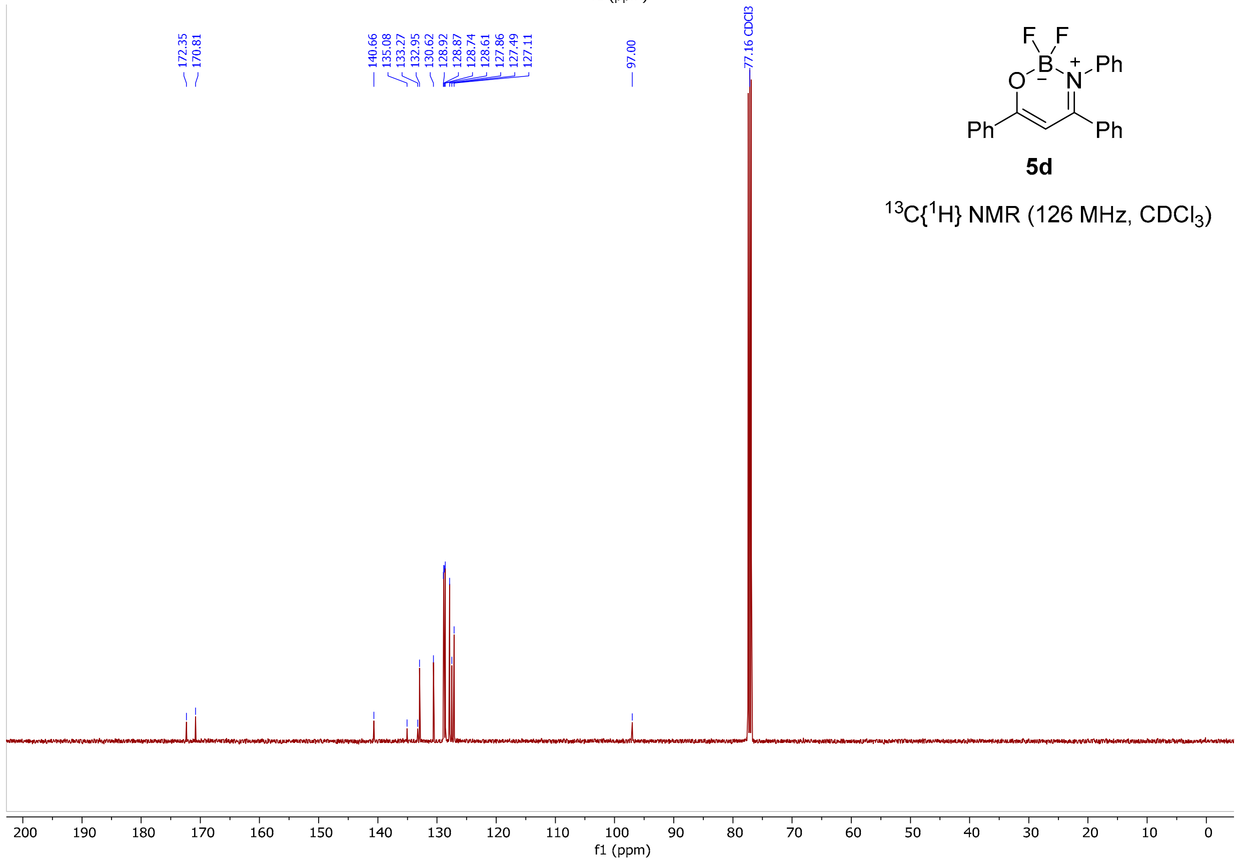
5d
13C{1H} NMR (126 MHz, CDCl3)
f1 (ppm)

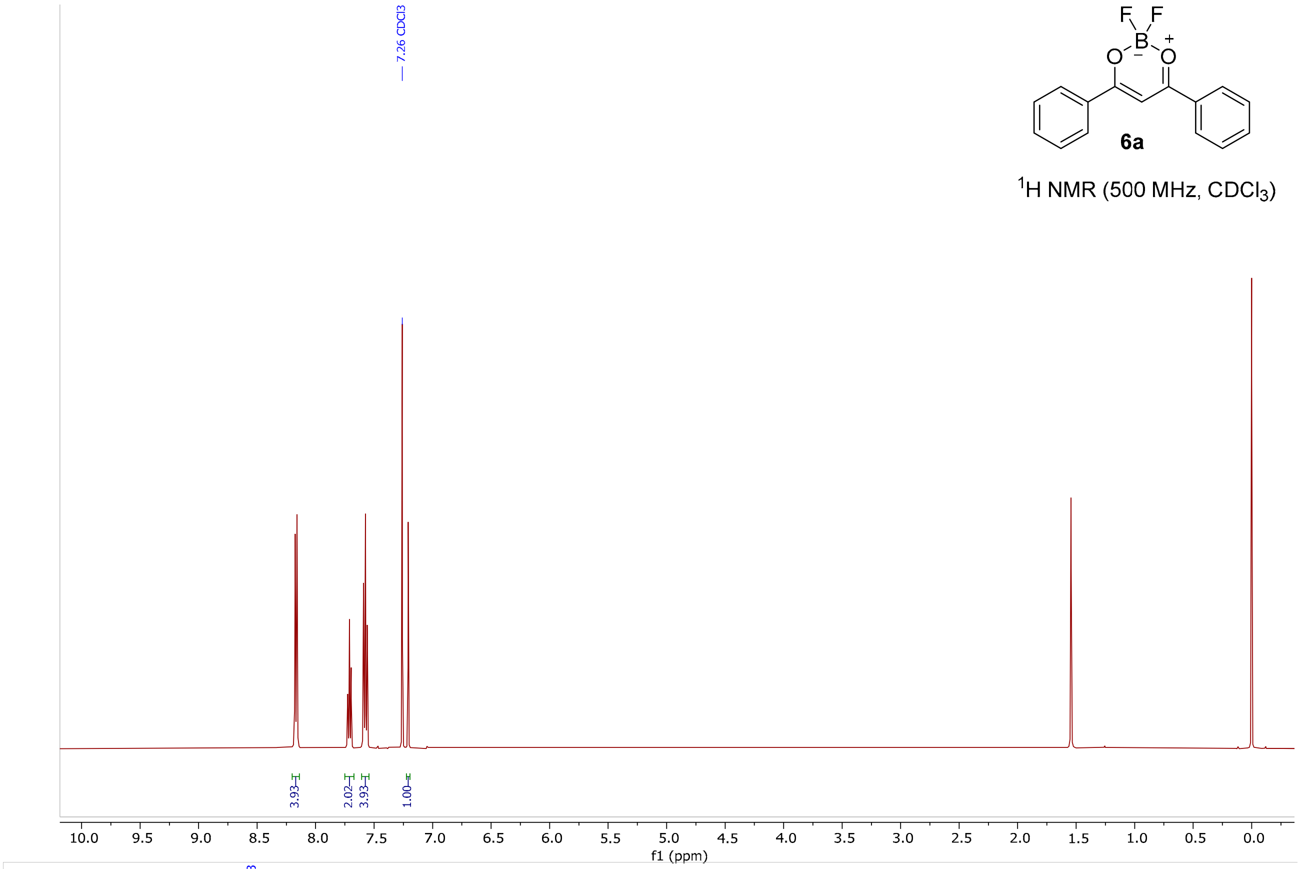
7.26 CDCl3
6a
1H NMR (500 MHz, CDCl3)
3.93
2.02
3.93
1.00
f1 (ppm)

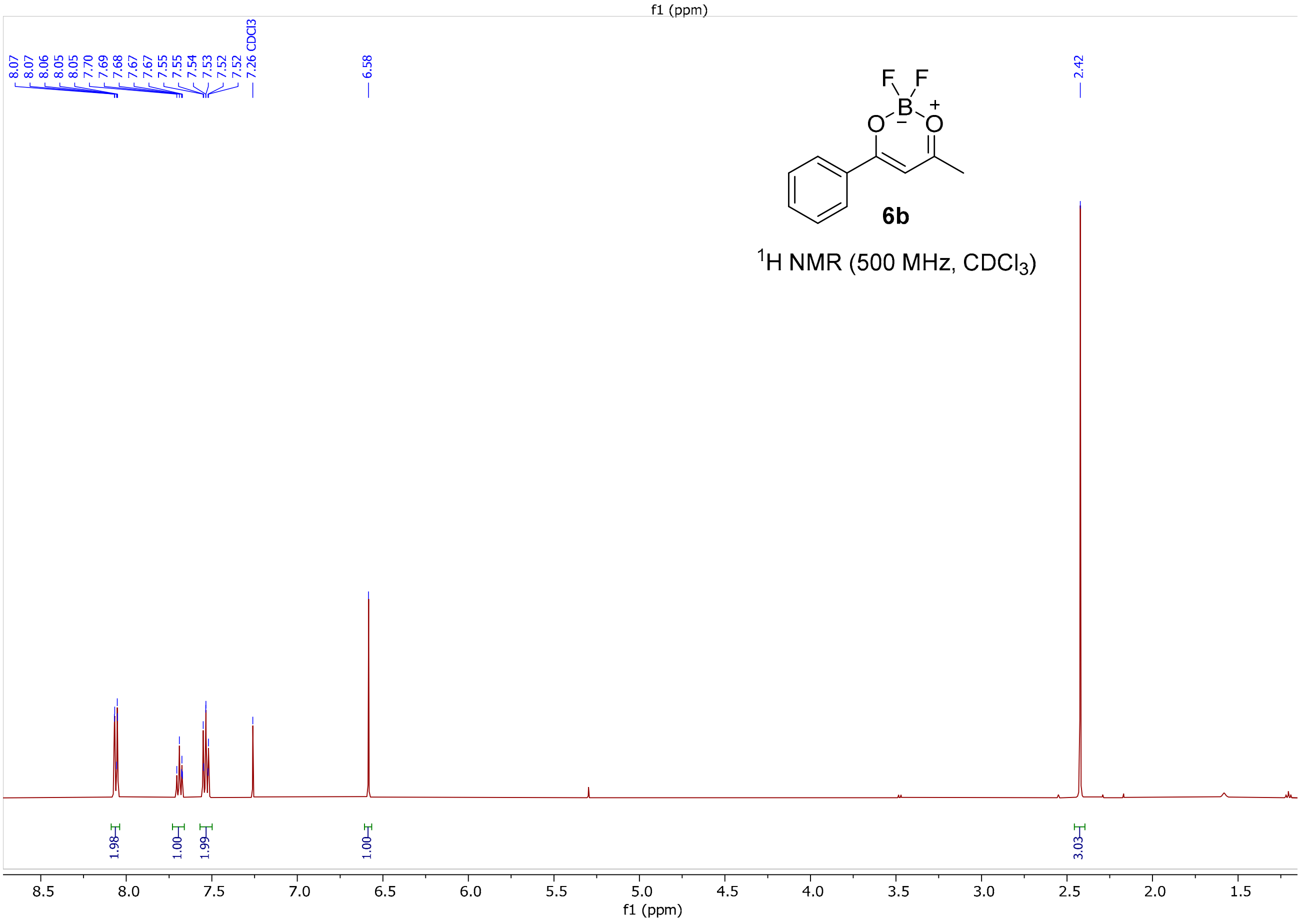
8.07
8.07
8.06
8.05
8.05
7.70
7.69
7.68
7.67
7.67
7.55
7.54
7.53
7.52
7.52
7.26 CDCl3
6.58
2.42
6b
1H NMR (500 MHz, CDCl3)
1.98
1.00
1.99
1.00
3.03
f1 (ppm)

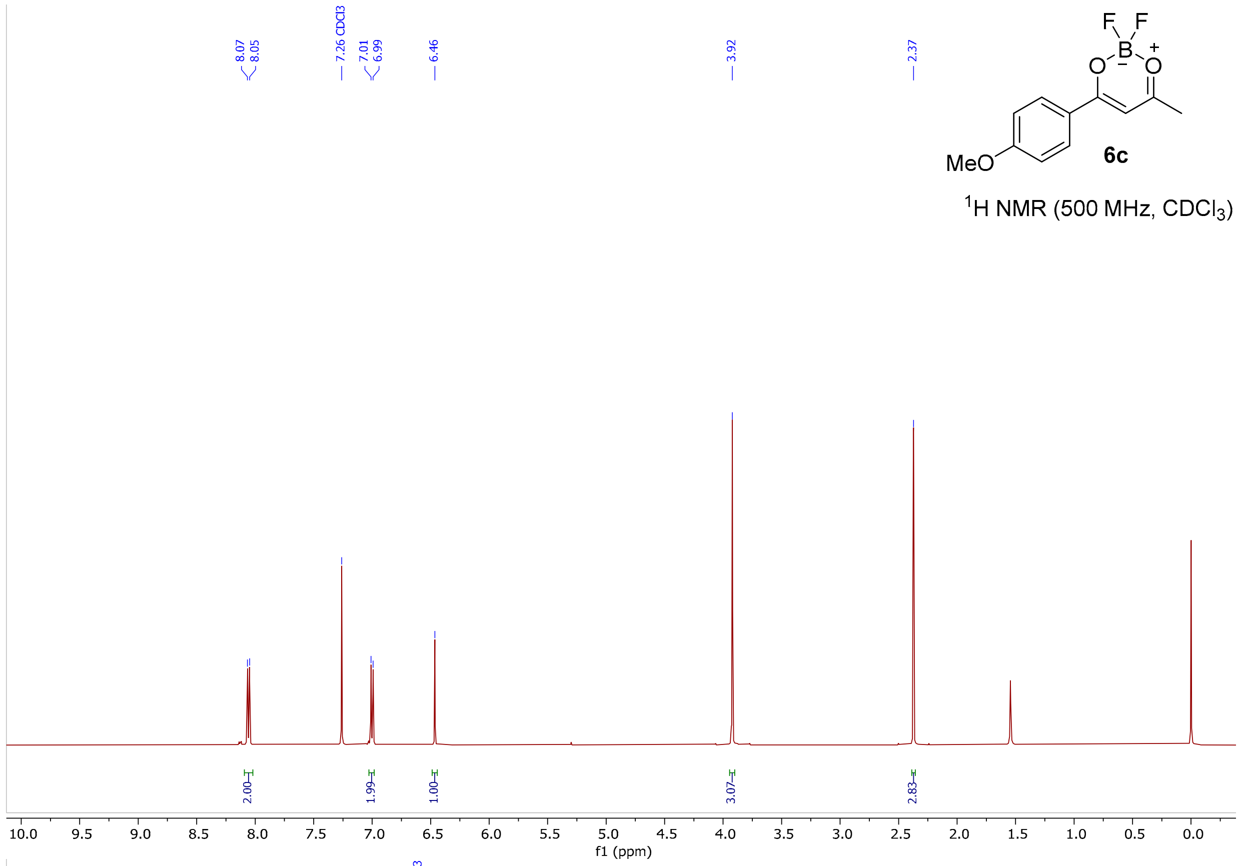
6c
^{1}H NMR (500 MHz, CDCl$_3$)
MeO
f1 (ppm)

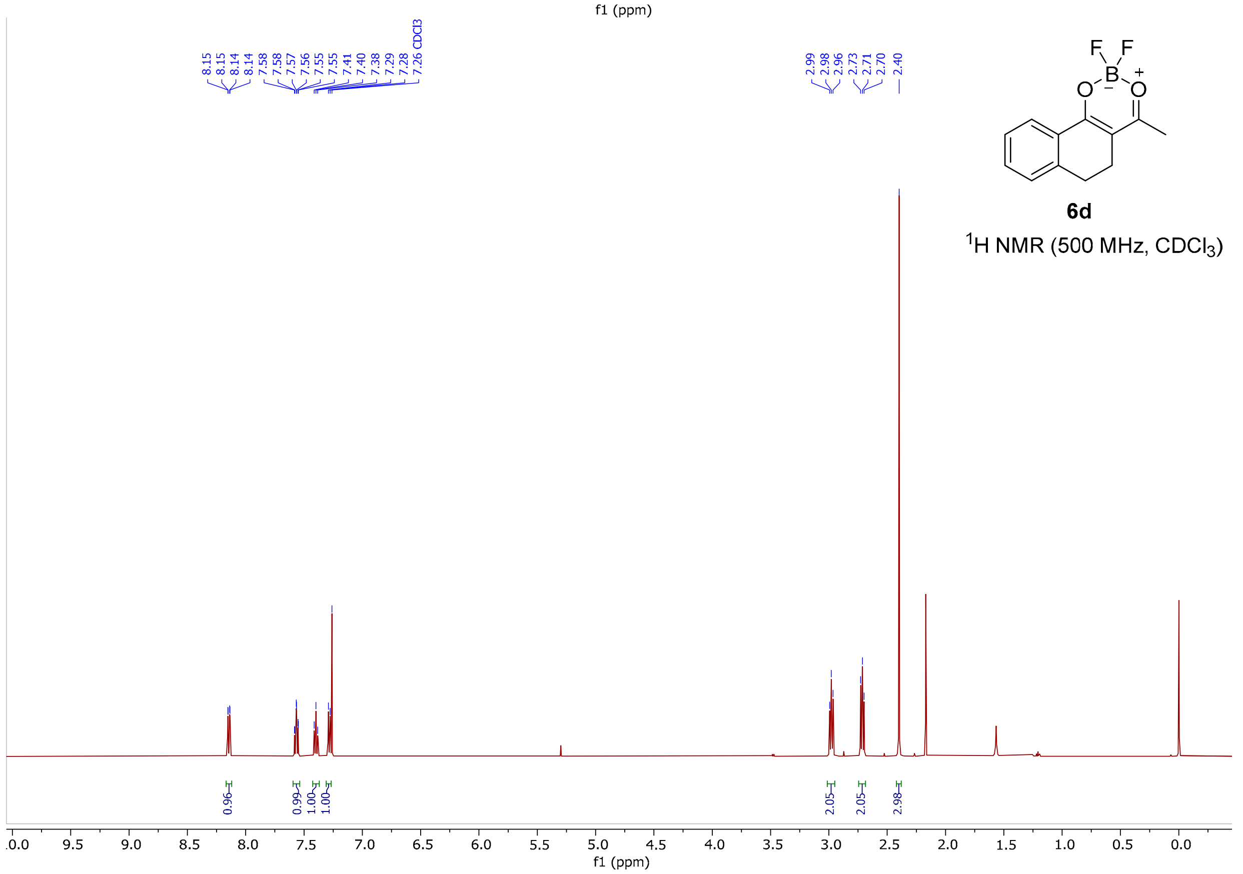
6d
^{1}H NMR (500 MHz, CDCl$_3$)
f1 (ppm)

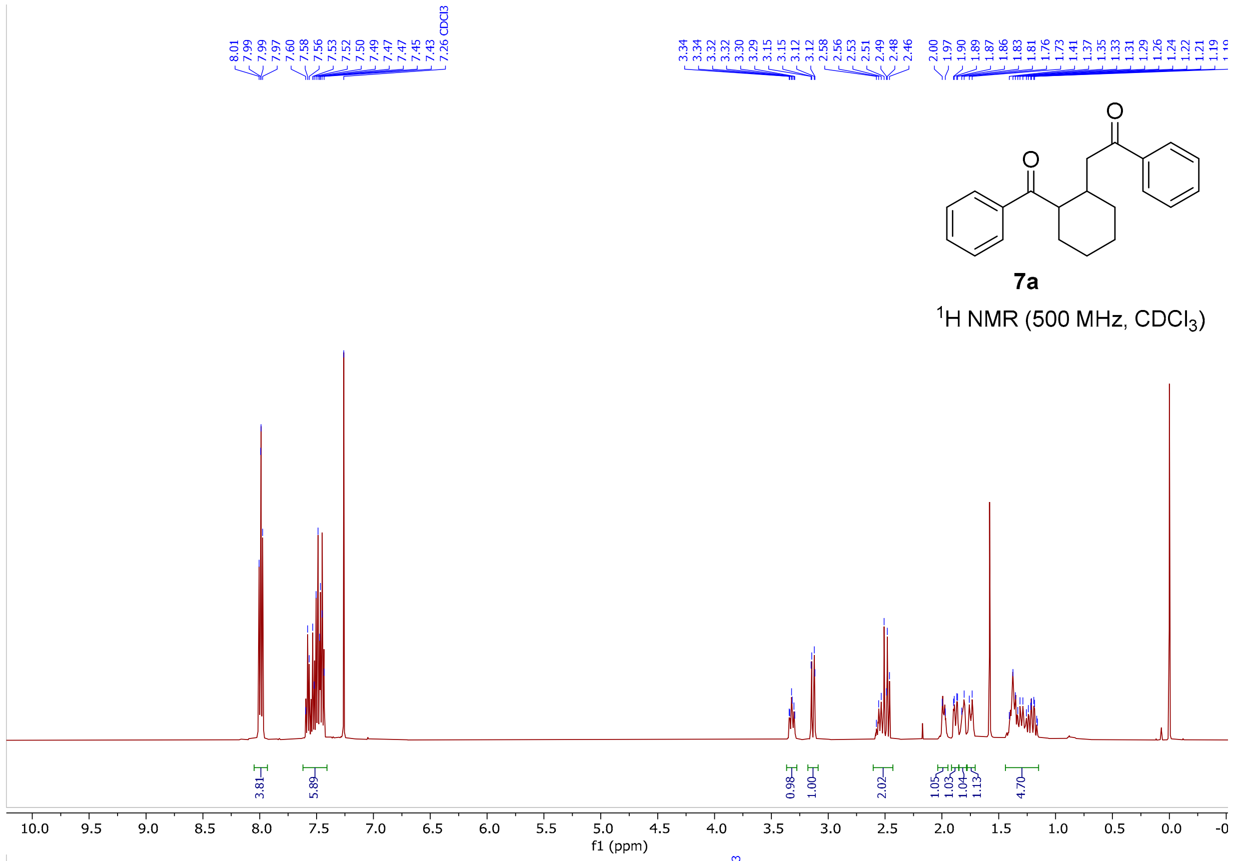

7a
^{1}H NMR (500 MHz, CDCl$_3$)
f1 (ppm)

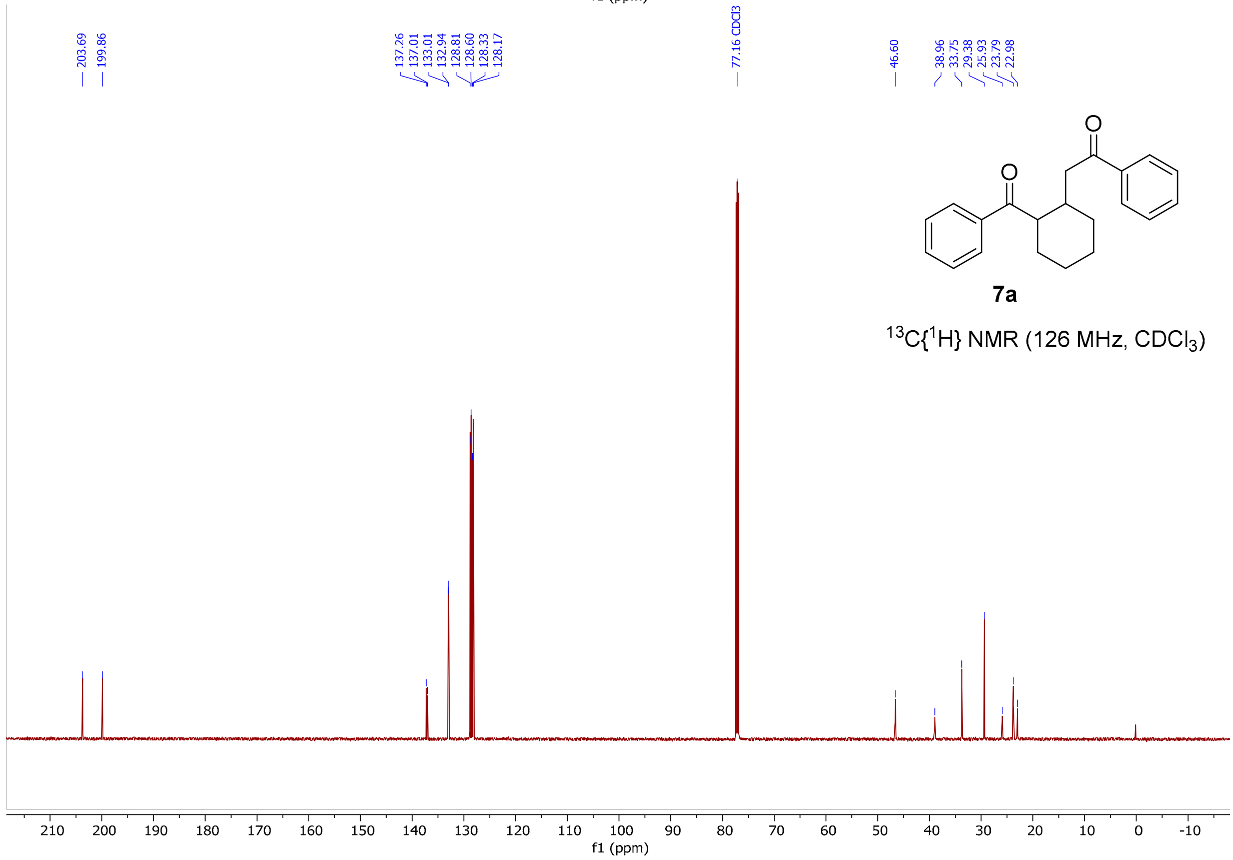

7a
^{13}C{^{1}H} NMR (126 MHz, CDCl$_3$)
f1 (ppm)

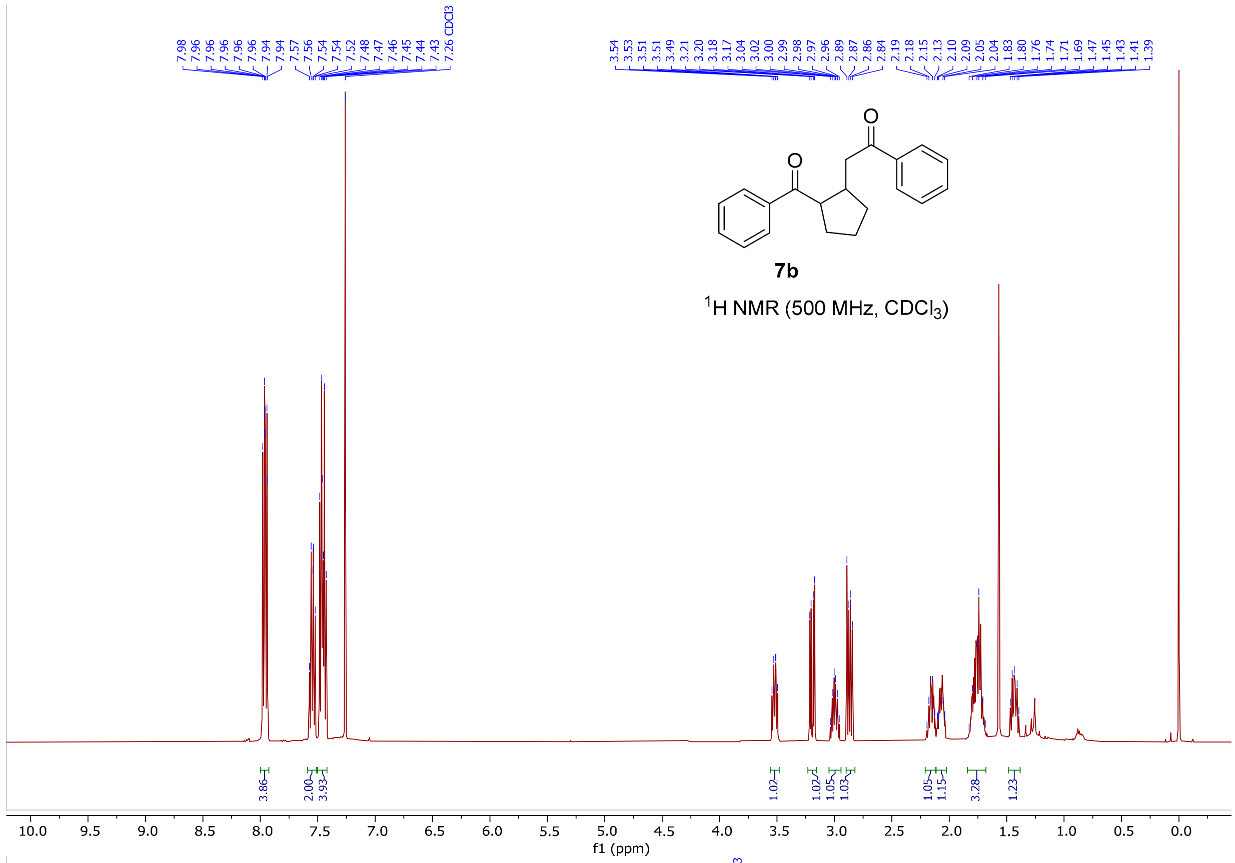
7b
^{1}H NMR (500 MHz, CDCl$_3$)
f1 (ppm)

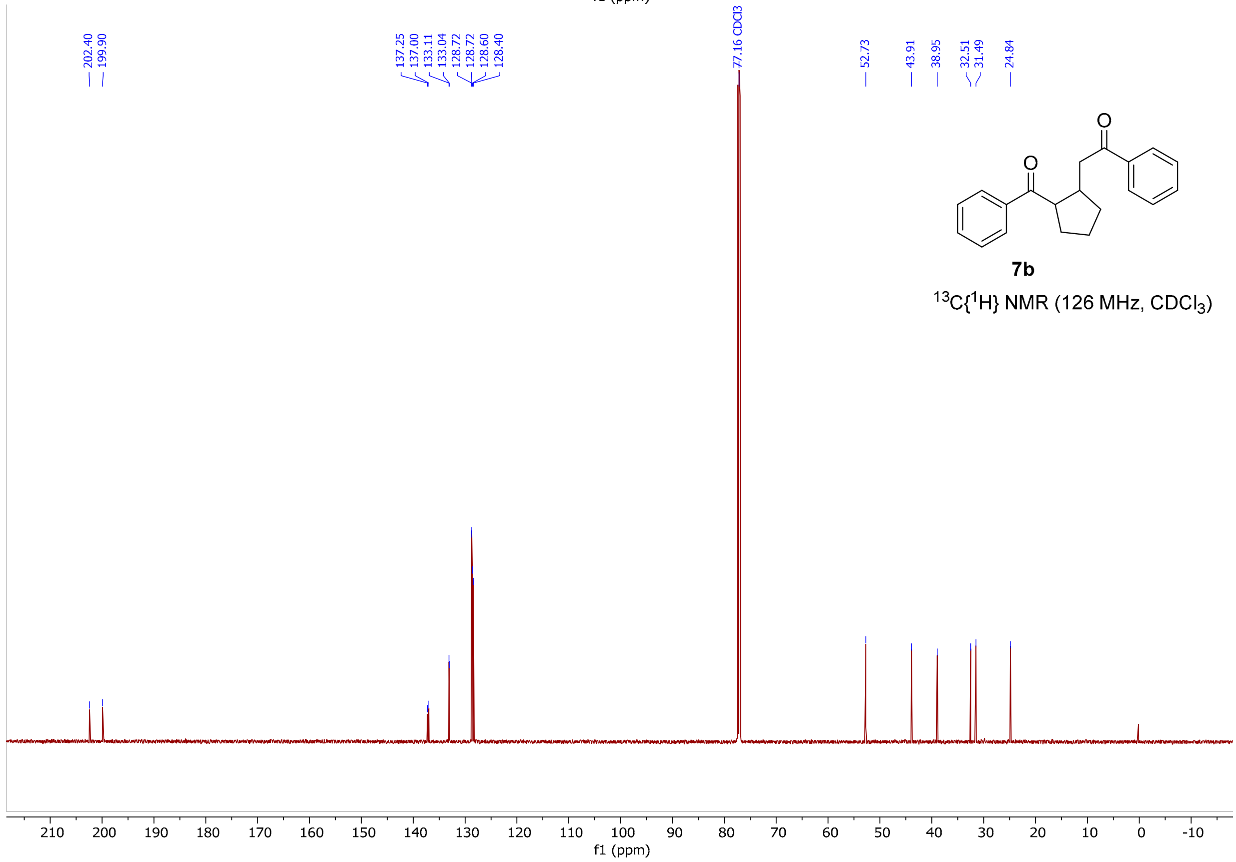
7b
^{13}C{^{1}H} NMR (126 MHz, CDCl$_3$)
f1 (ppm)

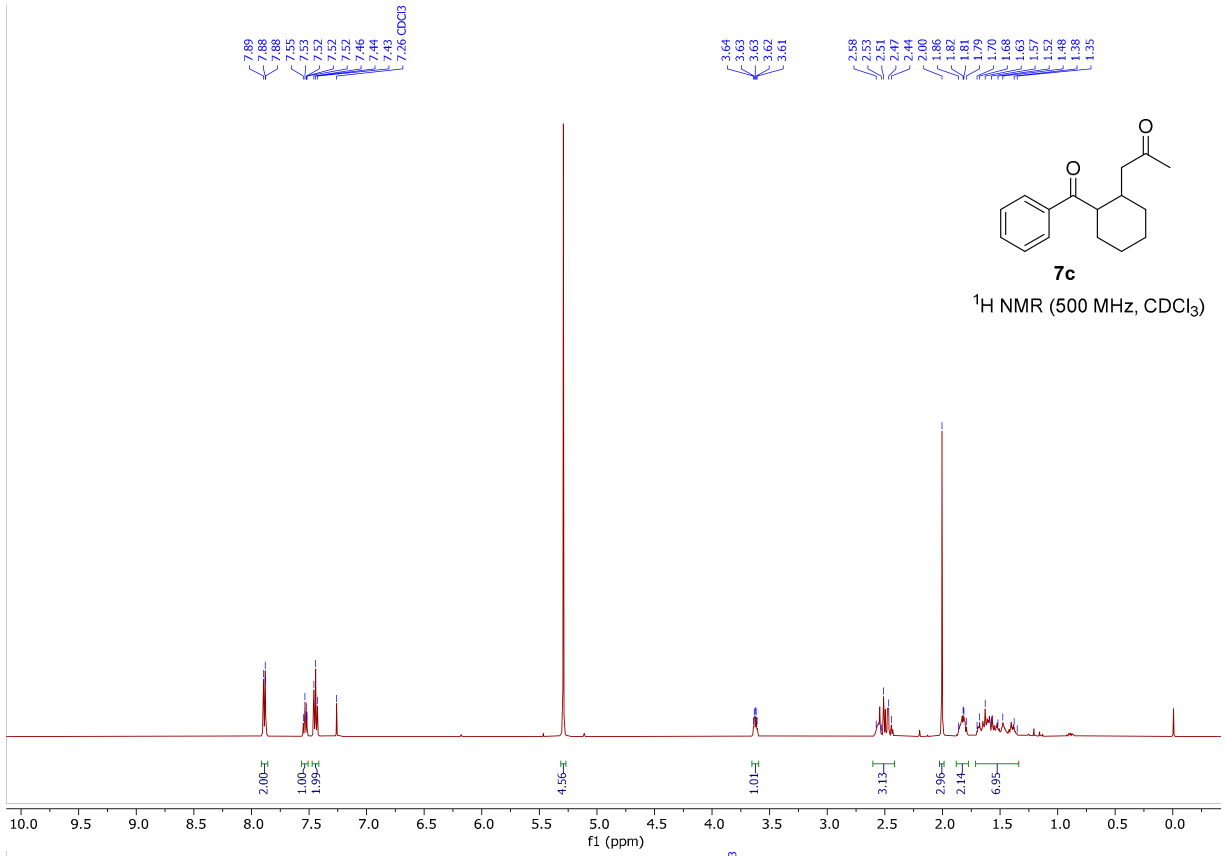
7.89
7.88
7.88
7.55
7.53
7.52
7.52
7.52
7.46
7.44
7.43
7.26 CDCl3
3.64
3.63
3.63
3.62
3.61
2.58
2.53
2.51
2.47
2.44
2.00
1.86
1.82
1.81
1.79
1.70
1.68
1.63
1.57
1.52
1.48
1.38
1.35
O
O
7c
1H NMR (500 MHz, CDCl3)
2.00
1.00
1.99
4.56
1.01
3.13
2.96
2.14
6.95
f1 (ppm)

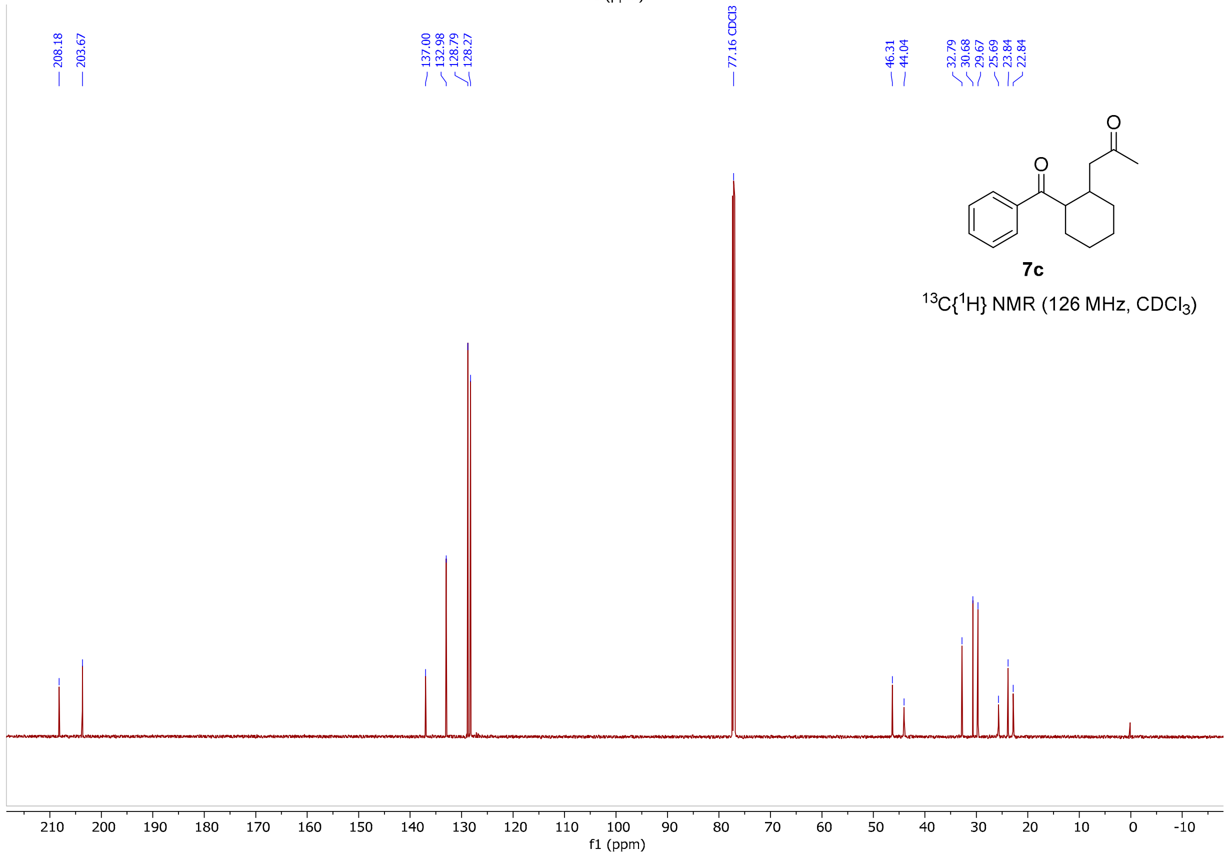
208.18
203.67
137.00
132.98
128.79
128.27
77.16 CDCl3
46.31
44.04
32.79
30.68
29.67
25.69
23.84
22.84
O
O
7c
13C{1H} NMR (126 MHz, CDCl3)
f1 (ppm)

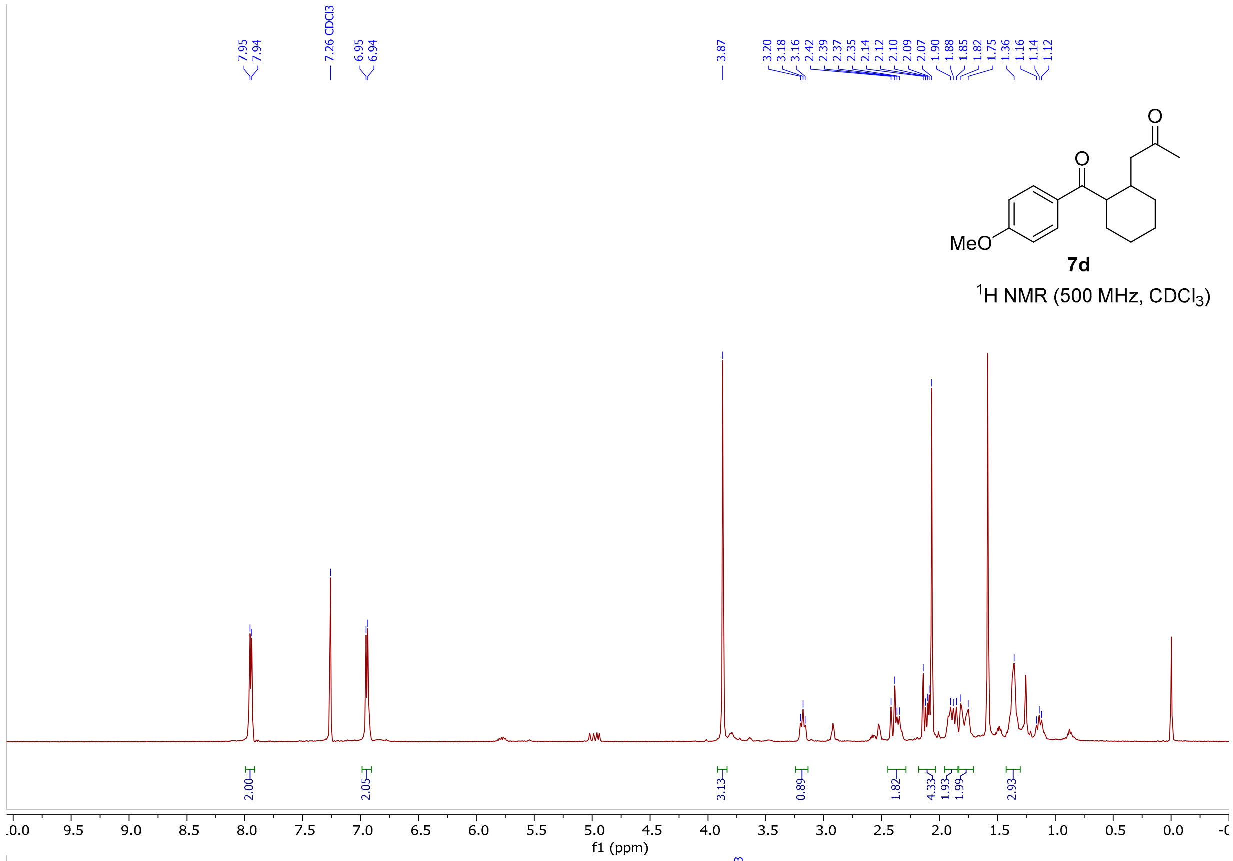
MeO
7d
1H NMR (500 MHz, CDCl3)
f1 (ppm)

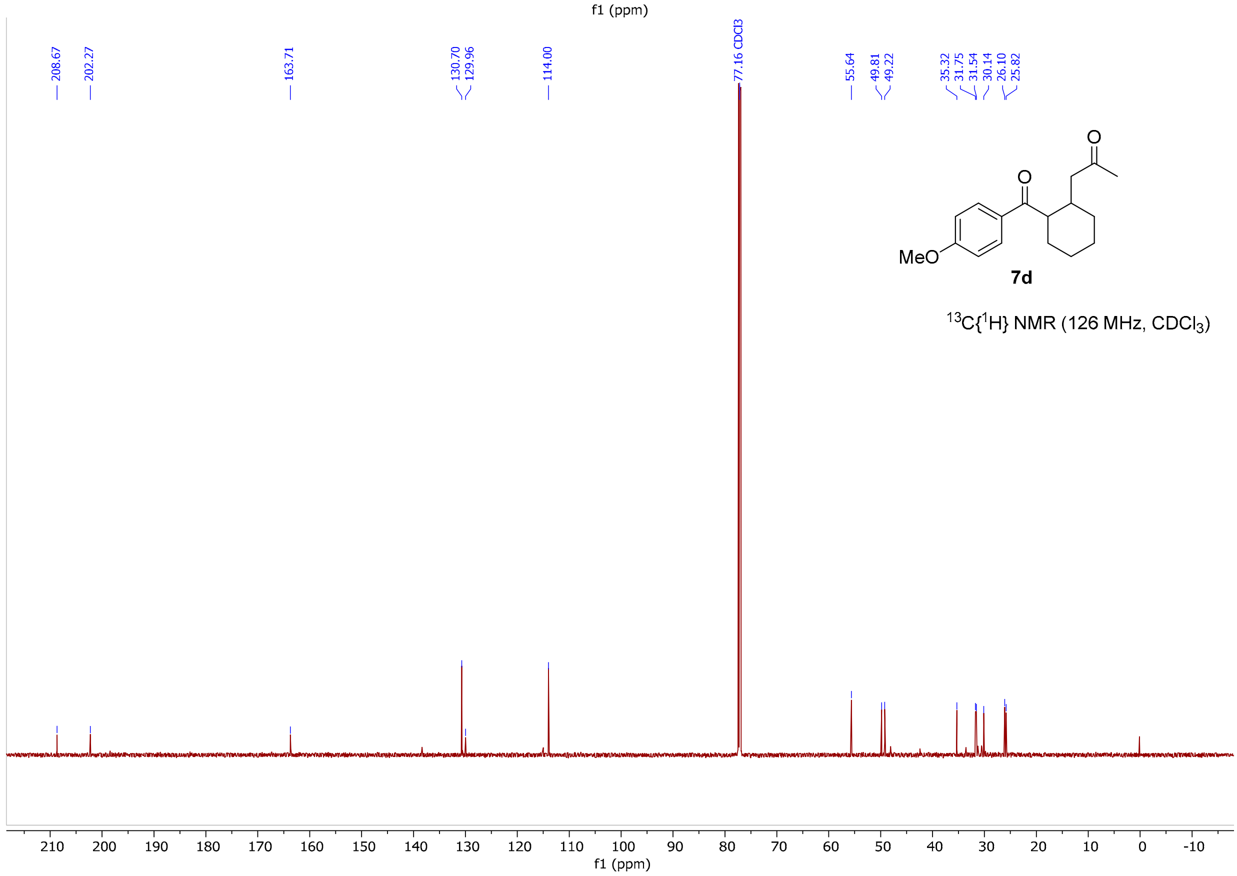
MeO
7d
13C{1H} NMR (126 MHz, CDCl3)
f1 (ppm)

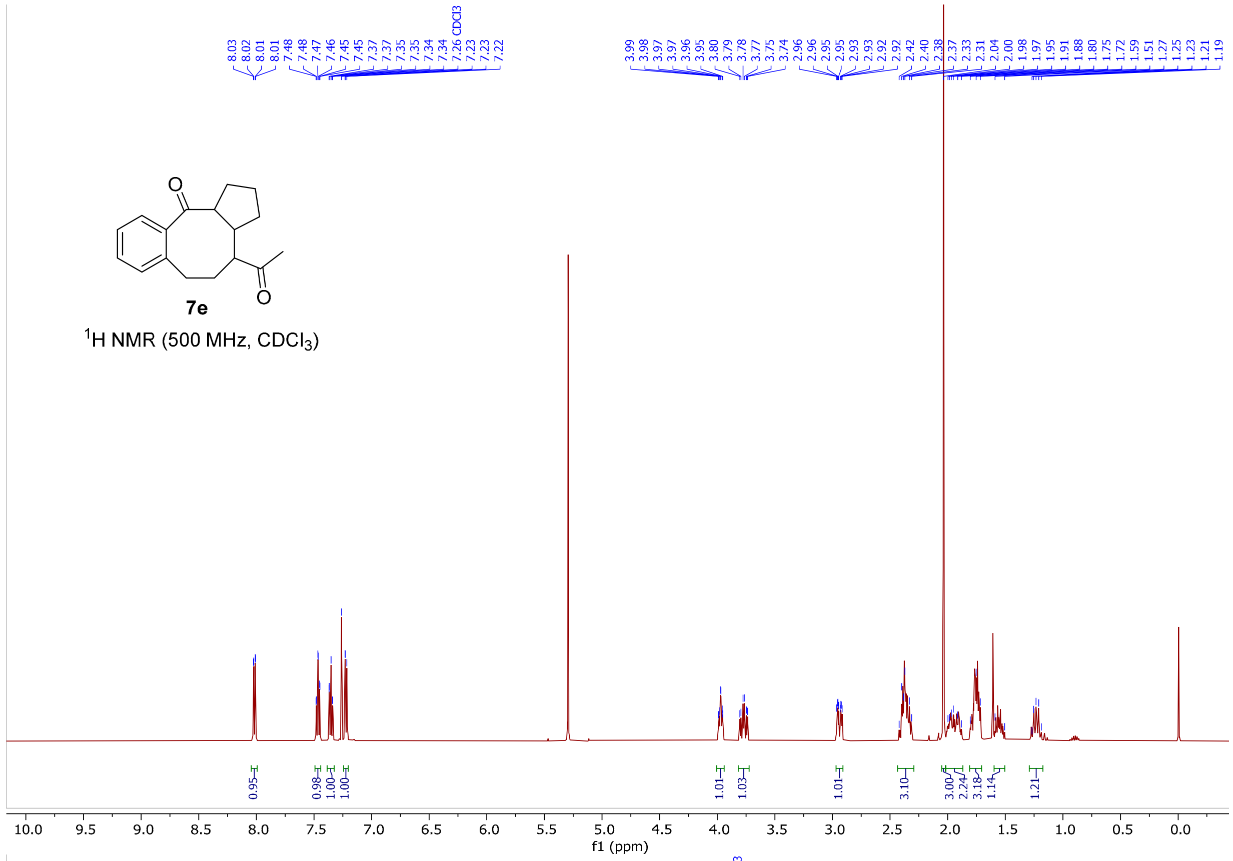
8.03 8.02 8.01 8.01 7.48 7.47 7.46 7.45 7.45 7.37 7.37 7.35 7.35 7.34 7.34 7.26 CDCl3 7.23 7.23 7.22
3.99 3.98 3.97 3.97 3.96 3.95 3.80 3.79 3.78 3.77 3.75 3.74 2.96 2.96 2.95 2.95 2.95 2.93 2.93 2.92 2.92 2.92 2.42 2.40 2.38 2.37 2.33 2.31 2.04 2.00 1.98 1.97 1.95 1.91 1.88 1.80 1.75 1.72 1.59 1.51 1.27 1.25 1.23 1.21 1.19
7e
^{1}H NMR (500 MHz, CDCl$_3$)
0.95 0.98 1.00 1.00 1.01 1.03 1.01 3.10 3.00 2.24 3.18 1.14 1.21
10.0 9.5 9.0 8.5 8.0 7.5 7.0 6.5 6.0 5.5 5.0 4.5 4.0 3.5 3.0 2.5 2.0 1.5 1.0 0.5 0.0
f1 (ppm)

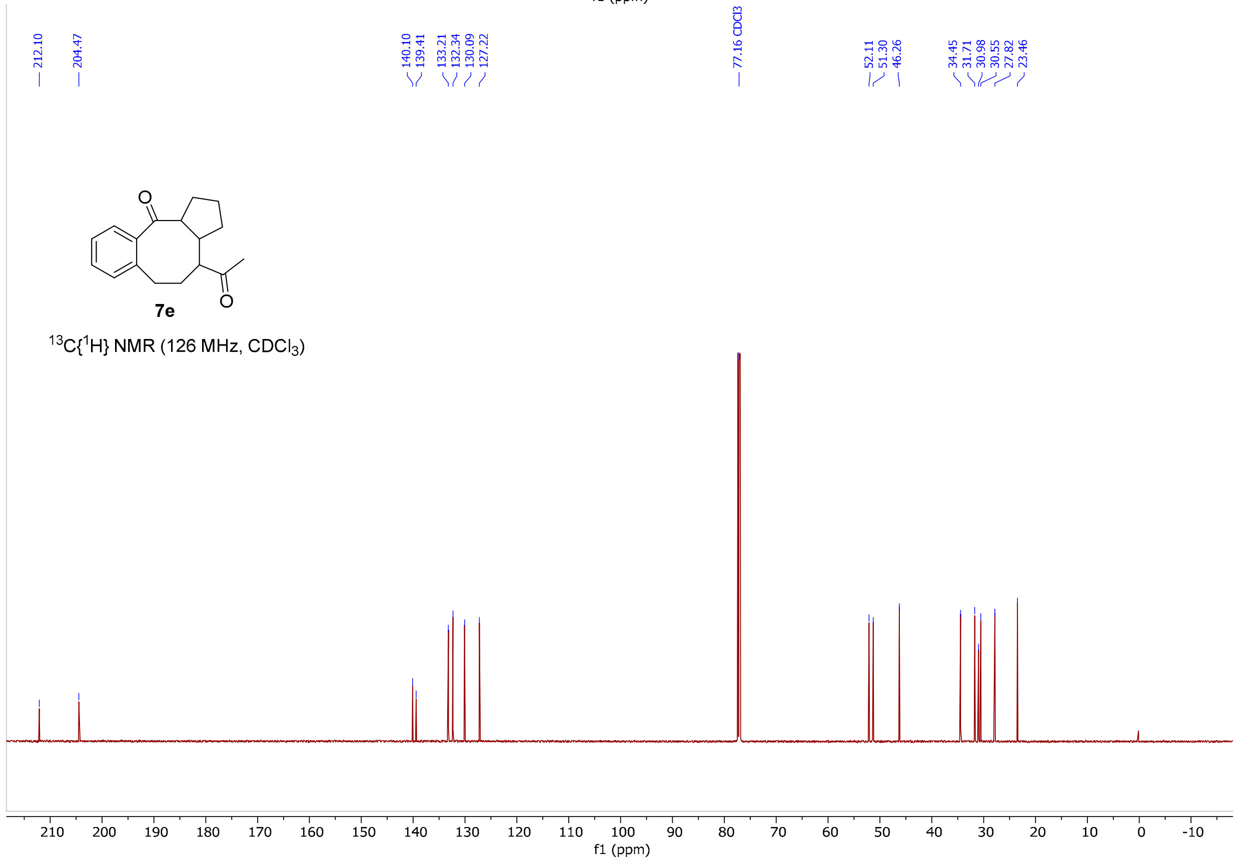
212.10 204.47 140.10 139.41 133.21 132.34 130.09 127.22 77.16 CDCl3 52.11 51.30 46.26 34.45 31.71 30.98 30.55 27.82 23.46
7e
^{13}C{^{1}H} NMR (126 MHz, CDCl$_3$)
210 200 190 180 170 160 150 140 130 120 110 100 90 80 70 60 50 40 30 20 10 0 -10
f1 (ppm)

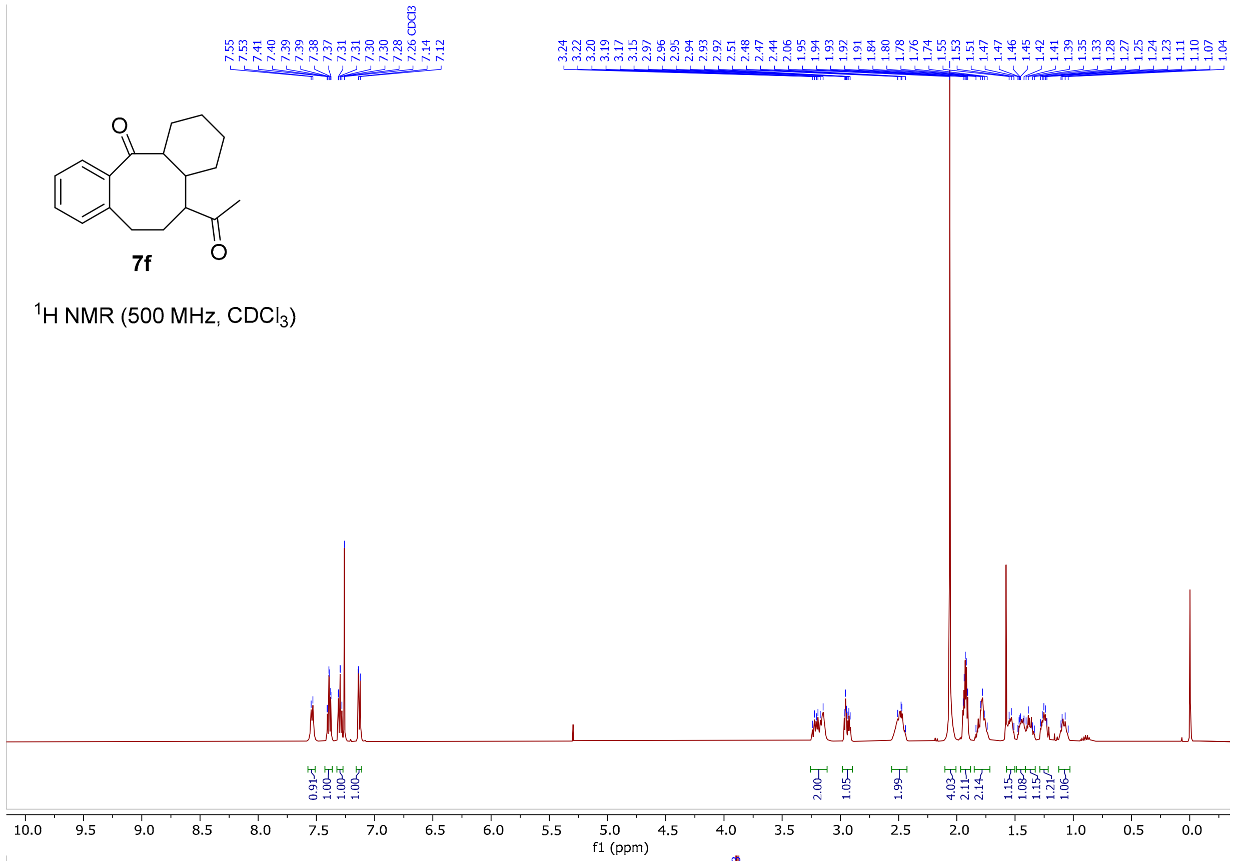
7f
^{1}H NMR (500 MHz, CDCl$_3$)
f1 (ppm)

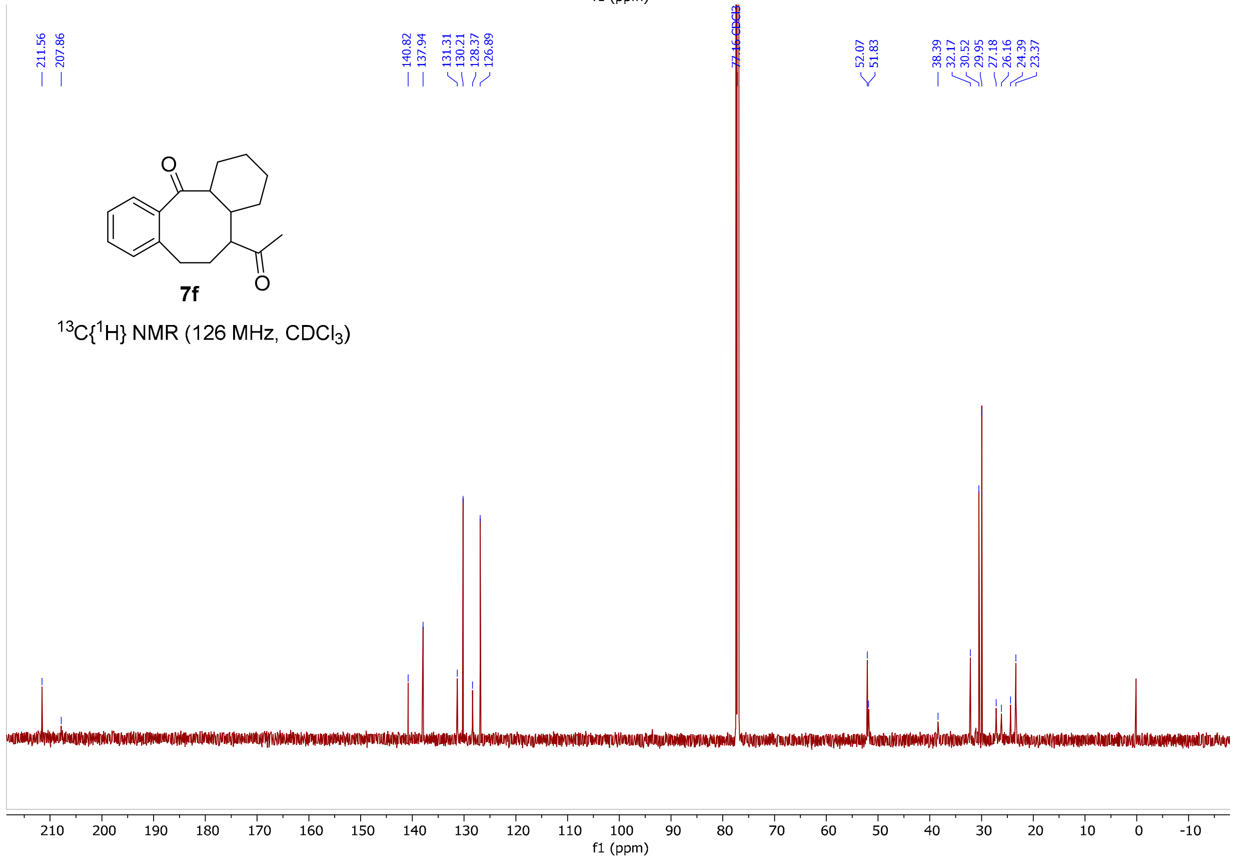
7f
^{13}C{^{1}H} NMR (126 MHz, CDCl$_3$)
f1 (ppm)

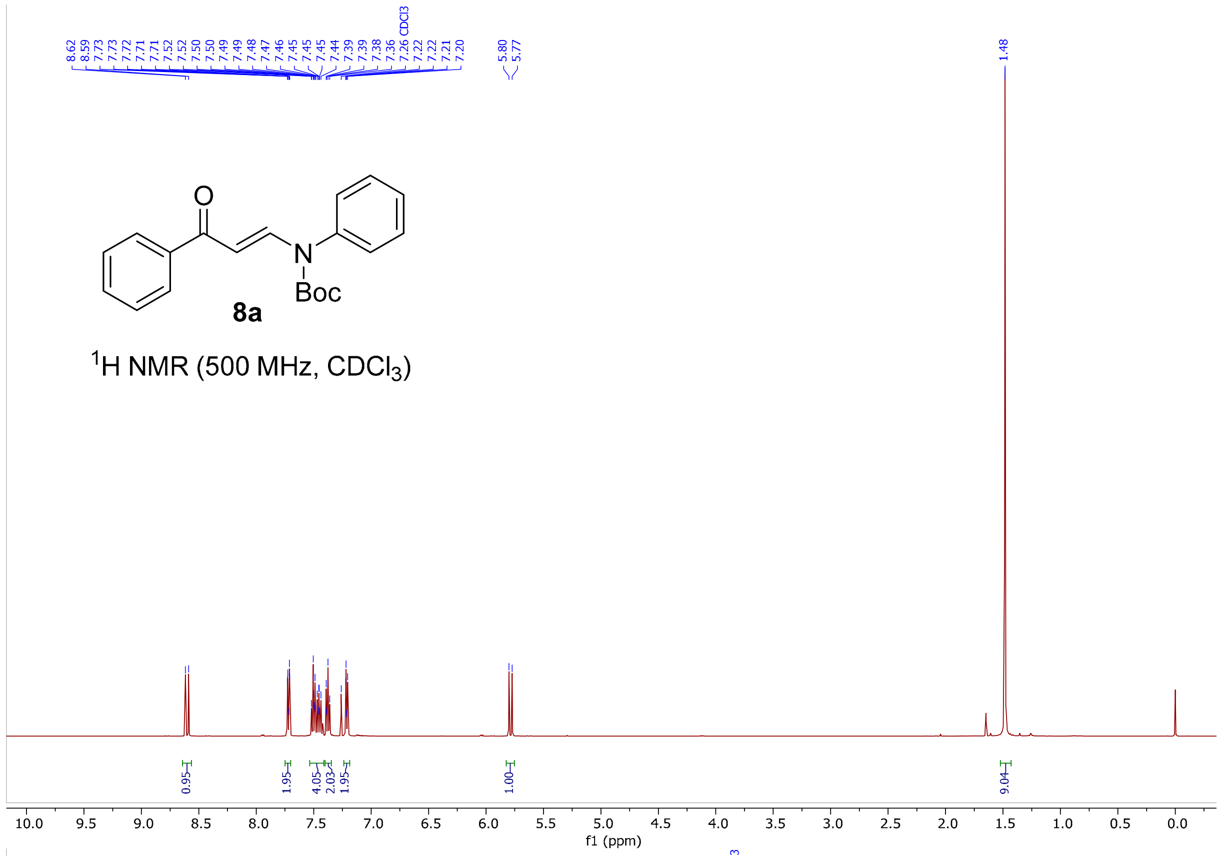
O
N
Boc
8a
1H NMR (500 MHz, CDCl3)
f1 (ppm)

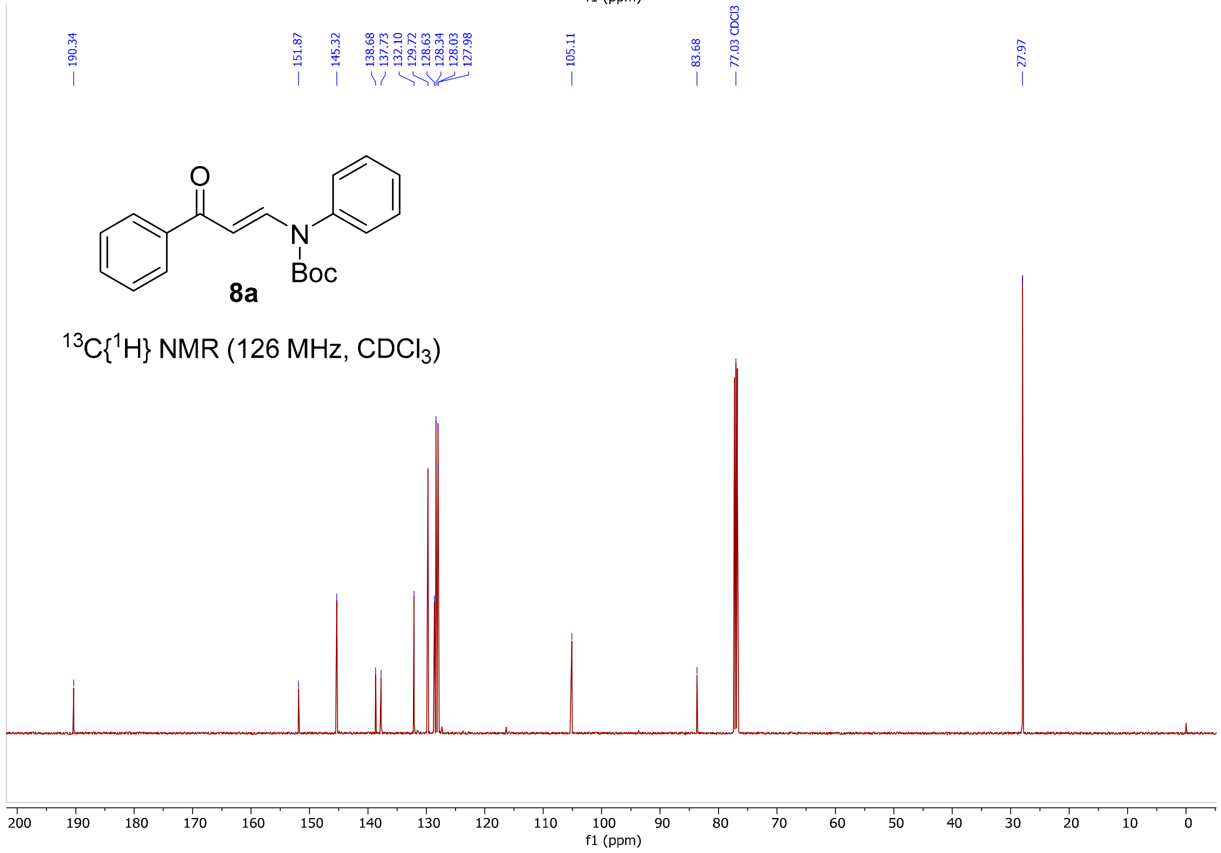
O
N
Boc
8a
13C{1H} NMR (126 MHz, CDCl3)
f1 (ppm)

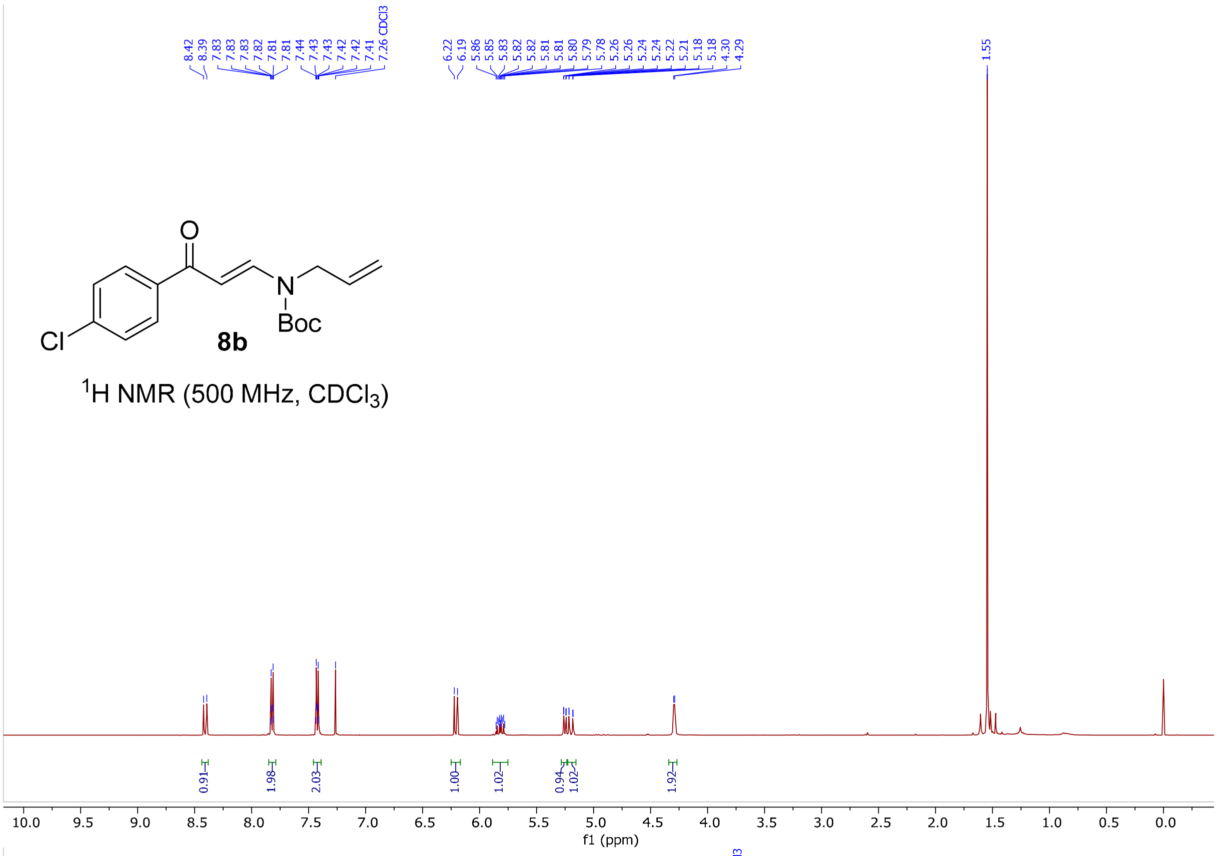
8.42
8.39
7.83
7.83
7.83
7.82
7.81
7.81
7.44
7.43
7.43
7.42
7.42
7.41
7.26 CDCl3
6.22
6.19
5.86
5.85
5.83
5.82
5.82
5.81
5.81
5.80
5.79
5.78
5.26
5.26
5.24
5.24
5.22
5.21
5.18
5.18
4.30
4.29
1.55
O
N
Boc
Cl
8b
1H NMR (500 MHz, CDCl3)
0.91
1.98
2.03
1.00
1.02
0.94
1.02
1.92
f1 (ppm)

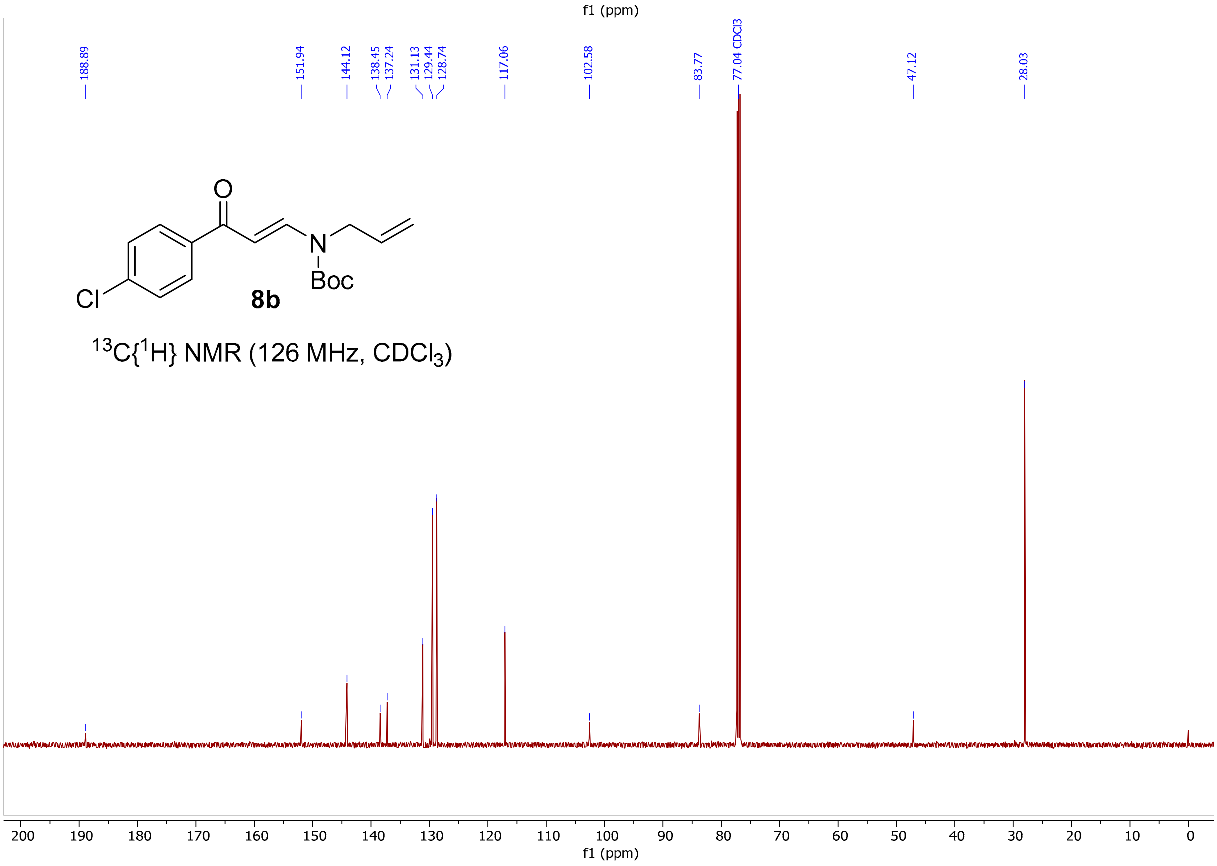
188.89
151.94
144.12
138.45
137.24
131.13
129.44
128.74
117.06
102.58
83.77
77.04 CDCl3
47.12
28.03
O
N
Boc
Cl
8b
13C{1H} NMR (126 MHz, CDCl3)
f1 (ppm)

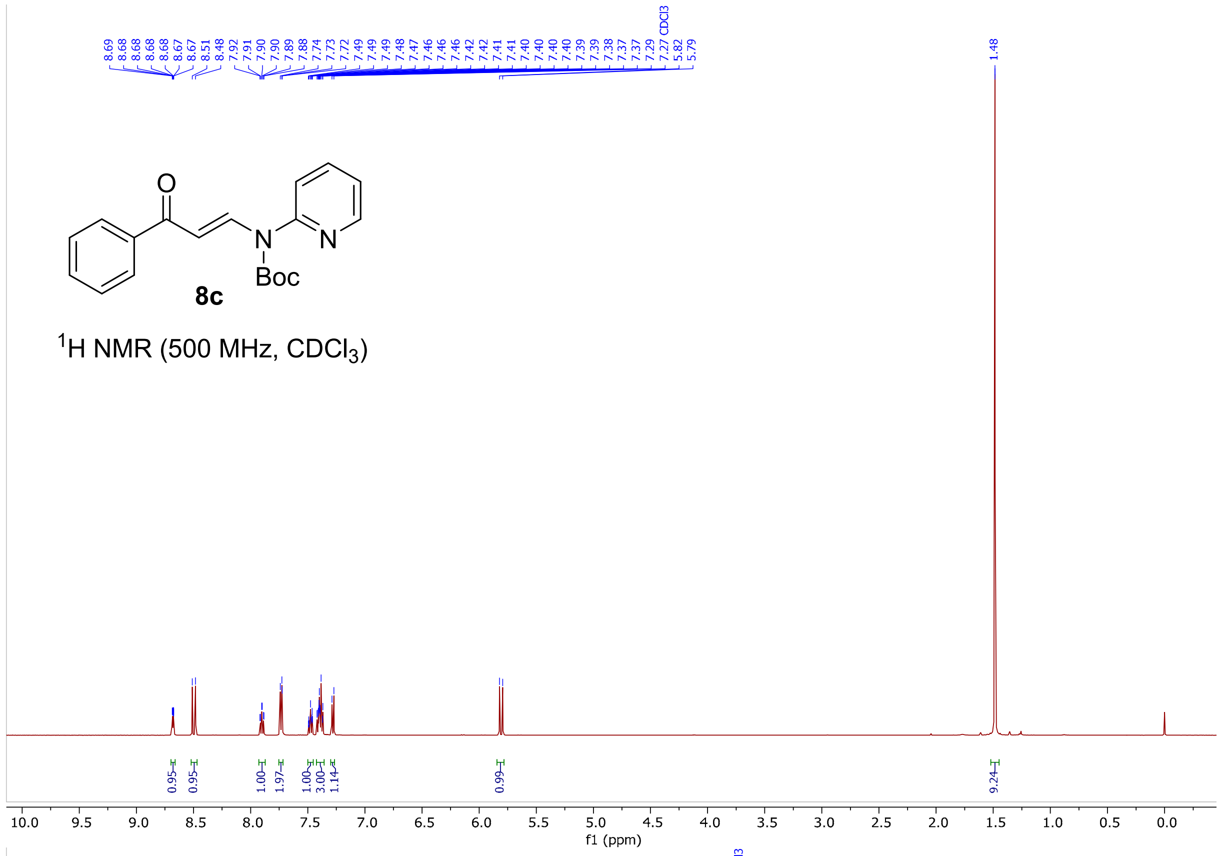

8.69 8.68 8.68 8.68 8.68 8.67 8.67 8.51 8.48 7.92 7.91 7.90 7.90 7.89 7.88 7.74 7.73 7.72 7.49 7.49 7.49 7.48 7.47 7.46 7.46 7.46 7.42 7.42 7.41 7.41 7.40 7.40 7.40 7.40 7.39 7.39 7.38 7.37 7.37 7.29 7.27 CDCl3 5.82 5.79
1.48
O
N
N
Boc
8c
1H NMR (500 MHz, CDCl3)
f1 (ppm)

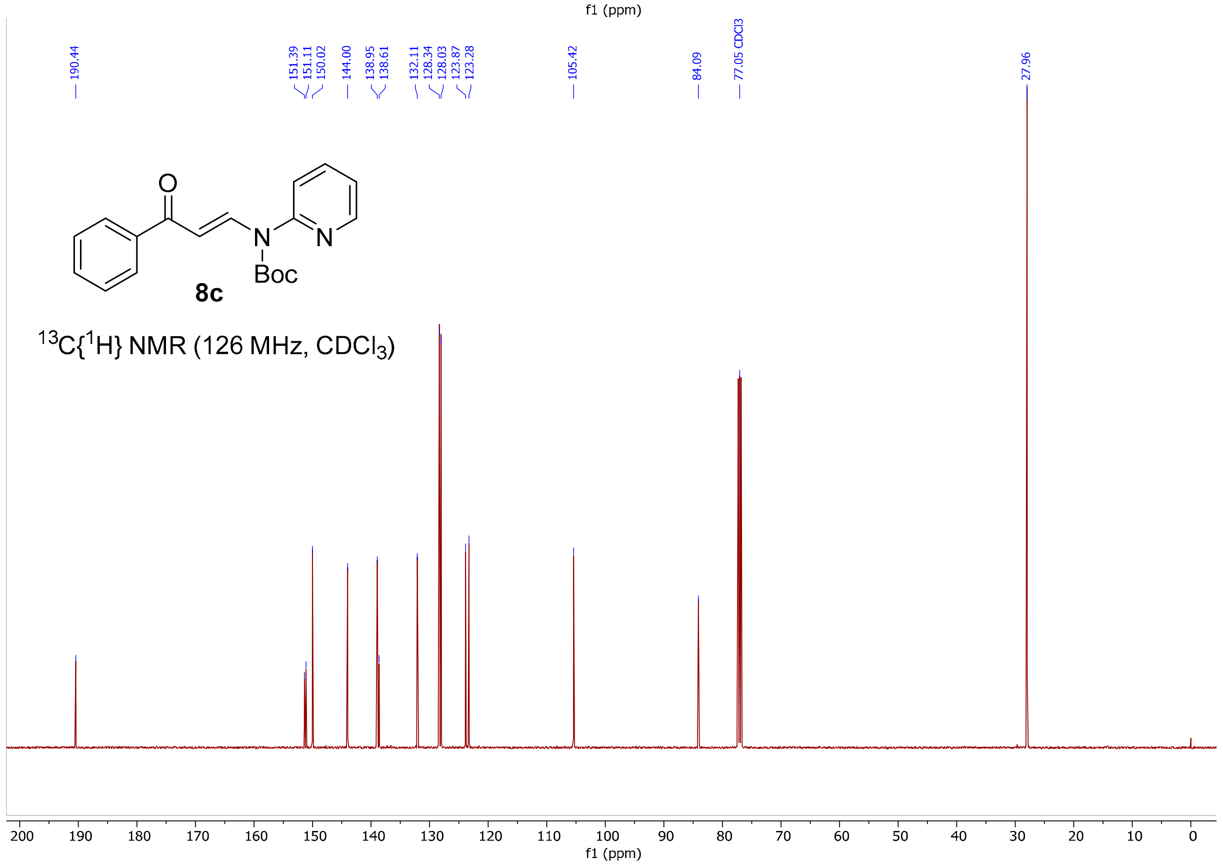

190.44
151.39 151.11 150.02 144.00 138.95 138.61 132.11 128.34 128.03 123.87 123.28
105.42
84.09
77.05 CDCl3
27.96
O
N
N
Boc
8c
13C{1H} NMR (126 MHz, CDCl3)
f1 (ppm)

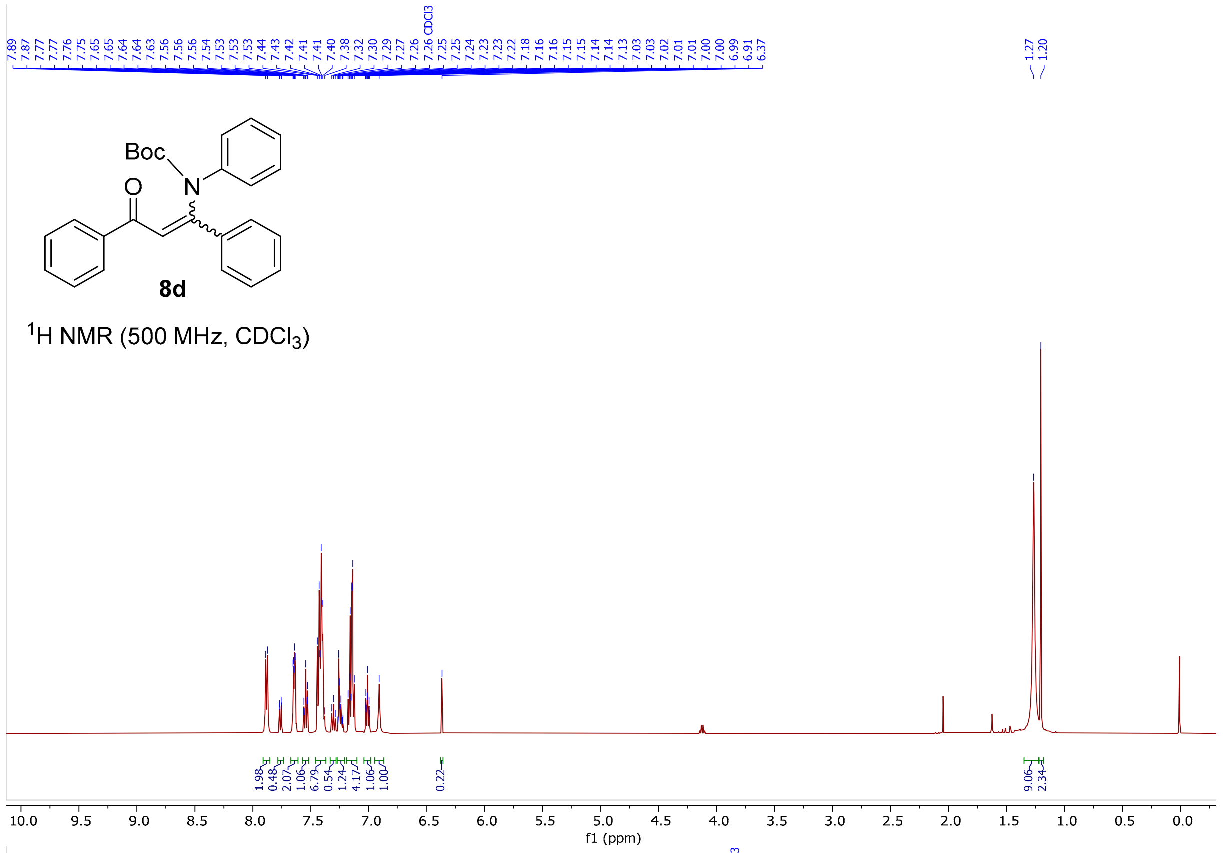

Boc
O
N
8d
^{1}H NMR (500 MHz, CDCl$_3$)
f1 (ppm)

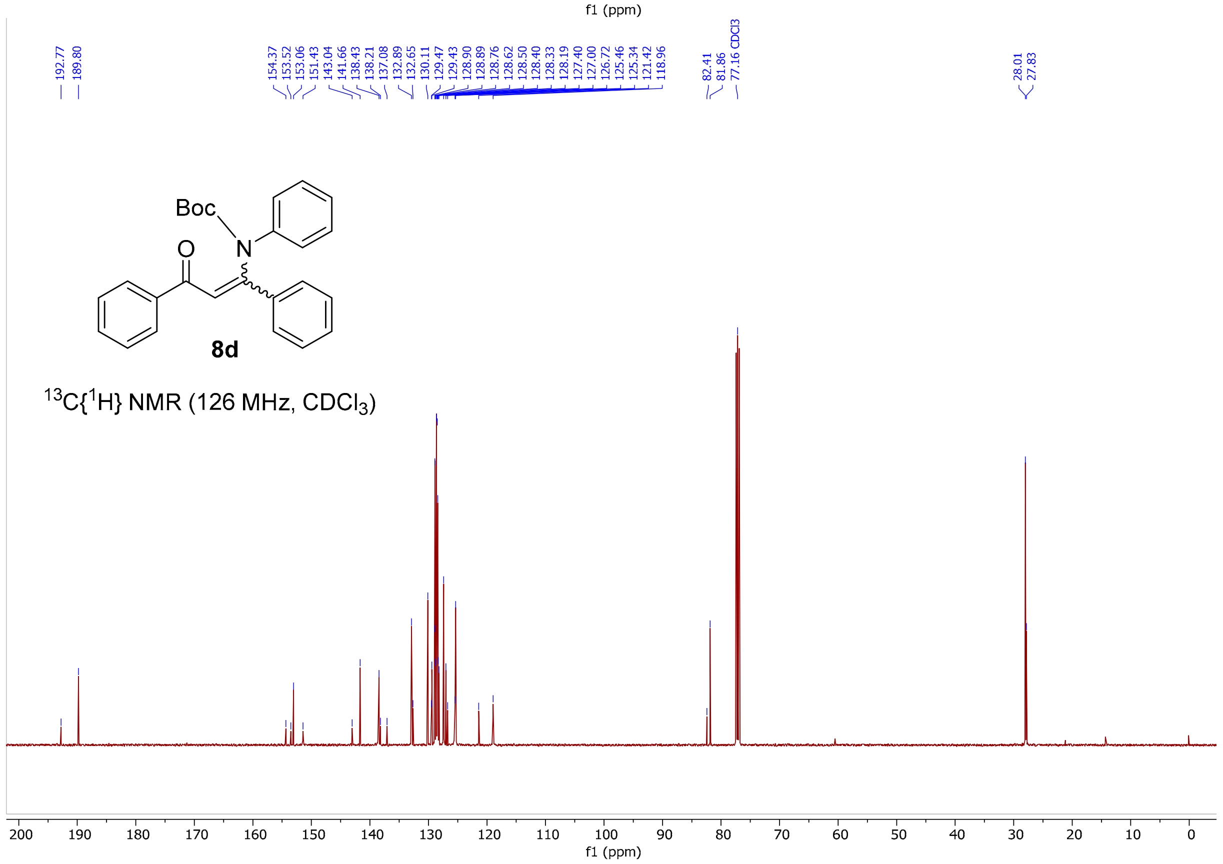

Boc
O
N
8d
^{13}C{^{1}H} NMR (126 MHz, CDCl$_3$)
f1 (ppm)

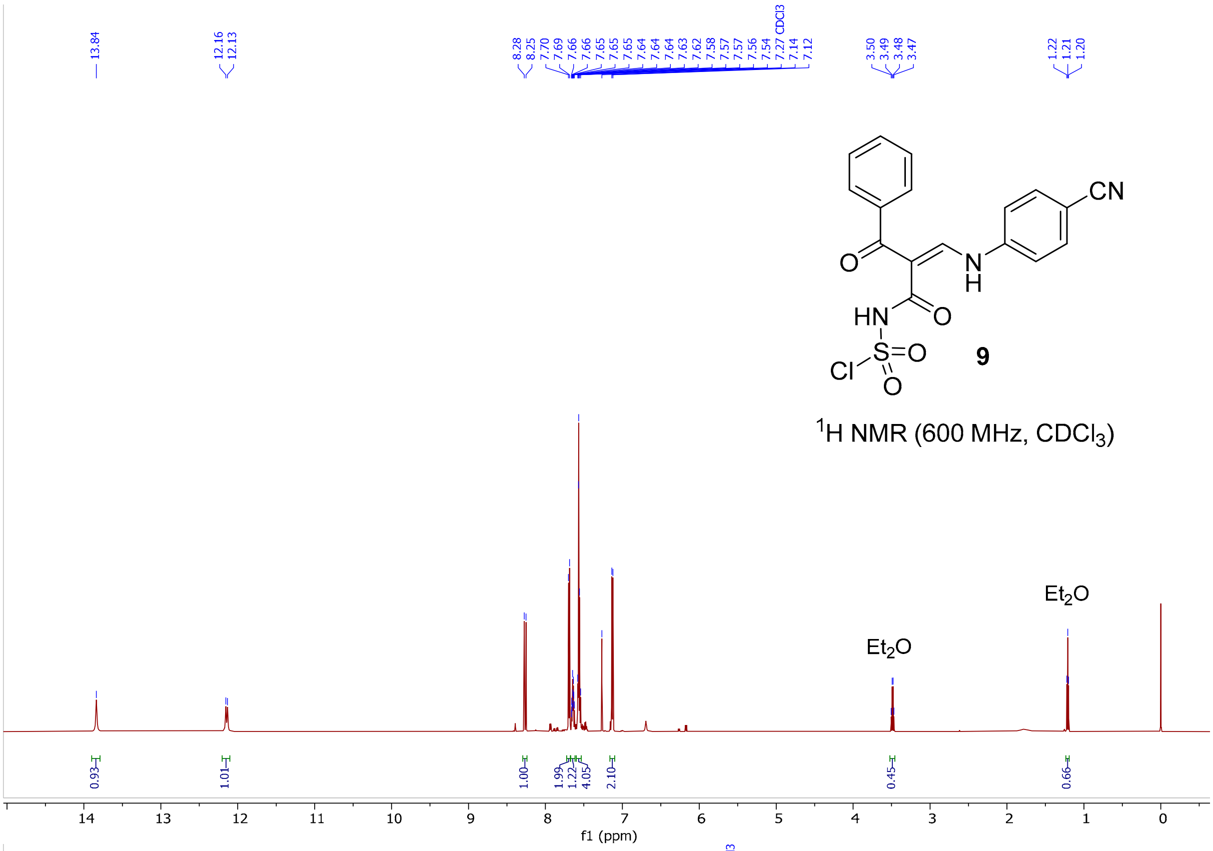
13.84
12.16
12.13
8.28
8.25
7.70
7.69
7.66
7.66
7.65
7.65
7.65
7.64
7.64
7.63
7.62
7.58
7.57
7.57
7.56
7.54
7.27 CDCl3
7.14
7.12
3.50
3.49
3.48
3.47
1.22
1.21
1.20
CN
O
N
H
HN
O
S=O
Cl
O
9
¹H NMR (600 MHz, CDCl₃)
Et₂O
Et₂O
0.93
1.01
1.00
1.99
1.22
4.05
2.10
0.45
0.66
14
13
12
11
10
9
8
7
6
5
4
3
2
1
0
f1 (ppm)

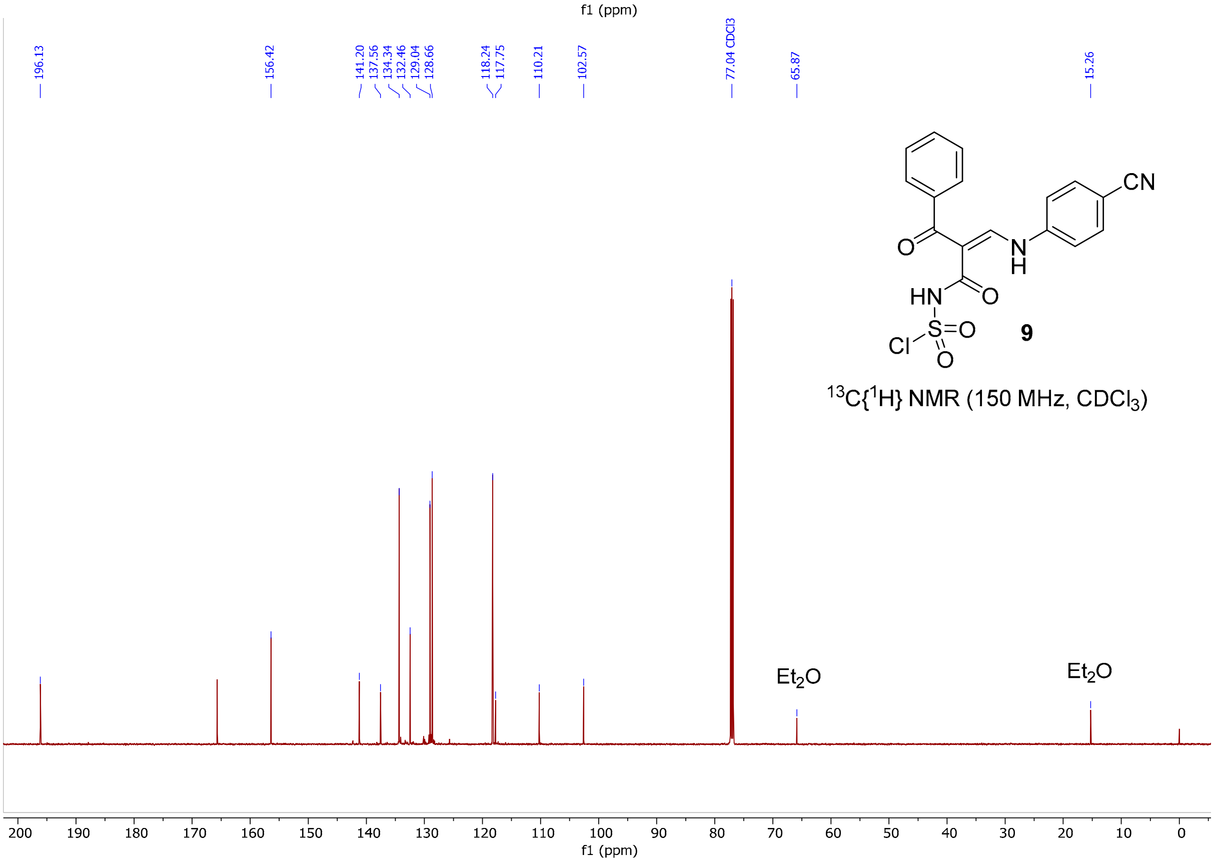
196.13
156.42
141.20
137.56
134.34
132.46
129.04
128.66
118.24
117.75
110.21
102.57
77.04 CDCl3
65.87
15.26
CN
O
N
H
HN
O
S=O
Cl
O
9
¹³C{¹H} NMR (150 MHz, CDCl₃)
Et₂O
Et₂O
200
190
180
170
160
150
140
130
120
110
100
90
80
70
60
50
40
30
20
10
0
f1 (ppm)

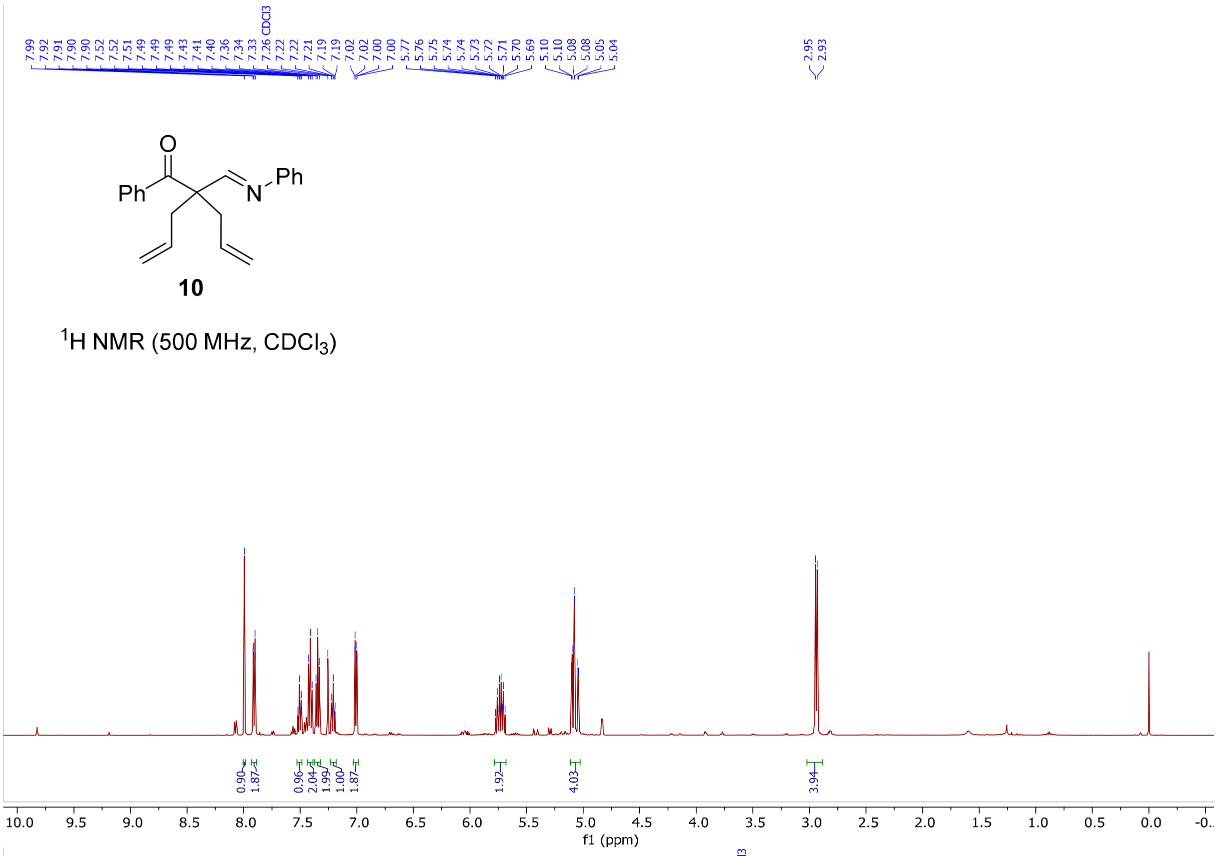
7.99
7.92
7.91
7.90
7.90
7.52
7.52
7.51
7.49
7.49
7.49
7.43
7.41
7.40
7.36
7.34
7.33
7.26 CDCl3
7.22
7.22
7.21
7.19
7.19
7.02
7.02
7.00
7.00
5.77
5.76
5.75
5.74
5.74
5.73
5.72
5.71
5.70
5.69
5.10
5.10
5.08
5.08
5.05
5.04
2.95
2.93
O
Ph
N
Ph
10
^{1}H NMR (500 MHz, CDCl$_3$)
0.90
1.87
0.96
2.04
1.99
1.00
1.87
1.92
4.03
3.94
f1 (ppm)

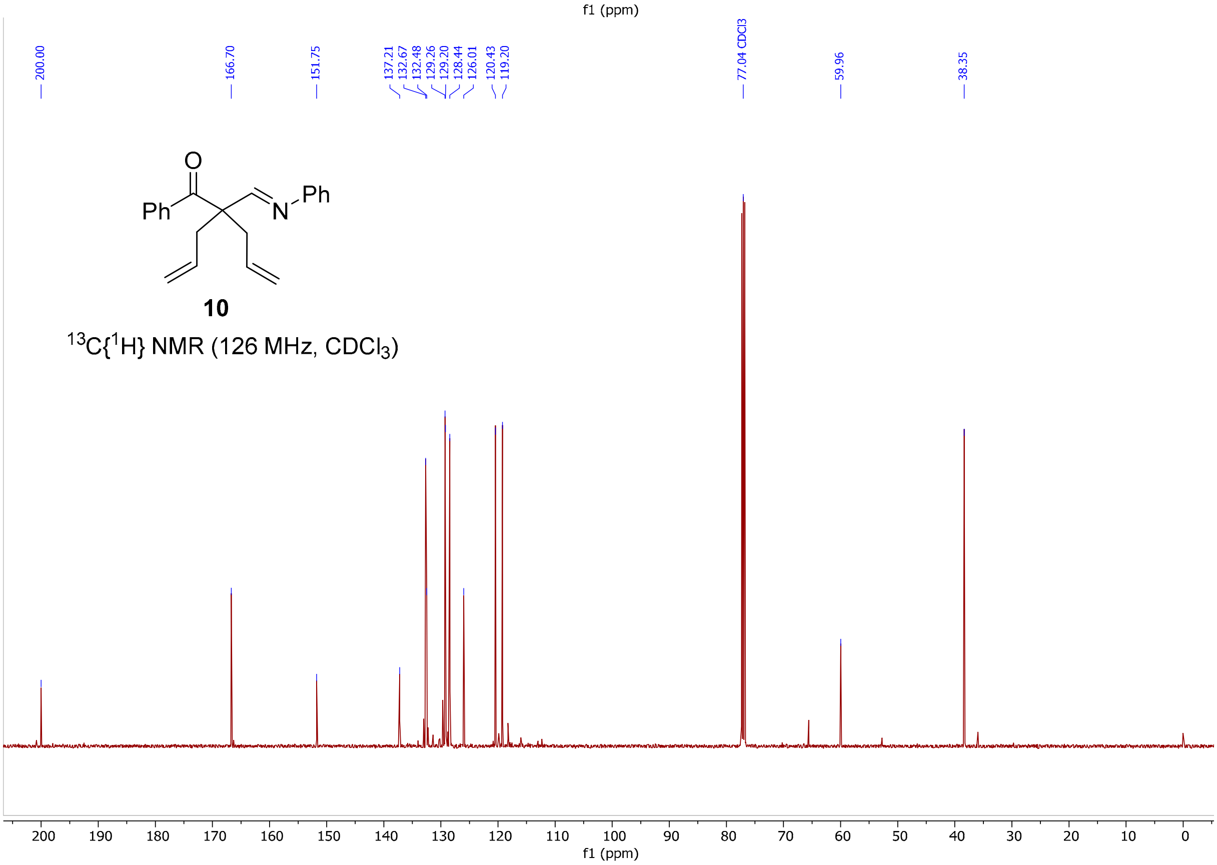
200.00
166.70
151.75
137.21
132.67
132.48
129.26
129.20
128.44
126.01
120.43
119.20
77.04 CDCl3
59.96
38.35
O
Ph
N
Ph
10
^{13}C{^{1}H} NMR (126 MHz, CDCl$_3$)
f1 (ppm)

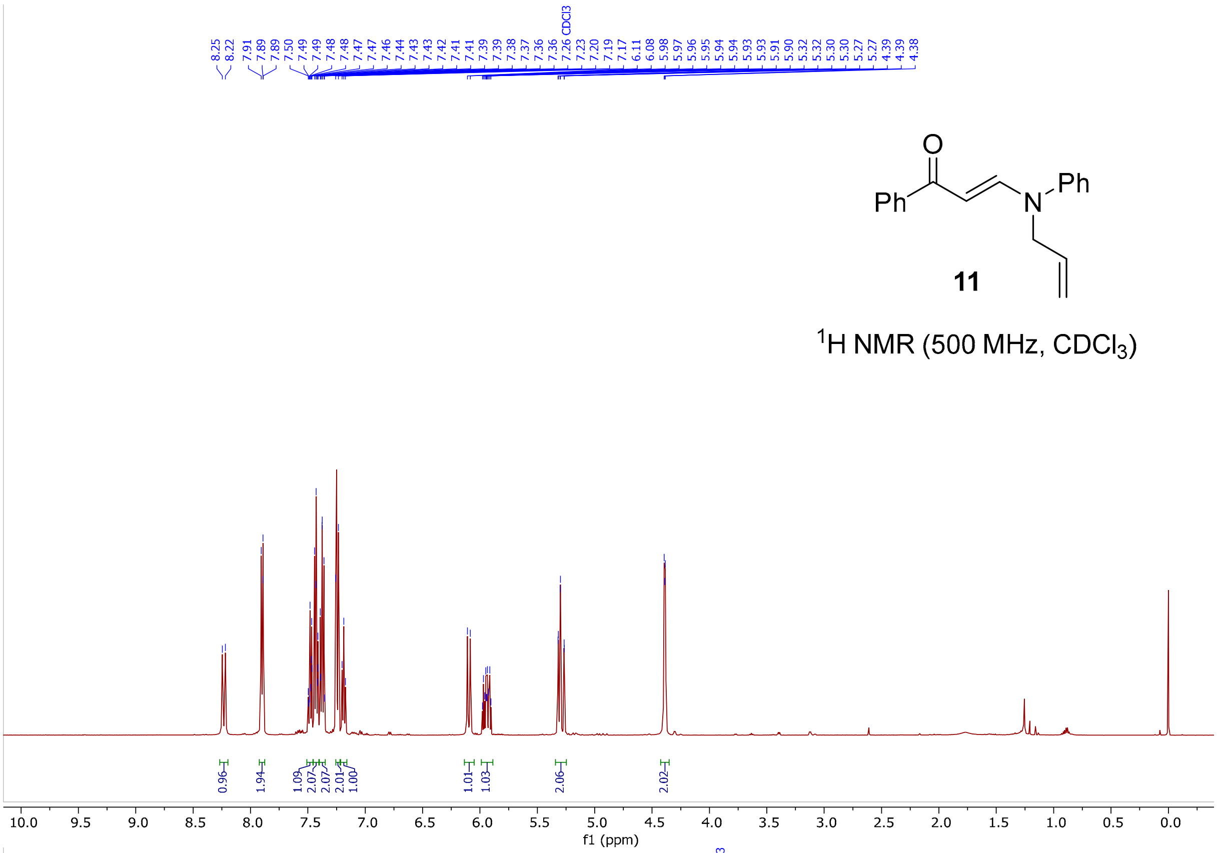
8.25
8.22
7.91
7.89
7.89
7.50
7.49
7.49
7.48
7.48
7.47
7.47
7.46
7.44
7.43
7.43
7.42
7.41
7.41
7.39
7.39
7.38
7.37
7.36
7.26 CDCl3
7.23
7.20
7.19
7.17
6.11
6.08
5.98
5.97
5.96
5.95
5.94
5.94
5.93
5.93
5.91
5.90
5.32
5.32
5.30
5.30
5.27
5.27
4.39
4.39
4.38
O
Ph
N
Ph
11
1H NMR (500 MHz, CDCl3)
0.96
1.94
1.09
2.07
2.07
2.01
1.00
1.01
1.03
2.06
2.02
10.0 9.5 9.0 8.5 8.0 7.5 7.0 6.5 6.0 5.5 5.0 4.5 4.0 3.5 3.0 2.5 2.0 1.5 1.0 0.5 0.0
f1 (ppm)

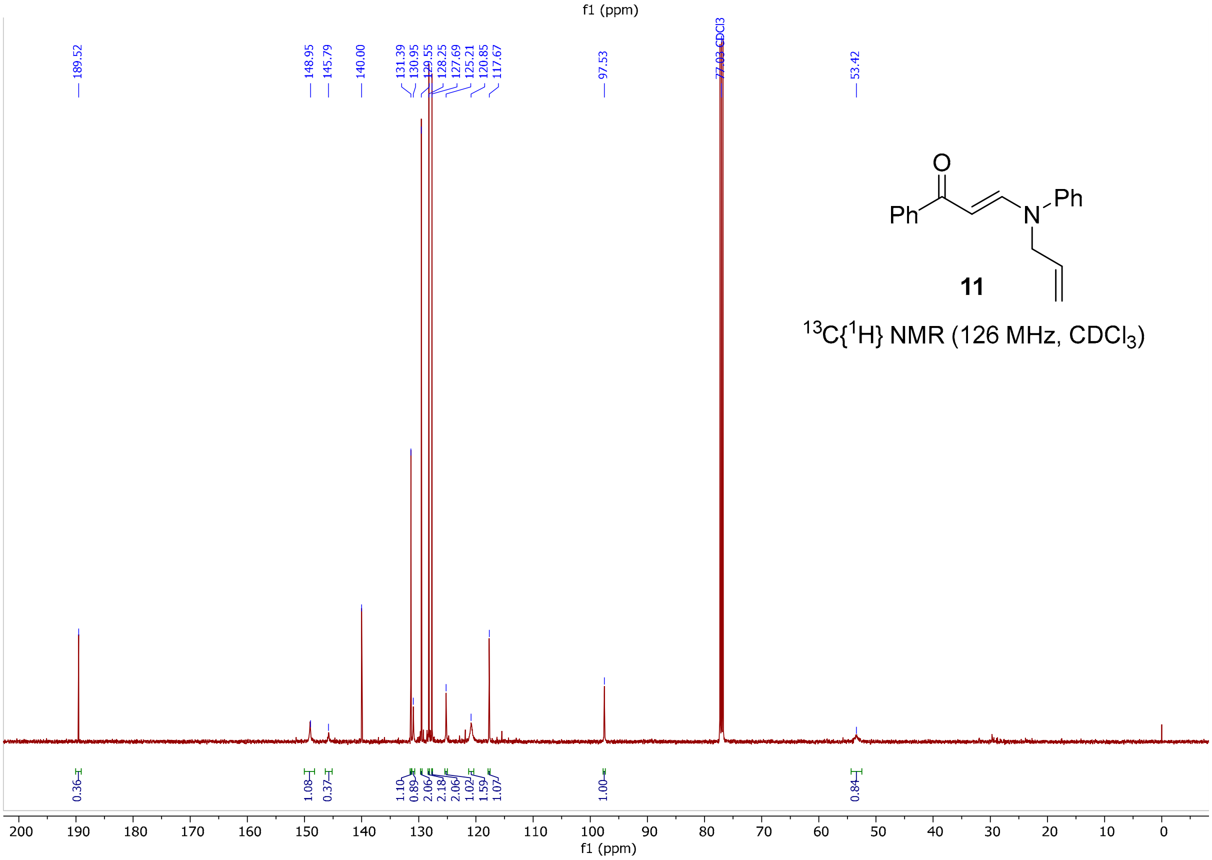
189.52
148.95
145.79
140.00
131.39
130.95
129.55
128.25
127.69
125.21
120.85
117.67
97.53
77.03 CDCl3
53.42
O
Ph
N
Ph
11
13C{1H} NMR (126 MHz, CDCl3)
0.36
1.08
0.37
1.10
0.89
2.06
2.18
2.06
1.02
1.59
1.07
1.00
0.84
200 190 180 170 160 150 140 130 120 110 100 90 80 70 60 50 40 30 20 10 0
f1 (ppm)

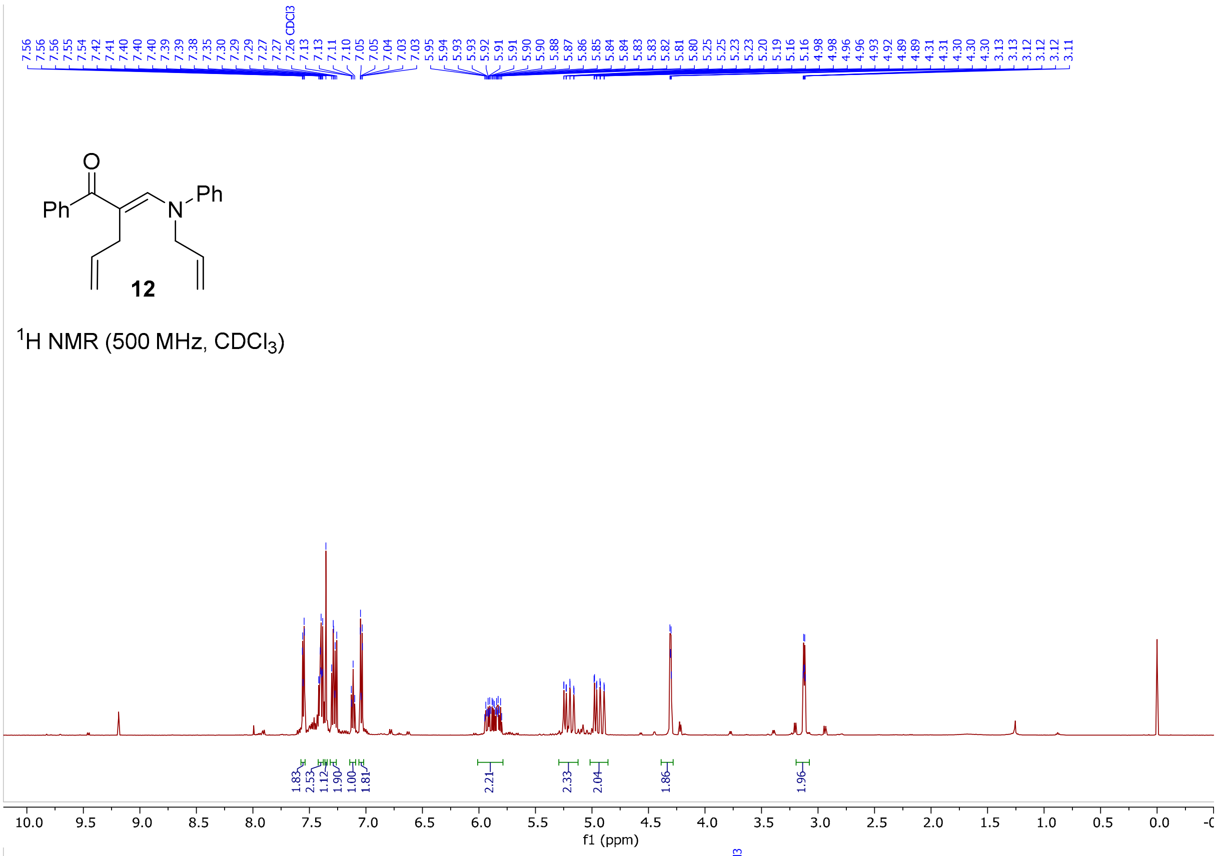

7.56 7.56 7.56 7.55 7.54 7.42 7.41 7.40 7.40 7.40 7.39 7.39 7.38 7.35 7.30 7.29 7.29 7.27 7.27 7.26 CDCl3 7.13 7.13 7.11 7.10 7.05 7.05 7.04 7.03 7.03 5.95 5.94 5.93 5.93 5.92 5.91 5.91 5.90 5.90 5.88 5.87 5.86 5.85 5.84 5.84 5.83 5.83 5.82 5.81 5.80 5.25 5.25 5.23 5.23 5.20 5.19 5.16 5.16 4.98 4.98 4.96 4.96 4.93 4.92 4.89 4.89 4.31 4.31 4.30 4.30 3.13 3.13 3.12 3.12 3.11
O
Ph
N
Ph
12
1H NMR (500 MHz, CDCl3)
1.83 2.53 1.12 1.90 1.00 1.81 2.21 2.33 2.04 1.86 1.96
f1 (ppm)

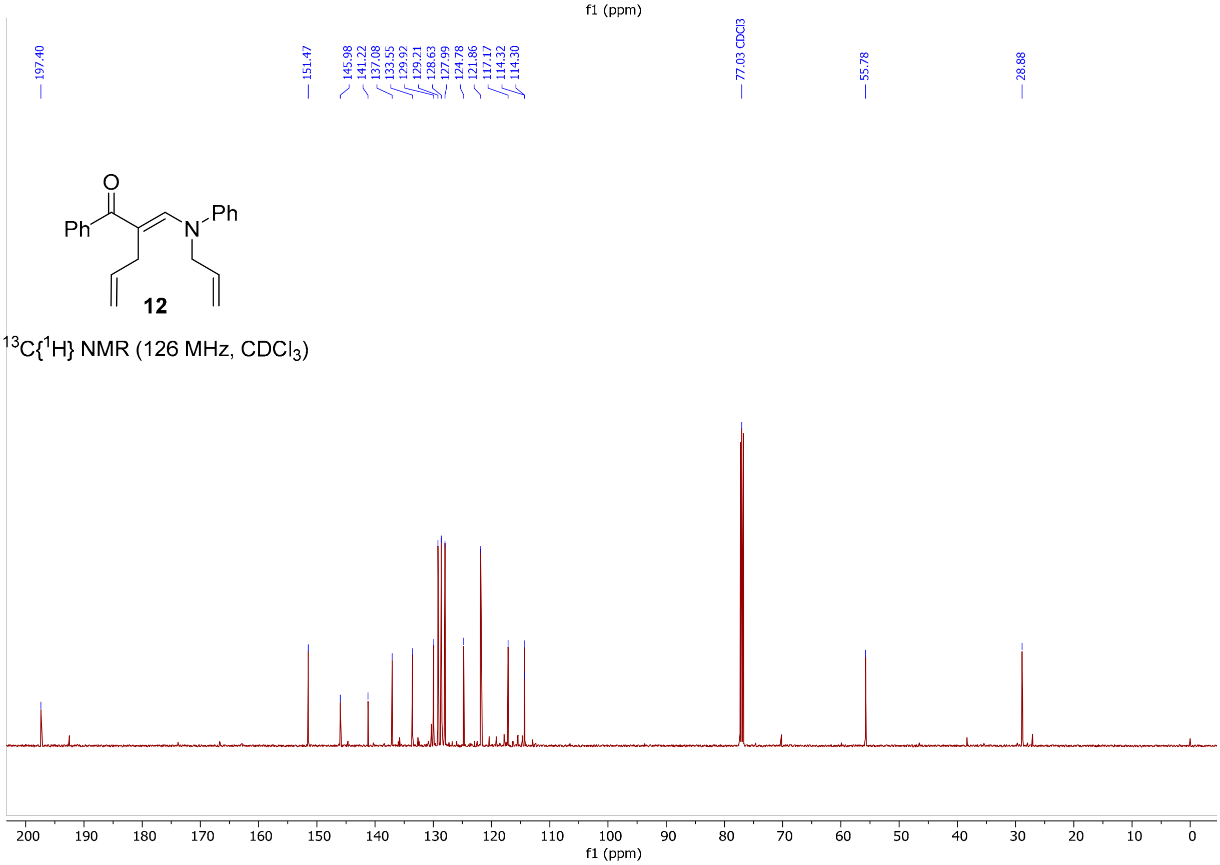

197.40 151.47 145.98 141.22 137.08 133.55 129.92 129.21 128.63 127.99 124.78 121.86 117.17 114.32 114.30 77.03 CDCl3 55.78 28.88
O
Ph
N
Ph
12
13C{1H} NMR (126 MHz, CDCl3)
f1 (ppm)

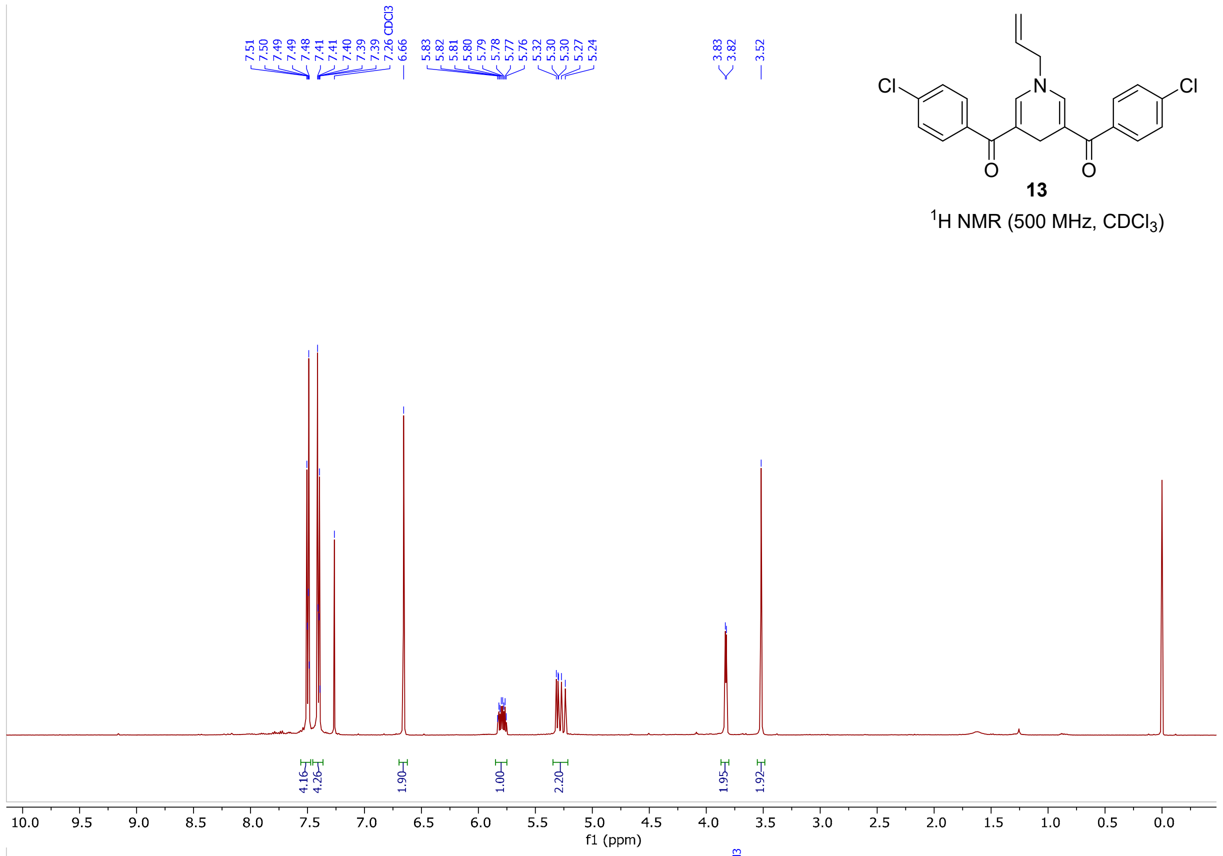
7.51 7.50 7.49 7.49 7.48 7.41 7.41 7.40 7.39 7.39 7.26 CDCl3 6.66
5.83 5.82 5.81 5.80 5.79 5.78 5.77 5.76 5.32 5.30 5.30 5.27 5.24
3.83 3.82 3.52
Cl
N
Cl
O
O
13
^{1}H NMR (500 MHz, CDCl$_3$)
4.16 4.26 1.90 1.00 2.20 1.95 1.92
10.0 9.5 9.0 8.5 8.0 7.5 7.0 6.5 6.0 5.5 5.0 4.5 4.0 3.5 3.0 2.5 2.0 1.5 1.0 0.5 0.0
f1 (ppm)

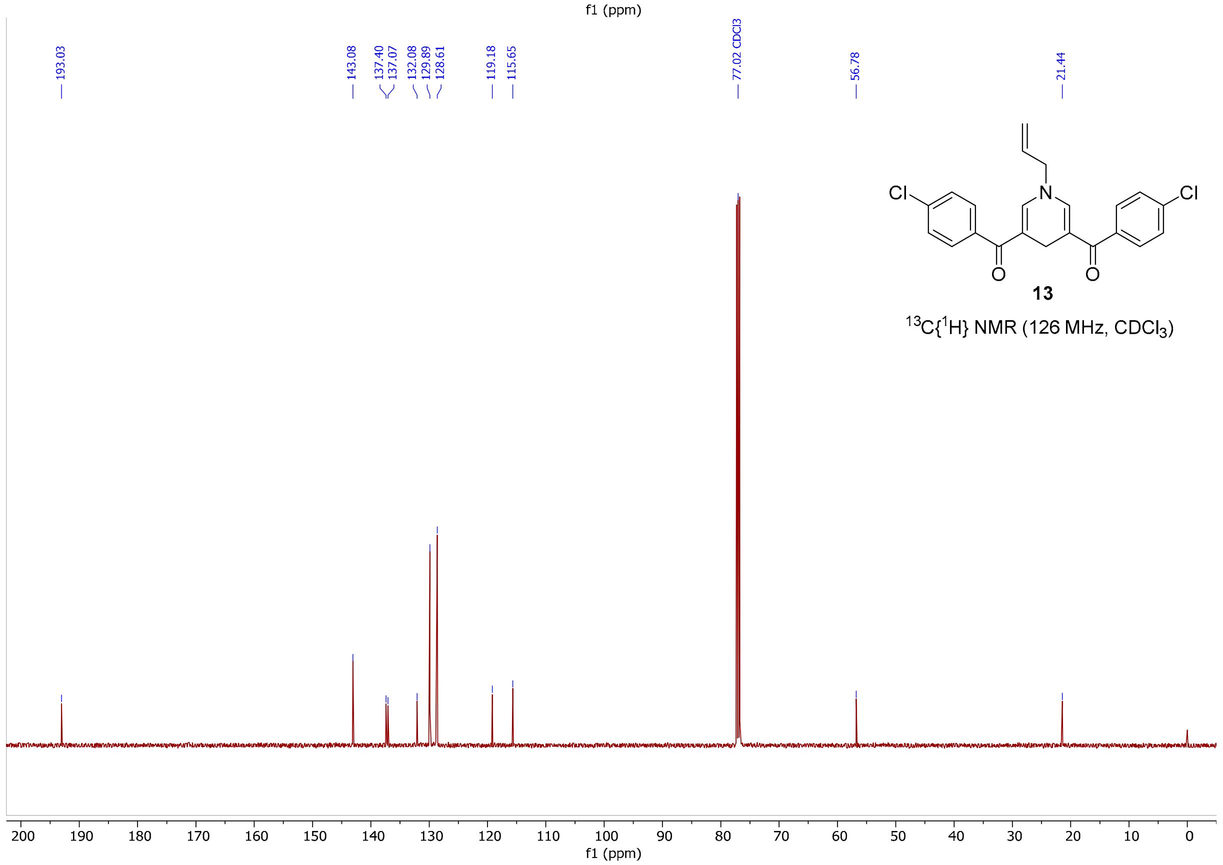
193.03
143.08 137.40 137.07 132.08 129.89 128.61 119.18 115.65
77.02 CDCl3
56.78
21.44
Cl
N
Cl
O
O
13
^{13}C{^{1}H} NMR (126 MHz, CDCl$_3$)
200 190 180 170 160 150 140 130 120 110 100 90 80 70 60 50 40 30 20 10 0
f1 (ppm)

2. X-Ray structure determination for compounds 5b, 8a, 8c, and 8d.

Single-crystal X-ray diffraction data was collected on Agilent Technologies SuperNova Dual diffractometer with an Atlas detector using monochromated Mo-K_{α} radiation (λ = 0.71073 Å) at 150 K. The data was processed using CrysAlis PRO [1]. Using Olex2.1.2. [2], the structures were solved by direct methods implemented in SHELXS [3] or SHELXT [4] and refined by a full-matrix least-squares procedure based on F^2 with SHELXT-2014/7 [5]. All nonhydrogen atoms were refined anisotropicallly. Hydrogen atoms were placed in geometrically calculated positions and were refined using a riding model. The drawings and the analysis of bond lengths, angles and intermolecular interactions were carried out using Mercury [6] and Platon [7].

Table S1. Crystal data and structure refinement for **5b**, **8a**, **8c**, and **8d**.

Compound	**5b**	**8a**	**8c**	**8d**
Empirical formula	$C_{15}H_{12}BF_2NO$	$C_{20}H_{21}NO_3$	$C_{19}H_{20}N_2O_3$	$C_{26}H_{25}NO_3$
Formula weight	271.07	323.38	324.37	399.47
Temperature/K	153(6)	150.00(10)	149.4(8)	150.00(10)
Crystal system	Monoclinic	Monoclinic	Orthorhombic	Orthorhombic
Space group	P21/c	$P2_1/n$	$Pca2_1$	$Pca2_1$
a [Å^3]	15.0139(13)	10.6591(4)	10.3763(5)	16.2283(4)
b [Å^3]	6.0913(4)	9.9707(4)	9.3599(5)	13.6618(3)
c [Å^3]	14.5275(13)	17.1028(8)	17.7620(8)	19.5121(4)
α [°]	90	90	90	90
β [°]	107.752(9)	96.576(4)	90	90
γ [°]	90	90	90	90
V [Å^3]	1265.34(19)	1805.70(13)	1725.07(15)	4325.98(17)
Z	4	4	4	8
ρ_{calc} [g/cm^3]	1.423	1.190	1.249	1.227
μ [mm^{-1}]	0.108	0.080	0.085	0.080
F(000)	560.0	688.0	688.0	1696.0
Crystal size/mm^3	0.4 × 0.2 × 0.2	0.2 × 0.2 × 0.1	0.3 × 0.2 × 0.2	0.5 × 0.4 × 0.3
Radiation	MoKα (λ = 0.71073)	MoKα (λ = 0.71073)	MoKα (λ = 0.71073)	MoKα (λ = 0.71073)
Reflections collected	13872	15500	13770	46065
Independent reflections	3485	4480	3722	10743
R_{int}	0.1115	0.0301	0.0339	0.0283
Data/restraints/parameters	3485/0/181	4480/0/221	3722/1/220	10743/1/548
GOF	1.031	1.040	1.069	1.052
R_1, wR_2 [I>=2σ (I)]	0.0555, 0.1356	0.0414, 0.0977	0.0389, 0.0943	0.0351, 0.0773
R_1, wR_2 (all data)	0.0950, 0.1668	0.0569, 0.1072	0.0480, 0.1002	0.0434, 0.0827
$(\Delta\rho)_{max}$ [e Å^{-3}]	0.28	0.05	0.17	0.18
$(\Delta\rho)_{min}$ [e Å^{-3}]	-0.30	-0.18	-0.20	-0.15
CCDC number	2393729	2393617	2393618	2393619

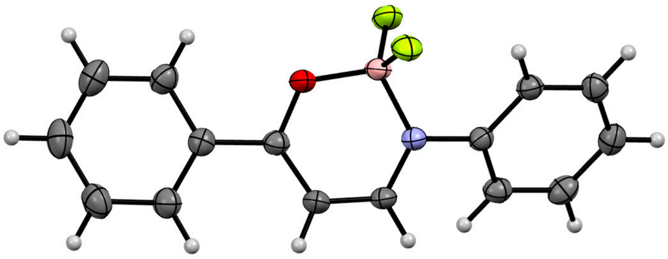

Figure S2. X-Ray structure of compound **5b**.

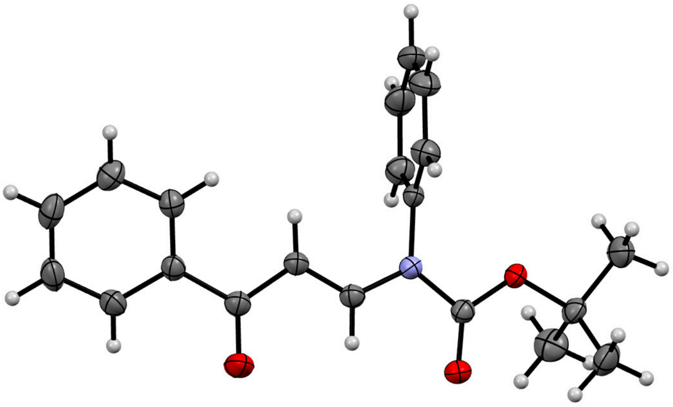

Figure S3. X-Ray structure of compound **8a**.

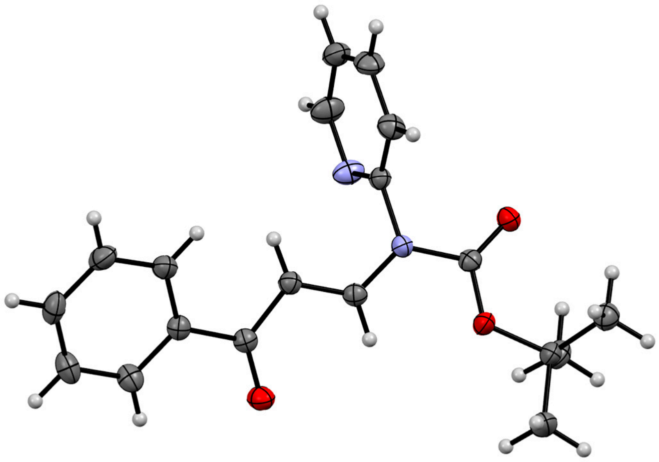

Figure S4. X-Ray structure of compound **8c**.

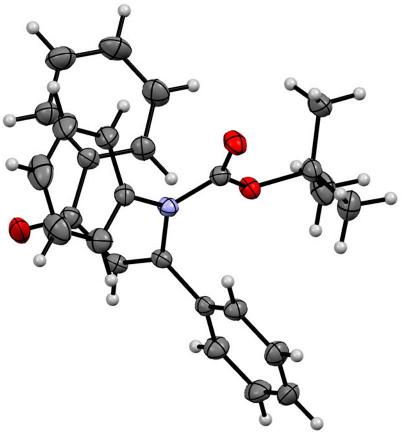

Figure S5. X-Ray structure of compound **8d**.

4. Absorption spectra of compounds 4, 5, 8 and 9.

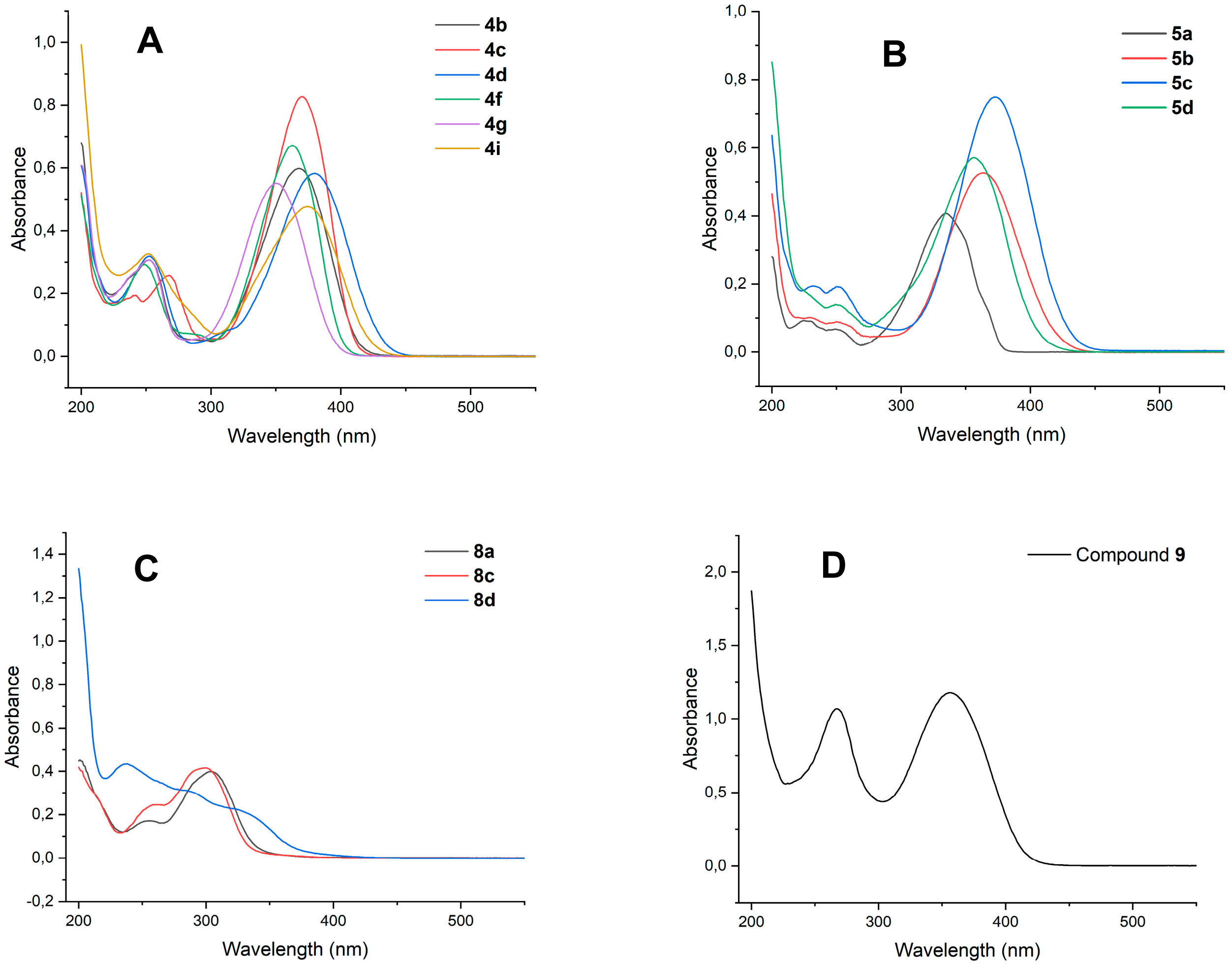

Compound	λmax (nm)	Max. λ of absorption (nm)	Compound	λmax (nm)	Max. λ of absorption (nm)
**4b**	368	440	**5b**	363	450
**4c**	370	430	**5c**	373	470
**4d**	380	455	**5d**	356	440
**4f**	363	420	**8a**	305	355
**4g**	350	415	**8c**	300	355
**4i**	374	445	**8d**	327	415
**5a**	334	390	**9**	267, 360	450

Figure S6. Absorption spectra of: (**A**) enaminones **4b–d,f,g,i**, (**B**) β-ketoiminate complexes **5a–d**, (**C**) *N*-Boc-enaminones **8a,c,d**, and (**D**) compound **9**.

5. References

1. CrysAlis PRO, Agilent Technologies UK Ltd, Yarnton, Oxfordshire, England, **2011**.
2. Dolomanov, O. V.; Bourhis, L. J.; Gildea, R. J.; Howard, J. A. K.; Puschmann, H. *OLEX2*: a complete structure solution, refinement and analysis program. *J. Appl. Cristallogr.* **2009**, *42*, 339–341. DOI: https://doi.org/10.1107/S0021889808042726
3. Sheldrick, G. M. A short history of *SHELX. Acta Crystallogr. A* **2008**, *64*, 112–122. DOI: https://doi.org/10.1107/S0108767307043930
4. Sheldrick, G. M. *SHELXT* – Integrated space-group and crystal-structure determination.*Acta Crystallogr. Sect. Found. Adv.* **2015**, *71*, 3–8. DOI: https://doi.org/10.1107/S2053273314026370
5. Sheldrick, G. M. Crystal structure refinement with *SHELXL*. *Acta Crystallogr. Sect. C Struct. Chem.* **2015**, *71*, 3–8. DOI: https://doi.org/10.1107/S2053229614024218
6. Macrae, C. F.; Edgington, P. R.; McCabe, P.; Pidcock, E.; Shields, G. P.; Taylor, R.; Towler, M.; van de Streek, J. Mercury: visualization and analysis of crystal structures. *J. Appl. Crystallogr.* **2006**, *39*, 453–457. DOI: https://doi.org/10.1107/S002188980600731X
7. Spek, A. L. Single-crystal structure validation with the program *PLATON*. *J. Appl. Crystallogr.* **2003**, *36*, 7–13. DOI: https://doi.org/10.1107/S0021889802022112
